# Impact of Side Chain Structure and Aglycon Carbonyl Group on the Immunostimulatory Activities of Semisynthetic Saponin Adjuvants

**DOI:** 10.1021/acsptsci.5c00792

**Published:** 2026-02-20

**Authors:** Di Bai, Liz Wang, Rebekah Beyea, Hyunjung Kim, Pengfei Wang

**Affiliations:** † Department of Chemistry, 9968 University of Alabama at Birmingham, 901 14th Street South, Birmingham, Alabama 35294, United States; ‡ Adjuvax LLC, 2000 9th Avenue South, Birmingham, Alabama AL35205, United States

**Keywords:** vaccine adjuvant, saponin immunostimulant, *Momordica* saponins, VSA-1, VSA-2

## Abstract

VSA-1 and VSA-2 are recently developed semisynthetic saponin immunostimulants derived from natural *Momordica* saponins (MS) I and II, respectively, through the incorporation of a linear dodecyl or benzyl undecanoate side chain. In this study, a series of novel MS derivatives with systematically varied side-chain lengths and steric bulk were synthesized. Immunological evaluation revealed that for MS I derivatives, adjuvant activity increased with side-chain elongation from C8 to C12 but declined upon further extension to C16. Enhancement of side-chain bulk, achieved by substituting the linear primary amide with a secondary amide, resulted in diminished adjuvant activity. In the case of MS II derivatives, either elongation or shortening of the side chain relative to –(CH_2_)_10_COOBn led to loss of immunostimulatory function, as evidenced by reduced IgG1 and IgG2a production. Furthermore, for both VSA-1 and VSA-2, reduction of the C23 carbonyl group to a primary hydroxyl group completely abolished the adjuvant activity.

## Graphical abstract

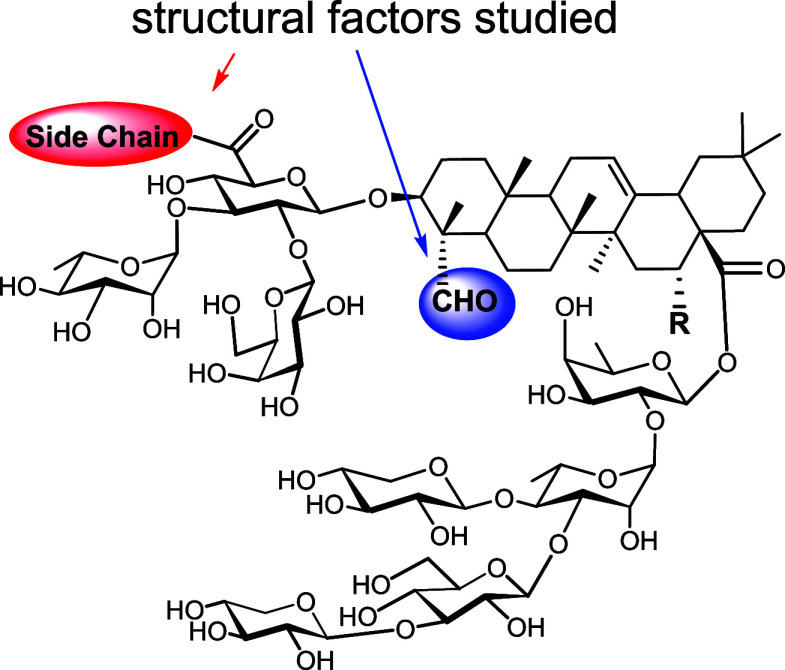

Saponin-based immunostimulants have attracted increasing attention owing to the clinical success of QS-21, a natural saponin adjuvant isolated from the bark of *Quillaja saponaria* Molina (QS), an evergreen tree native to central Chile. QS-21 elicits robust and balanced humoral and cellular immune responses and has been incorporated into the shingles vaccine *Shingrix*, the malaria vaccine *Mosquirix*, and the respiratory syncytial virus (RSV) vaccine *Arexvy* as a component of the combination adjuvants AS01_B_ and AS01_E_.^1,2^ Another QS-21-containing adjuvant, Matrix-M, has been approved for use in a human COVID-19 vaccine as well as the malaria vaccine R21. Despite the long-standing success of saponins as vaccine adjuvants, the molecular basis of their immunostimulatory activity remains incompletely understood. Elucidating the structure–activity relationships (SAR) of saponins has therefore been the focus of extensive research aimed at identifying the structural motifs essential for adjuvant activity.^3–23^

For QS-21 (Figure 1), the contributions of individual structural elements, including the C3-branched trisaccharide, the C28-linear tetrasaccharide, the dimeric normonoterpene carboxylic acid moiety linked to the fucosyl residue of the C28 tetrasaccharide, the C23 carbonyl group of the quillaic acid core, and the anomeric stereochemistry, have been investigated across various analogs. Nevertheless, the structure–activity relationships underlying the adjuvant activity of saponins remain only partially elucidated.

**Figure 1:**
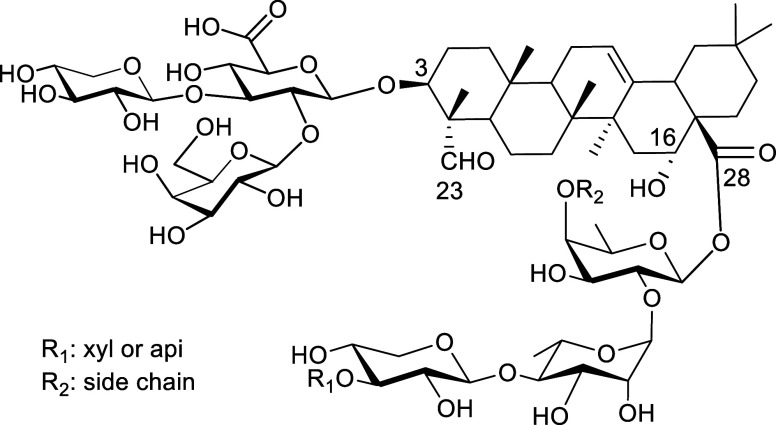
Structure of QS-21.

Subsequent SAR studies of QS saponins have led to the discovery of a new generation of semisynthetic saponin adjuvants, VSA-1 and VSA-2, which can be conveniently synthesized in a single step from *Momordica* saponins.^24–26^ These natural precursors are sustainably sourced from the seeds of the widely available perennial plant *Momordica cochinchinensis* Spreng (Figure 2).^27^ Both VSA-1 and VSA-2 have demonstrated promising adjuvant activities in direct, head-to-head comparisons with QS-21.^28–30^ In this study, we further investigated the SAR of the VSA series to elucidate the structural determinants responsible for their adjuvant activity.

**Figure 2:**
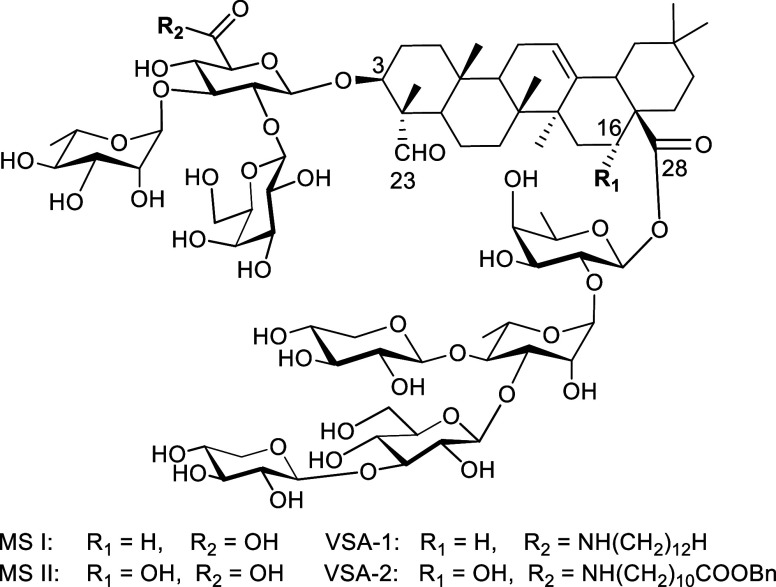
Structure of natural *Momordica* saponins I and II and semisynthetic VSA-1 and VSA-2.

## Results and Discussion

Earlier studies have demonstrated that the acyl side chain of QS-21 is responsible for both its toxicity and its ability to stimulate cellular immune responses.^6,31^ Removal of the acyl side chain from QS saponins abolishes their capacity to induce antigen-specific lymphoproliferation and cytotoxic T lymphocyte (CTL) responses. Reintroduction of a simple aliphatic side chain into deacylated QS saponins, achieved through chemical coupling of dodecylamine to the glucuronic acid moiety at the C3 position of quillaic acid, produced a complex mixture of semisynthetic QS saponin analogs collectively known as GPI-0100. This preparation restored potent immunostimulatory properties, inducing both Th1- and Th2-type responses as well as antigen-specific CTL activation, comparable to those elicited by QS-21.^4,6^ Marciani and co-workers demonstrated that shorter side chains (C8–C10) produced effects similar to those of the dodecyl side chain; however, extension of the chain beyond 12 carbons—such as incorporation of a tetradecylamide (C14) chain—resulted in an IgG subclass distribution resembling that of deacylated saponins, characterized by a Th2-biased response, as indicated by reduced IgG2a/IgG1 titer ratios.^6,18^

Despite the established importance of the fatty side chain in modulating immune responses, its molecular mechanism of action remains unclear. Previous investigations into the influence of side-chain length on the adjuvant activity of QS saponin analogs were conducted using complex mixtures, making it difficult to attribute the observed effects to specific molecular species. Thus, it is of interest to determine whether similar structure–activity trends can be observed using a structurally well-defined pure saponin. VSA-1 represents such a structurally defined semisynthetic saponin, prepared from pure *Momordica* saponin I via the same synthetic strategy employed in the preparation of GPI-0100.^24^

To explore the role of side-chain length and steric effects, six VSA-1 analogs (compounds **1**–**6**, Figure 3) were synthesized. Compounds **1**–**4** contain linear aliphatic side chains of varying lengths, C8, C10, C14, and C16, respectively. Previous SAR studies of QS-21 by Kensil and co-workers suggested that the carboxyl group of the C3-glucuronic acid is not directly involved in adjuvant function; however, an increase in side-chain bulk may introduce steric hindrance that interferes with a site critical to biological activity.^4^ To assess the effect of steric factors, two additional analogs (compounds **5** and **6**) were synthesized, each featuring a 12-carbon aliphatic moiety similar to that in VSA-1. Compound **5** contains a branched *N*,*N*-dihexylamide side chain, isomeric to the linear dodecylamide of VSA-1, while compound **6** incorporates a cyclic aliphatic moiety through the attachment of an azacyclotridecyl unit.

**Figure 3:**
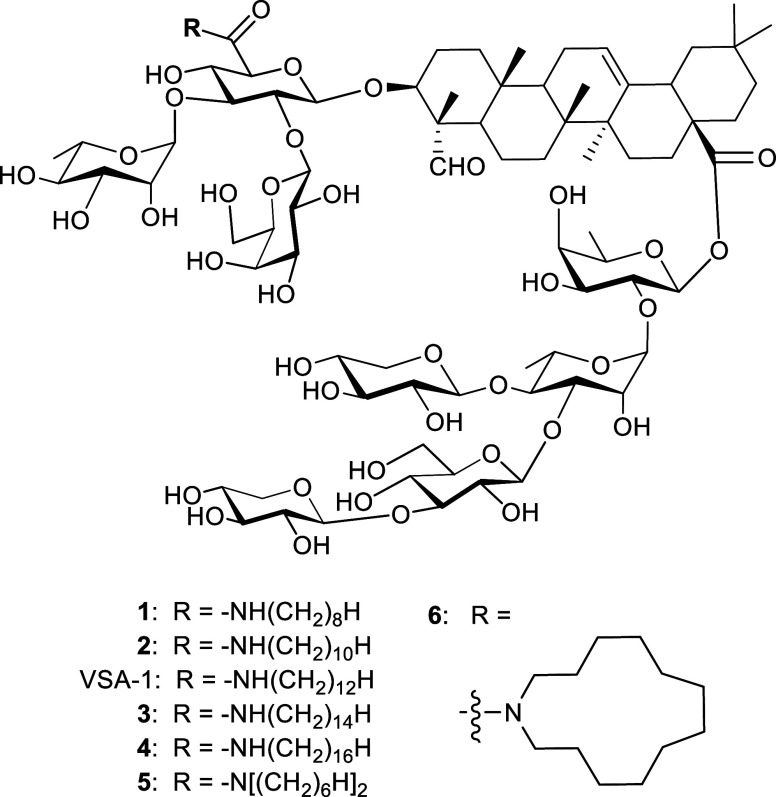
VSA-1 analogs.

The natural saponin precursors, MS I and II, were isolated from the seeds of *M. cochinchinensis* (Lour.) Spreng according to a previously published method.^27^ The VSA-1 analogs **1**–**6** were then synthesized via a straightforward, one-step amide-coupling reaction.^32^

The immunostimulatory activities of VSA-1 and its analogs **1**–**6** were evaluated by measuring their ability to enhance antibody responses to chicken egg ovalbumin (OVA). Female BALB/c mice (8 to 10 weeks old, five per group) were immunized subcutaneously (s.c.) with OVA (20 μg) alone or in combination with VSA-1 (100 μg) or saponins **1**–**6** (100 μg) on days 0, 14, and 28. Serum samples were collected, and body weights were recorded prior to each immunization and 2 weeks after the final immunization. Antigen-specific serum IgG1 and IgG2a titers were determined by enzyme-linked immunosorbent assay (ELISA). Because IgG1 and IgG2a production is preferentially enhanced by Th2- and Th1-type cytokines, respectively, their relative levels serve as reliable indicators of the Th2/Th1 immune bias induced by each adjuvant.^33^

With increasing side-chain length from C8 to C12, both IgG1 and IgG2a titers increased in 2 weeks after the second (week 4) and third (week 6) immunizations (Figure 4). However, further extension of the side chain beyond C12, as in C14 and C16 analogs, resulted in reduced antibody responses, consistent with observations from QS-derived analogs. None of these longer-chain analogs elicited IgG2a levels comparable to those induced by VSA-1. Similarly, analogs **5** and **6**, which feature either a branched side chain or a cyclic amide moiety with the same carbon count as the dodecyl chain of VSA-1, did not induce antibody titers significantly higher than those observed with OVA alone, especially IgG2a (Figure S1). These results confirm that a linear dodecyl side chain is critical for the adjuvant activity of MS I-derived saponins.

**Figure 4:**
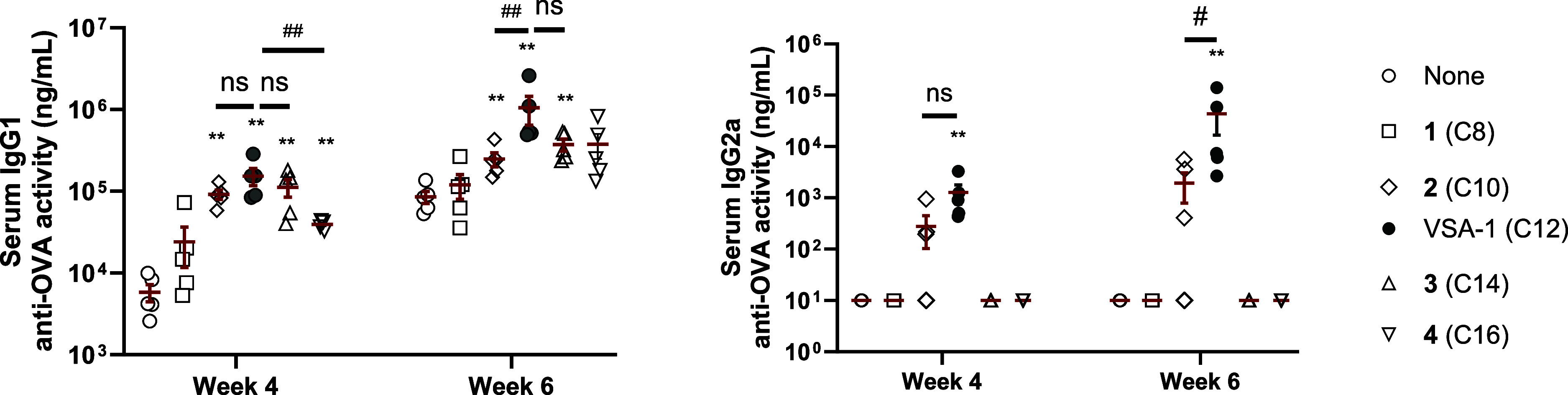
Serum IgG1 and IgG2a anti-OVA response in mice immunized by the s.c. route with OVA alone or with VSA-1 or a VSA-1 analog. Mice were immunized on days 0, 14, and 28. Serum samples were collected prior to each immunization and at 6 weeks after the initial immunization. Values are expressed as mean ± SEM. Statistical significance in antibody responses was evaluated by *t* tests (with unpaired, nonparametric, and Mann–Whitney test). *P < 0.05 and **P < 0.01 compared with mice immunized with OVA alone; #*P* < 0.05 and ##*P* < 0.01.

A recent SAR study of QS-21 analogs focusing on the chain length of the introduced carboxyl side chain demonstrated that analogs with chains two or four carbons longer than that of TQL1055 (Figure 5) exhibit adjuvant activity comparable to QS-21 and superior to TQL1055.^22^

**Figure 5:**
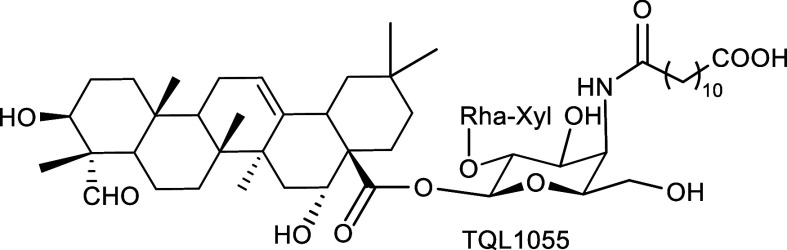
Structure of QS-21 analog TQL1055.

Given that VSA-2 possesses a carboxyl ester side chain of similar length to TQL1055, it was of interest to determine whether the side-chain length similarly influences its adjuvant activity. Accordingly, we synthesized analogs **7**–**10** with varying chain lengths (Figure 6). In addition, compound **11** contains a benzyl ester of a dimeric aminopentanoic acid, whereas compound **12** incorporates an ester of the trimeric analog. These two analogs will reveal whether internal functional group(s) of a side chain would affect adjuvant activities.

**Figure 6:**
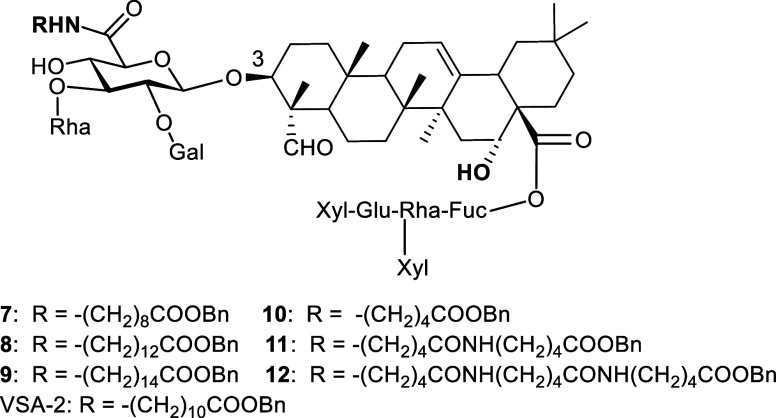
Structure of VSA-2 analogs.

The VSA-2 analogs were synthesized using a procedure analogous to that employed for VSA-2 (Scheme 1).^30^ The starting materials, 9-aminodecanoic acid, 13-aminotridecanoic acid, and 15-aminopentadecanoic acid, are commercially available and were converted to the corresponding benzyl esters via reaction with thionyl chloride in benzyl alcohol. Coupling of these benzyl ester side chains with MS II yielded analogs **7**–**9**. The side chain for analogue **10** is commercially available, and its incorporation was achieved by coupling MS II with benzyl 5-aminopentanoate hydrochloride (**13**). The synthesis of analogs **11** and **12**, which feature dimeric and trimeric side chains mimicking QS-21, was accomplished through a stepwise strategy. Coupling of commercially available *N*-Boc-5-aminopentanoic acid (**14**) with **13** afforded the dimeric intermediate **15**, while coupling of **14** with **15** generated the trimeric intermediate **16**. Subsequent incorporation of intermediates **15** and **16** into MS II yielded analogs **11** and **12**, respectively.

**Scheme 1:**
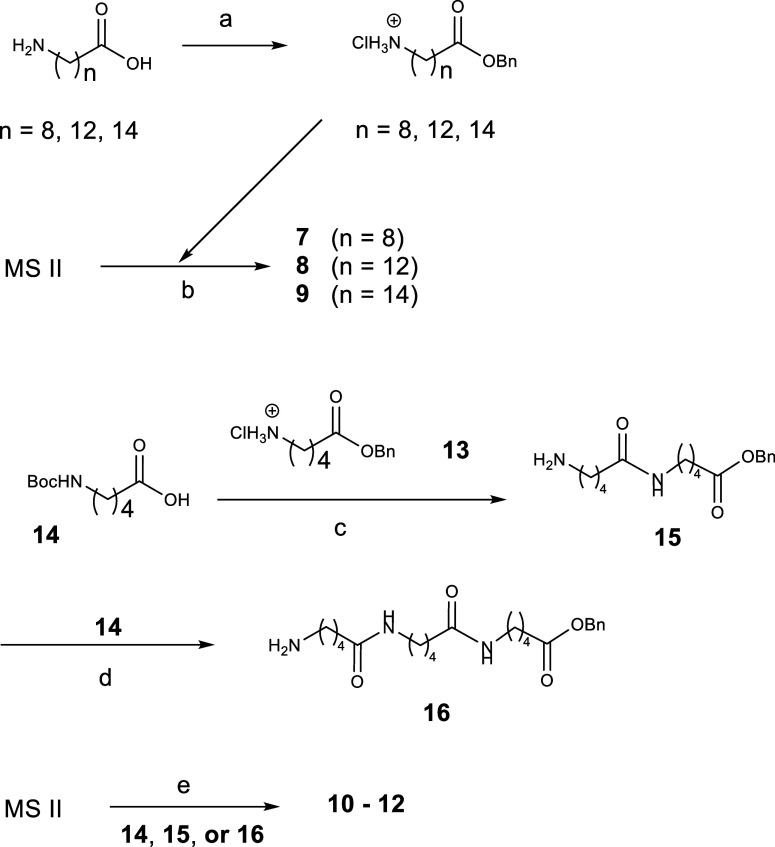
Synthesis of VSA-2 Analogs with Different Side Chains^a^

a Reagents and conditions: (a) SOCl_2_, BnOH, r.t.-70 °C, 27% (*n* = 8), 99% (*n* = 12), 93% (*n* = 14); (b) *N*-methylmorpholine (NMM), hydroxybenzotriazole (HOBt), 1-ethyl-3-(3-(dimethylamino)­propyl)-carbodiimide hydrochloride (EDC·HCl), MeCN/H_2_O, r.t., 92% (*n* = 8), 71% (*n* = 12), 69% (*n* = 14); (c) NMM, HOBt, EDC·HCl, DCM, r.t., 22%; TFA, DCM, r.t., 85%; (d) NMM, HOBt, EDC·HCl, DCM, r.t., 36%; TFA, DCM, r.t., 100%; (e) NMM, HOBt, EDC·HCl, MeCN/H_2_O, r.t., 53% (**10**), 83 (**11**), 64% (**12**).

The immunostimulatory activities of VSA-2 analogs were evaluated by measuring the responses of the OVA-specific IgG1 and IgG2a responses. Female BALB/c mice (8–10 weeks old, five per group) were immunized subcutaneously (s.c.) with OVA (20 μg) alone or in combination with QS-21 (20 μg), VSA-2 (100 μg), or saponins **7**–**12** (100 μg), following the same immunization and bleeding schedule used for VSA-1 analogs (**1**–**6**). ELISA analysis demonstrated that only QS-21 and VSA-2 induced IgG1 and IgG2a titers significantly higher than those of the group induced by the OVA alone in weeks 4 and 6. Alterations in side-chain length or incorporation of an internal amide group substantially diminished adjuvant activity (Figure 7).

**Figure 7:**
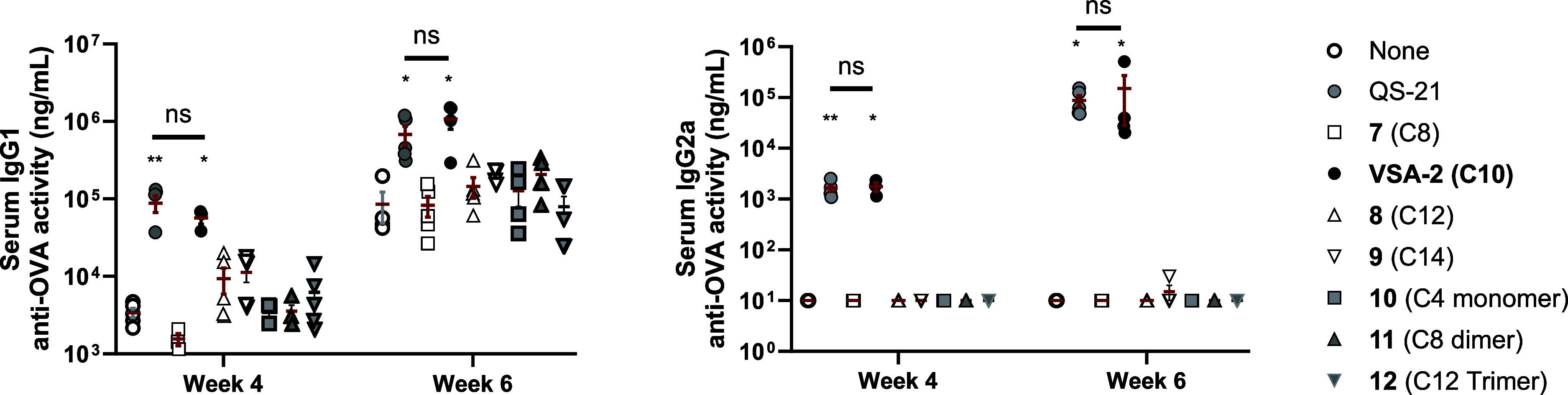
Serum IgG1 and IgG2a anti-OVA response in mice immunized by the s.c. route with OVA alone or with QS-21, VSA-2, or a VSA-2 analog. Mice were immunized on days 0, 14, and 28. Serum samples were collected prior to each immunization and at 6 weeks after the initial immunization. Values are expressed as mean ± SEM. Statistical significance in antibody responses was evaluated by *t* tests (with unpaired, nonparametric, and Mann–Whitney test). *P < 0.05 and **P < 0.01 compared with mice immunized with OVA alone.

The C23 carbonyl group of quillaic acid in QS-21 is known to be critical for adjuvanticity; however, its precise immunological role remains unclear. Kensil and co-workers observed that modification of this carbonyl abolished QS-21’s adjuvant function, eliminating its ability to stimulate antibody production and induce CTL.^4^ It was proposed that the carbonyl group may participate in a Schiff base interaction with free amino groups on the surface of the target immune cells. Nonetheless, direct Schiff base-stabilized interaction between QS-21 and a specific immune cell population has not yet been demonstrated.

However, in a study by Oda and co-workers examining 47 saponins isolated from medicinal and food plants, the C23 aldehyde group in the aglycone was found to be nonessential for adjuvant activity.^7^ Similarly, Palatnik-de-Sousa et al. reported that one of the *Chiococca alba* saponins, CA4 (Figure 8A)—a triterpene bidesmoside with an oleanane-type triterpene core lacking a carbonyl moiety—elicited robust cellular immune responses, significantly enhancing antigen-specific IgG and IgG2a production and increasing CD4^+^ TNF-α, CD8^+^ IFN-γ, and CD8^+^ TNF-α levels.^11^ In a SAR study of synthetic QS-21 analogs, Fernandez-Tejada et al. demonstrated that the quillaic acid variant TQL1055 (bearing a C23 carbonyl) produced lower IgG1 and IgG2b titers compared with its echinocystic acid counterpart analog EA, which lacks a C23 carbonyl (Figure 8B).^16^

**Figure 8:**
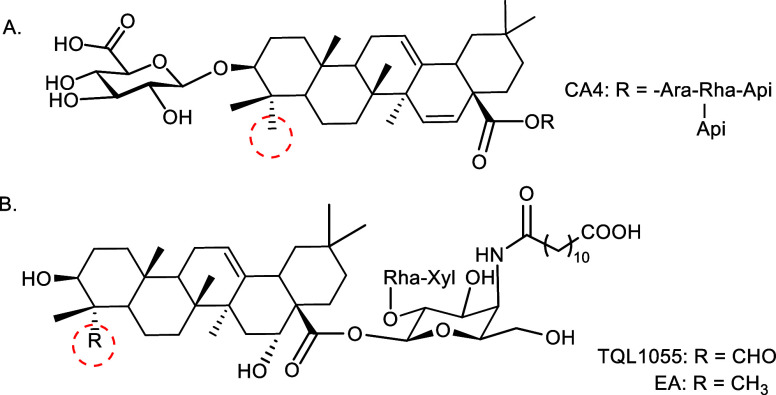
(A) *Chiococca alba* saponin CA4 and (B) synthetic QS-21 analogs.

To evaluate the contribution of the C23 carbonyl group to adjuvant activity, two analogs, V1H and V2H, were synthesized (Scheme 2). V1H was obtained via the direct reduction of VSA-1 with NaBH_4_. For V2H, reduction was first performed on MS II, followed by amide coupling with benzyl 11-aminoundecanoate. Immunological evaluation, using the same immunization, sampling, and ELISA procedures applied to the other analogs, demonstrated that reduction of the C23 carbonyl in VSA-1 and VSA-2 abolished their ability to enhance both IgG1 and IgG2a production (Figure S2).

**Scheme 2:**
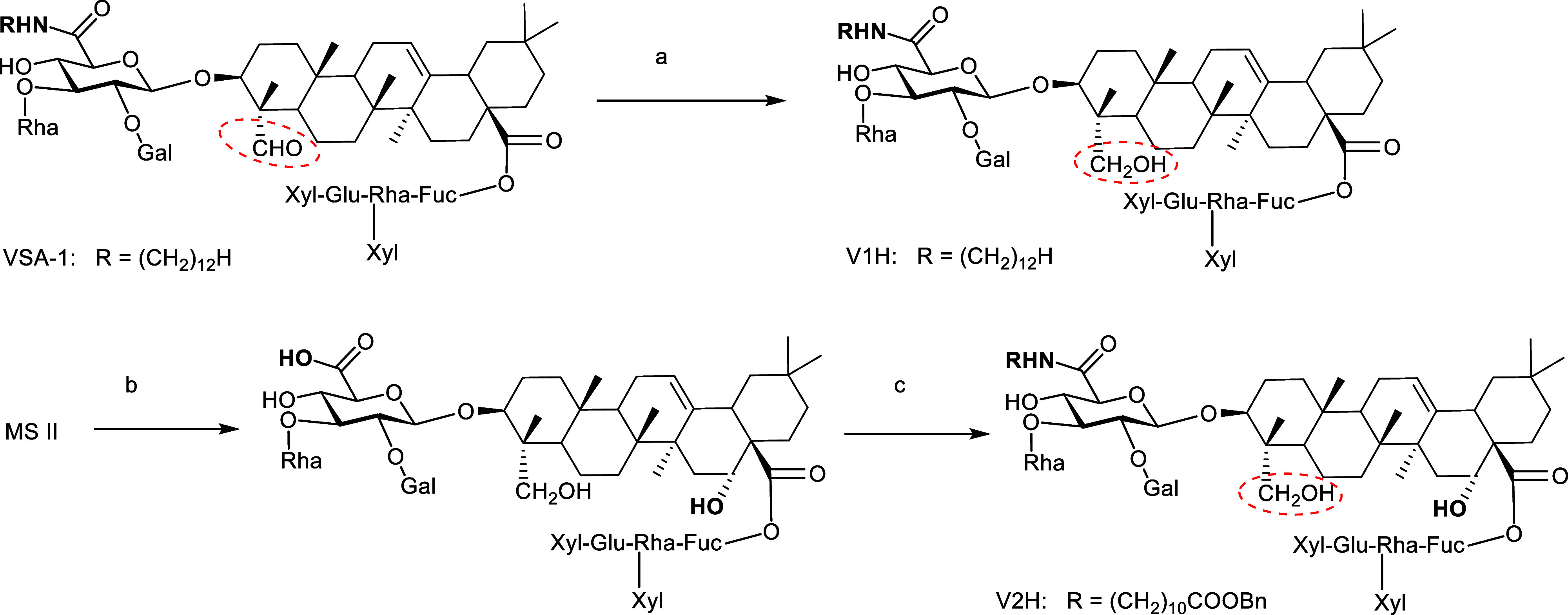
Synthesis of VSA Analogs with Reduced C23^a^

a Reagents and conditions: (a) NaBH_4_, MeOH, 0 °C-r.t., 83%; (b) NaBH_4_, MeOH, 0 °C-r.t., 100%; (c) Benzyl 11-aminoundecanoate, NMM, HOBt, HBTU, MeCN/H_2_O, r.t., 37%.

## Conclusion

In this work, we demonstrate that for MS I bearing a gypsogenin-type triterpenoid core incorporation of a linear dodecylamide side chain at the C3-glucuronic acid position yields a semisynthetic saponin exhibiting the highest immunostimulatory activity. Shortening or lengthening the side chain reduces both antigen-specific IgG1 and IgG2a responses, consistent with observations reported for QS-derived analogs. Introduction of a branched or cyclic 12-carbon aliphatic moiety similarly diminishes the adjuvant activity of the resulting saponins, supporting the earlier postulate by Kensil and co-workers that increased steric hindrance at the C3-glucuronic acid may interfere with interactions critical to biological activity. Recent investigations on QS-21 analogs have shown that extending the terminal-functionalized side chain (bearing a terminal carboxyl group) from 12 to 14 or 16 carbons enhances adjuvant potency.^22^ VSA-2, derived from MS II, also features a terminal-functionalized side chain (with a terminal benzyl ester group).^25^ Our results indicate that the adjuvant activity of VSA-2 is more sensitive to side-chain length, as either elongation or shortening of the chain markedly reduces the activity. The role of the C23 carbonyl group in saponin adjuvants remains incompletely understood; however, for both VSA-1 and VSA-2, reduction of the triterpenoid C23 carbonyl to a primary hydroxyl group abolishes adjuvant activity, as evidenced by ELISA analyses of antigen-specific IgG1 and IgG2a production. The results from this work along with other SAR studies confirm that saponins’ adjuvanticity is sensitive to structural variation,^4,6,13,17,18,20,22,34^ even when the structural changes do not affect their hydrophilic–lipophilic balance.^30^

## Experimental Section

### Chemistry General

Organic solutions were concentrated by rotary evaporation at a rate of about 12 Torr. Flash column chromatography was performed by employing 230–400 mesh silica gel. Thin-layer chromatography was performed using glass plates precoated to a depth of 0.25 mm with 230–400 mesh silica gel impregnated with a fluorescent indicator (254 nm). Proton and carbon-13 nuclear magnetic resonance (^1^H NMR or ^13^C NMR) spectra were recorded on 300, 500, 600, and 850 MHz NMR spectrometers. Chemical shifts are expressed in parts per million (δ scale) downfield from tetramethylsilane. Data are presented as follows: chemical shift, multiplicity (s = singlet, d = doublet, t = triplet, q = quartet, m = multiplet and/or multiple resonances, AB = AB quartet), coupling constant in Hertz (Hz), integration. Anhydrous solvents were used without distillation. Solvents for workup and column chromatography were obtained from commercial vendors and used without further purification. The purity of the products was determined by a combination of HPLC, HRMS, and ^1^H NMR and found to be ≥95%.

### General Procedure of Preparing Side Chains

A mixture of an amino fatty acid (0.25 mmol) and thionyl chloride (36 μL, 0.5 mmol) was first stirred at room temperature and then was heated to 70 °C in an oil bath and stirred for about 1 h. To the reaction mixture was added substituted benzyl alcohol (0.5 mmol), and the mixture was stirred overnight. The mixture was purified with column chromatography on silica (eluted with a DCM/MeOH gradient) to produce the desired side chain.

### General Procedure of Derivatizing MS I and II

*Momordica* saponins were isolated by using the published procedure.^27^ To a clear solution of MS I (15.8 mg, 9.4 μmol) in acetonitrile (0.5 mL) and water (0.25 mL) were added 11-aminoundecanoic acid benzyl ester hydrochloride (6.6 mg, 20 μmol),^35^ *N*-methylmorpholine (NMM) (12.0 mg, 118 μmol), hydroxybenzotriazole (HOBt) (9.2 mg, 60 μmol), and 1-ethyl-3-(3-(dimethylamino)­propyl)­carbodiimide hydrochloride (EDC·HCl) (12.0 mg, 63 μmol) at room temperature.^32^ The reaction mixture was stirred for 1 day and then filtered. The filtrate was purified with RP HPLC by using a semi-Prep C18, 250 × 10 mm, 5 μm column, and H_2_O/MeCN gradients (90–10% H_2_O over 45 min with a 3 mL/min flow rate). The desired product had a retention time of 30 min, and the fraction was concentrated on a rotary evaporator at room temperature to remove MeCN; the remaining water was then removed on a lyophilizer to provide the final product as a white solid.

### **1** (9.6 mg, 30%)

^1^H NMR (850 MHz, CD_3_OD) (characteristic protons): δ 9.48 (s, 1H), 5.35 (d, *J* = 1.6 Hz, 1H), 5.34 (d, *J* = 8.3 Hz, 1H), 5.27 (t, *J* = 3.6 Hz, 1H), 5.04 (d, *J* = 1.5 Hz, 1H), 4.66 (d, *J* = 7.8 Hz, 1H), 4.59 (d, *J* = 7.8 Hz, 1H), 4.51–4.48 (m, 2H), 4.47 (m, 1H), 4.27 (dd, *J* = 3.0, 1.8 Hz, 1H), 4.03 (dd, *J* = 3.32, 1.9 Hz, 1H), 4.00 (dd, *J* = 11.5, 5.4 Hz, 1H), 3.15 (t, *J* = 10.9 Hz, 1H), 3.06 (dd, *J* = 9.2, 7.6 Hz, 1H), 2.82 (dd, *J* = 13.8, 4.1 Hz, 1H), 1.20 (s, 3H), 1.19 (s, 3H), 1.02 (s, 3H), 0.96–0.81 (m, 10H), 0.82 (s, 3H); ^13^C NMR (580 MHz, CD_3_OD): δ 209.2, 176.6, 169.9, 169.8, 143.5, 121.8, 104.6, 103.9, 103.7, 102.8, 102.4, 101.8, 100.0, 94.0, 87.3, 84.3, 84.1, 81.5, 77.7, 76.7, 76.1, 76.0, 75.4, 74.9, 74.5, 74.0, 73.6, 73.0, 72.8, 72.4, 72.2, 71.6, 71.3, 70.8, 70.7, 70.4, 70.1, 70.0, 69.6, 69.2, 69.1, 68.1, 67.4, 65.7, 65.6, 60.8, 60.8, 58.4, 58.3, 56.1, 54.8, 48.1, 48.0, 46.6, 46.0, 41.9, 41.6, 39.6, 38.9, 38.8, 37.9, 35.5, 33.5, 32.2, 32.1, 31.7, 31.5, 30.1, 29.2, 29.1, 28.9, 28.9, 27.5, 26.6, 24.7, 24.4, 23.1, 22.8, 22.6, 22.4, 20.2, 17.0, 16.4, 16.4, 15.1, 14.9, 13.1, 9.5; HRMS (ESI-TOF) *m*/*z*: [M + H]^+^ calcd for C_84_H_138_NO_39_, 1784.8846; found, 1784.8883.

### **2** (13.3 mg, 41%)

^1^H NMR (850 MHz, CD_3_OD) (characteristic protons): δ 9.48 (s, 1H), 5.35 (d, *J* = 1.6 Hz, 1H), 5.34 (d, *J* = 8.3 Hz, 1H), 5.27 (t, *J* = 3.6 Hz, 1H), 5.04 (d, *J* = 1.5 Hz, 1H), 4.66 (d, *J* = 7.8 Hz, 1H), 4.59 (d, *J* = 7.8 Hz, 1H), 4.51–4.48 (m, 2H), 4.47 (m, 1H), 4.27 (dd, *J* = 3.0, 1.8 Hz, 1H), 4.03 (dd, *J* = 3.3, 1.9 Hz, 1H), 4.00 (dd, *J* = 11.5, 5.4 Hz, 1H), 3.15 (t, *J* = 10.9 Hz, 1H), 3.06 (dd, *J* = 9.2, 7.6 Hz, 1H), 2.82 (dd, *J* = 13.8, 4.1 Hz, 1H), 1.20 (s, 3H), 1.19 (s, 3H), 1.02 (s, 3H), 0.96–0.81 (m, 10H), 0.82 (s, 3H); ^13^C NMR (580 MHz, CD_3_OD): δ 209.2, 176.5, 169.8, 143.5, 121.8, 104.6, 103.9, 103.7, 102.8, 102.4, 101.8, 100.0, 94.0, 87.3, 84.3, 84.1, 81.5, 77.7, 77.6, 76.6, 76.1, 75.6, 75.4, 74.9, 74.5, 74.0, 73.6, 73.0, 72.8, 72.4, 72.2, 71.6, 71.3, 70.8, 70.7, 70.4, 70.1, 70.0, 69.6, 69.2, 69.1, 68.1, 67.4, 65.7, 65.6, 60.8, 60.8, 58.4, 58.3, 58.2, 56.2, 56.1, 56.0, 54.8, 48.1, 46.6, 46.0, 41.8, 41.6, 39.6, 39.0, 38.7, 38.0, 35.7, 33.5, 32.2, 32.1, 31.7, 31.7, 31.5, 30.1, 29.5, 29.4, 29.4, 29.2, 29.2, 29.0, 29.0, 28.9, 27.5, 26.5, 24.7, 24.4, 23.2, 22.8, 22.6, 22.4, 22.3, 21.1, 21.0, 20.2, 17.0, 16.4, 16.4, 16.1, 16.0, 15.9, 15.8, 15.1, 15.0, 13.1, 13.0, 11.9, 9.5; HRMS (ESI-TOF) *m*/*z*: [M + H]^+^ calcd for C_84_H_138_NO_39_, 1784.8846; found, 1784.8883.

### **3** (14.3 mg, 43%)

^1^H NMR (850 MHz, CD_3_OD) (characteristic protons): δ 9.48 (s, 1H), 5.35 (d, *J* = 1.6 Hz, 1H), 5.33 (d, *J* = 8.3 Hz, 1H), 5.27 (t, *J* = 3.7 Hz, 1H), 5.04 (d, *J* = 1.5 Hz, 1H), 4.66 (d, *J* = 7.8 Hz, 1H), 4.58 (d, *J* = 7.9 Hz, 1H), 4.52–4.48 (m, 2H), 4.47 (m, 1H), 4.27 (dd, *J* = 3.2, 1.9 Hz, 1H), 4.03 (dd *J* = 3.3, 1.9 Hz, 1H), 4.00 (dd, *J* = 9.2, 5.4 Hz, 1H), 3.15 (t, *J* = 10.9 Hz, 1H), 3.06 (t, *J* = 8.8 Hz, 1H), 2.83 (dd, *J* = 13.6, 3.7 Hz, 1H), 1.23 (s, 3H), 1.19 (s, 3H), 1.11 (t, *J* = 7.2 Hz, 2H), 1.02 (s, 3H), 0.82 (s, 3H); ^13^C NMR (580 MHz, CD_3_OD): δ 209.3, 176.5, 169.8, 143.6, 121.8, 117.6, 116.2, 104.6, 103.9, 103.7, 102.8, 102.5, 101.8, 100.0, 94.0, 87.3, 84.3, 84.1, 81.5, 77.7, 77.6, 76.8, 76.6, 76.1, 76.0, 75.4, 75.4, 74.9, 74.5, 74.0, 73.6, 73.0, 72.8, 72.4, 72.2, 71.6, 71.3, 70.8, 70.7, 70.4, 70.1, 70.0, 69.6, 69.2, 69.1, 68.1, 67.4, 65.7, 65.6, 60.8, 60.8, 56.5, 54.8, 46.6, 46.0, 43.7, 41.8, 41.6, 39.6, 39.0, 38.7, 38.0, 37.6, 35.7, 34.4, 33.5, 32.2, 32.1, 31.7, 31.5, 30.1, 29.53, 29.46, 29.45, 29.43, 29.38, 29.2, 29.1, 28.9, 27.5, 27.4, 26.6, 24.8, 24.4, 23.2, 22.8, 22.6, 22.4, 20.2, 17.0, 16.4, 16.4, 15.1, 15.0, 14.4, 13.1, 9.5; HRMS (ESI-TOF) *m*/*z*: [M + H]^+^ calcd for C_90_H_150_NO_39_, 1868.9785; found, 1868.9716.

### **4** (14 mg, 41%)

^1^H NMR (850 MHz, CD_3_OD) (characteristic protons): δ 9.48 (s, 1H), 5.35 (d, *J* = 1.5 Hz, 1H), 5.34 (d, *J* = 8.3 Hz, 1H), 5.27 (t, *J* = 3.9 Hz, 1H), 5.04 (d, *J* = 1.5 Hz, 1H), 4.66 (d, *J* = 7.8 Hz, 1H), 4.58 (d, *J* = 7.8 Hz, 1H), 4.52–4.82 (m, 2H), 4.47 (m, 1H), 4.27 (dd, *J* = 1.9, 3.1 Hz, 1H), 4.03 (dd, *J* = 1.8, 3.2 Hz, 1H), 4.00 (dd, *J* = 5.4, 11.5 Hz, 1H), 3.15 (t, *J* = 11.3 Hz, 1H), 3.06 (dd, *J* = 8.0, 9.2 Hz, 1H), 2.83 (dd, *J* = 4.0, 13.7 Hz, 1H), 1.28 (d, *J* = 6.2 Hz, 3H), 1.20 (s, 3H), 1.19 (s, 3H), 1.02 (s, 3H), 0.94 (s, 3H), 0.82 (s, 3H); ^13^C NMR (850 MHz, CD_3_OD): δ 209.3, 176.5, 169.8, 161.9, 161.7, 161.6, 161.4, 121.8, 118.9, 117.5, 116.2, 114.8, 104.6, 103.9, 103.7, 102.8, 102.5, 101.8, 100.0, 94.0, 87.3, 84.3, 84.1, 81.5, 77.7, 77.6, 76.8, 76.6, 76.1, 76.0, 75.4, 75.4, 74.9, 74.5, 74.0, 73.6, 73.0, 72.8, 72.4, 72.2, 71.6, 71.3, 70.8, 70.7, 70.4, 70.1, 70.0, 69.6, 69.2, 69.1, 68.1, 67.4, 65.7, 65.6, 60.8, 60.8, 54.9, 48.1, 46.6, 46.0, 41.8, 41.6, 39.6, 38.7, 38.0, 35.7, 33.5, 32.2, 32.1, 31.7, 31.6, 30.1, 29.5, 29.5, 29.4, 29.4, 29.2, 29.1, 28.9, 27.5, 26.5, 24.8, 24.4, 23.2, 22.8, 22.6, 22.4, 20.2, 17.0, 16.4, 16.4, 15.1, 15.0, 13.1, 9.5; HRMS (ESITOF) *m*/*z*: [M + H]^+^ calcd for C_92_H_154_NO_39_, 1897.0098; found, 1897.0055.

### **5** (10 mg, 36%)

^1^H NMR (500 MHz, CD_3_OD) (characteristic protons): δ 9.48 (s, 1H), 5.33 (d, *J* = 1.6 Hz, 1H), 5.31 (d, *J* = 8.2 Hz, 1H), 5.26 (t, *J* = 3.3 Hz, 1H), 5.03 (d, *J* = 1.0 Hz, 1H), 4.64 (d, *J* = 7.8 Hz, 1H), 4.58 (d, *J* = 7.6 Hz, 1H), 4.50–4.48 (m, 2H), 4.47 (m, 1H), 4.26 (dd, *J* = 3.0, 1.8 Hz, 1H), 4.14 (d, *J* = 9.3 Hz, 1H), 4.00 (dd, *J* = 3.2, 1.8 Hz, 1H), 3.91 (dd, *J* = 11.4, 5.3 Hz, 1H), 3.17 (q, 1.7 Hz, 1H), 3.15 (t, *J* = 10.9 Hz, 1H), 3.06 (dd, *J* = 9.2, 7.9 Hz, 1H), 2.82 (dd, *J* = 13.8, 3.9 Hz, 1H), 1.38 (m, 6H), 1.31 (m, 10H), 1.00 (s, 3H), 0.95 (m, 3H), 0.92 (s, 3H), 0.91 (s, 3H), 0.80 (s, 3H); ^13^C NMR (176 MHz, CD_3_OD): δ 209.6, 176.5, 168.2, 162.0, 161.8, 161.6, 143.5, 121.8, 118.8, 117.4, 116.0, 114.7, 104.6, 103.9, 103.7, 102.9, 102.8, 102.0, 100.0, 94.0, 87.2, 84.9, 84.7, 81.5, 77.7, 76.6, 76.1, 76.0, 75.4, 74.8, 74.5, 74.0, 73.6, 73.0, 72.9, 72.4, 72.2, 71.6, 71.5, 71.3, 70.8, 70.7, 70.1, 69.9, 69.6, 69.3, 69.1, 68.1, 67.5, 65.7, 65.6, 60.9, 60.8, 58.4, 58.3, 58.2, 56.2, 56.1, 54.7, 48.3, 48.1, 48.0, 46.6, 46.2, 46.0, 41.8, 41.6, 39.6, 38.1, 35.8, 33.5, 32.2, 32.1, 31.6, 31.6, 31.4, 30.1, 29.3, 27.5, 27.1, 26.3, 26.2, 24.8, 24.5, 23.1, 22.7, 22.6, 22.4, 22.3, 20.2, 17.0, 16.44, 16.37, 16.0, 15.9, 15.8, 15.1, 14.9, 13.2, 13.0, 9.6 HRMS (ESITOF) *m*/*z*: [M + H]^+^ calcd for C_88_H_145_NO_39_, 1840.9472; found, 1840.9523.

### **6** (27 mg, 97%)

^1^H NMR (500 MHz, CD_3_OD) (characteristic protons): δ 9.48 (s, 1H), 5.33 (d, *J* = 1.6 Hz, 1H), 5.31 (d, *J* = 8.2 Hz, 1H), 5.26 (t, *J* = 3.3 Hz, 1H), 5.03 (d, *J* = 1.0 Hz, 1H), 4.64 (d, *J* = 7.8 Hz, 1H), 4.58 (d, *J* = 7.6 Hz, 1H), 4.50–4.48 (m, 2H), 4.47 (m, 1H), 4.26 (dd, *J* = 3.0, 1.8 Hz, 1H), 4.14 (d, *J* = 9.3 Hz, 1H), 4.00 (dd, *J* = 3.2, 1.8 Hz, 1H), 3.91 (dd, *J* = 11.4, 5.3 Hz, 1H), 3.17 (q, 1.7 Hz, 1H), 3.15 (t, *J* = 10.9 Hz, 1H), 3.06 (dd, *J* = 9.2, 7.9 Hz, 1H), 2.82 (dd, *J* = 13.8, 3.9 Hz, 1H), 1.38 (m, 6H), 1.31 (m, 10H), 1.00 (s, 3H), 0.95 (m, 3H), 0.92 (s, 3H), 0.91 (s, 3H), 0.80 (s, 3H); ^13^C NMR (214 MHz, CD_3_OD): δ 209.5, 176.5, 168.7, 162.1, 161.9, 161.7, 161.6, 143.6, 121.8, 118.8, 117.4, 116.0, 114.7, 104.60, 104.56, 103.9, 103.7, 102.8, 102.6, 102.0, 100.0, 94.0, 92.3, 87.3, 86.4, 84.8, 84.2, 81.5, 77.7, 77.6, 77.3, 77.1, 76.8, 76.3, 76.1, 76.0, 75.4, 74.9, 74.5, 74.3, 74.0, 73.9, 73.6, 73.3, 73.0, 72.8, 72.6, 72.4, 72.2, 71.6, 71.3, 70.75, 70.67, 70.1, 70.0, 69.9, 69.62, 69.56, 69.3, 69.1, 68.9, 68.08, 68.05, 67.4, 65.7, 65.63, 65.58, 65.4, 60.9, 60.8, 56.2, 56.1, 56.0, 54.8, 49.2, 48.3, 48.1, 48.0, 46.6, 41.8, 41.6, 39.6, 35.8, 33.5, 32.2, 32.1, 31.6, 30.1, 29.2, 27.54, 27.48, 25.8, 25.6, 25.3, 25.0, 24.8, 24.6, 24.5, 24.1, 24.0, 23.1, 22.7, 22.6, 20.2, 17.0, 16.4, 16.4, 15.1, 14.9, 9.6; HRMS (ESITOF) *m*/*z*: [M + H-Boc]^+^ calcd for C_88_H_143_NO_39_, 1838.9315; found, 1838.9349.

### **7** (27 mg, 97%)

^1^H NMR (600 MHz, CD_3_OD) (characteristic protons): δ 9.51 (s, 1H), 7.39–7.38 (m, 4H), 7.34 (m, 1H), 5.44 (d, *J* = 1.5 Hz, 1H), 5.34 (t, *J* = 3.2 Hz, 1H), 5.24 (d, *J* = 8.3 Hz, 1H), 5.15 (s, 2H), 5.05 (d, *J* = 1.3 Hz, 1H), 4.75 (d, *J* = 7.9 Hz, 1H), 4.57 (d, *J* = 7.8 Hz, 1H), 4.54 (s, 1H), 4.52–4.47 (m, 2H), 4.46 (d, *J* = 7.6 Hz, 1H), 4.26 (t, *J* = 3.2, 1.8 Hz, 1H), 4.06–4.02 (m, 2H), 3.19 (t, *J* = 10.7 Hz, 1H), 3.15 (dd, *J* = 9.2, 8.1 Hz, 1H), 2.92 (dd, *J* = 9.4, 4.2 Hz, 1H), 2.37 (t, *J* = 7.3 Hz, 2H), 2.32 (t, *J* = 13.6 Hz, 1H), 1.41 (s, 3H), 1.26 (d, *J* = 6.2 Hz, 3H), 1.24 (d, *J* = 6.4 Hz, 3H),1.19 (s, 3H), 1.21 (s, 3H), 1.04 (s, 3H), 0.95 (s, 3H), 0.89 (s, 3H), 0.82 (s, 3H); ^13^C NMR (214 MHz, CD_3_OD): δ 209.6, 175.4, 173.6, 169.7, 136.5, 128.3, 128.0, 127.91, 127.88, 126.9, 126.7, 104.8, 103.8, 103.6, 102.8, 102.7, 101.8, 99.4, 94.0, 87.5, 84.5, 84.3, 77.3, 76.8, 76.6, 76.1, 75.5, 75.4, 75.0, 74.3, 74.1, 73.7, 73.3, 73.0, 72.4, 72.2, 71.6, 71.4, 70.8, 70.7, 70.5, 70.1, 69.7, 69.2, 69.0, 68.0, 65.8, 65.7, 63.8, 60.8, 60.7, 56.0, 54.8, 48.5, 48.1, 48.0, 46.6, 41.5, 41.1, 39.7, 38.8, 38.75, 38.70, 35.8, 35.3, 33.7, 33.5, 32.1, 30.0, 29.1, 29.0, 28.9, 28.8, 26.5, 26.1, 24.8, 23.4, 23.1, 22.9, 20.1, 17.2, 16.7, 16.5, 16.2, 16.1, 16.0, 15.3, 15.2, 9.8; HRMS (ESI-TOF) *m*/*z*: [M + H]^+^ calcd for C_92_H_144_NO_42_, 1934.9163; found, 1935.9277.

### **8** (17 mg, 71%)

^1^H NMR (600 MHz, CD_3_OD) (characteristic protons): δ 9.51 (s, 1H), 7.39–7.38 (m, 4H), 7.34 (m, 1H), 5.44 (d, *J* = 1.5 Hz, 1H), 5.34 (t, *J* = 3.2 Hz, 1H), 5.24 (d, *J* = 8.3 Hz, 1H), 5.15 (s, 2H), 5.05 (d, *J* = 1.3 Hz, 1H), 4.75 (d, *J* = 7.9 Hz, 1H), 4.57 (d, *J* = 7.8 Hz, 1H), 4.54 (s, 1H), 4.52–4.47 (m, 2H), 4.46 (d, *J* = 7.6 Hz, 1H), 4.26 (t, *J* = 3.2, 1.8 Hz, 1H), 4.06–4.02 (m, 2H), 3.19 (t, *J* = 10.7 Hz, 1H), 3.15 (dd, *J* = 9.2, 8.1 Hz, 1H), 2.92 (dd, *J* = 9.4, 4.2 Hz, 1H), 2.37 (t, *J* = 7.3 Hz, 2H), 2.32 (t, *J* = 13.6 Hz, 1H), 1.41 (s, 3H), 1.26 (d, *J* = 6.2 Hz, 3H), 1.24 (d, *J* = 6.4 Hz, 3H),1.19 (s, 3H), 1.21 (s, 3H), 1.04 (s, 3H), 0.95 (s, 3H), 0.89 (s, 3H), 0.82 (s, 3H); ^13^C NMR (214 MHz, DMSO-D_6_): δ 177.8, 173.8, 168.2, 136.6, 128.9, 128.6, 128.5, 128.2, 127.0, 104.5, 103.4, 101.3, 82.9, 76.9, 73.9, 72.4, 72.2, 70.6, 70.1, 69.5, 69.1, 65.9, 63.3, 60.6, 60.5, 54.6, 48.9, 48.3, 48.2, 48.1, 41.5, 41.4, 38.6, 35.8, 33.9, 33.1, 30.5, 29.7, 29.53, 29.45, 29.40, 29.2, 29.0, 28.7, 26.8, 26.6, 24.4, 22.5, 18.3, 17.9, 16.9, 16.5, 15.8, 10.6; HRMS (ESI-TOF) *m*/*z*: [M + H]^+^ calcd for C_96_H_152_NO_42_, 1990.9789; found, 1990.8888.

### **9** (15 mg, 63%)

^1^H NMR (600 MHz, CD_3_OD) (characteristic protons): δ 9.51 (s, 1H), 7.39–7.38 (m, 4H), 7.34 (m, 1H), 5.44 (d, *J* = 1.5 Hz, 1H), 5.34 (t, *J* = 3.2 Hz, 1H), 5.24 (d, *J* = 8.3 Hz, 1H), 5.15 (s, 2H), 5.05 (d, *J* = 1.3 Hz, 1H), 4.75 (d, *J* = 7.9 Hz, 1H), 4.57 (d, *J* = 7.8 Hz, 1H), 4.54 (s, 1H), 4.52–4.47 (m, 2H), 4.46 (d, *J* = 7.6 Hz, 1H), 4.26 (t, *J* = 3.2, 1.8 Hz, 1H), 4.06–4.02 (m, 2H), 3.19 (t, *J* = 10.7 Hz, 1H), 3.15 (dd, *J* = 9.2, 8.1 Hz, 1H), 2.92 (dd, *J* = 9.4, 4.2 Hz, 1H), 2.37 (t, *J* = 7.3 Hz, 2H), 2.32 (t, *J* = 13.6 Hz, 1H), 1.41 (s, 3H), 1.26 (d, *J* = 6.2 Hz, 3H), 1.24 (d, *J* = 6.4 Hz, 3H),1.19 (s, 3H), 1.21 (s, 3H), 1.04 (s, 3H), 0.95 (s, 3H), 0.89 (s, 3H), 0.82 (s, 3H); ^13^C NMR (214 MHz, CD_3_OD): δ 209.5, 173.9, 169.8, 161.9, 161.7, 143.6, 136.4, 128.1, 127.82, 127.78, 121.5, 117.4, 104.7, 103.8, 103.6, 102.8, 102.7, 101.9, 99.3, 94.0, 87.4, 84.6, 84.5, 82.1, 77.3, 76.9, 76.5, 76.0, 75.44, 75.39, 75.1, 74.3, 74.1, 73.6, 73.3, 73.0, 72.4, 72.3, 71.6, 71.52, 71.45, 70.8, 70.7, 70.5, 70.1, 70.0, 69.6, 69.2, 69.1, 68.0, 65.7, 60.8, 60.6, 58.4, 58.3, 58.2, 56.2, 56.1, 56.0, 54.8, 48.5, 46.9, 46.6, 41.5, 41.0, 39.7, 38.7, 38.0, 35.7, 35.2, 33.7, 32.7, 32.0, 30.5, 29.9, 29.6, 29.5, 29.46, 29.44, 29.3, 29.2, 29.0, 28.9, 28.7, 26.5, 25.9, 24.7, 23.3, 23.1, 20.6, 20.0, 17.0, 16.4, 16.0, 15.9, 15.8, 15.1, 9.6; HRMS (ESI-TOF) *m*/*z*: [M + H]^+^ calcd for C_98_H_156_NO_42_, 2019.0102; found, 2020.0099.

### **10** (12 mg, 53%)

^1^H NMR (600 MHz, CD_3_OD) (characteristic protons): δ 9.51 (s, 1H), 7.39–7.38 (m, 4H), 7.34 (m, 1H), 5.44 (d, *J* = 1.5 Hz, 1H), 5.34 (t, *J* = 3.2 Hz, 1H), 5.24 (d, *J* = 8.3 Hz, 1H), 5.15 (s, 2H), 5.05 (d, *J* = 1.3 Hz, 1H), 4.75 (d, *J* = 7.9 Hz, 1H), 4.57 (d, *J* = 7.8 Hz, 1H), 4.54 (s, 1H), 4.52–4.47 (m, 2H), 4.46 (d, *J* = 7.6 Hz, 1H), 4.26 (t, *J* = 3.2, 1.8 Hz, 1H), 4.06–4.02 (m, 2H), 3.19 (t, *J* = 10.7 Hz, 1H), 3.15 (dd, *J* = 9.2, 8.1 Hz, 1H), 2.92 (dd, *J* = 9.4, 4.2 Hz, 1H), 2.37 (t, *J* = 7.3 Hz, 2H), 2.32 (t, *J* = 13.6 Hz, 1H), 1.41 (s, 3H), 1.26 (d, *J* = 6.2 Hz, 3H), 1.24 (d, *J* = 6.4 Hz, 3H),1.19 (s, 3H), 1.21 (s, 3H), 1.04 (s, 3H), 0.95 (s, 3H), 0.89 (s, 3H), 0.82 (s, 3H); ^13^C NMR (214 MHz, CD_3_OD): δ 209.6, 175.6, 173.5, 169.9, 136.3, 128.2, 127.81, 127.76, 121.6, 104.7, 103.8, 103.6, 102.7, 101.9, 99.3, 94.0, 87.4, 84.6, 84.4, 82.1, 77.3, 76.9, 76.5, 76.0, 75.4, 75.1, 74.3, 74.1, 73.0, 72.4, 71.5, 70.8, 70.7, 70.5, 70.1, 70.0, 69.6, 69.2, 68.0, 67.4, 65.8, 65.7, 60.8, 60.6, 58.3, 54.8, 48.5, 48.2, 48.1, 48.0, 41.5, 41.0, 39.7, 38.2, 35.7, 35.2, 33.2, 31.9, 29.9, 28.2, 25.9, 23.3, 23.1, 21.7, 17.0, 16.4, 15.9, 15.8, 15.1, 15.0, 9.6; HRMS (ESI-TOF) *m*/*z*: [M + H]^+^ calcd for C_88_H_135_NO_42_, 1877.85; found, 1878.8777.

### 11

Benzyl 5-amino-pentanoate·HCl (100 mg, 0.41 mmol) was added to the mixture of Boc-NH-C4-acid (89 mg, 0.41 mmol), *N*-methylmorpholine (NMM) (226.0 μL, 2.05 mmol), hydroxybenzotriazole (HOBt) (188.0 mg, 1.23 mmol), and 1-ethyl-3-(3-(dimethylamino)­propyl) carbodiimide hydrochloride (EDC·HCl) (243 mg, 1.23 mmol) in dichloromethane (1.5 mL) at room temperature. The reaction mixture was diluted with dichloromethane (5 mL) and washed with saturated NH_4_Cl (4 × 10 mL) and dried over Na_2_SO_4_. Removal of solvent from the dried extracts on a rotary evaporator afforded the intermediate, which was further treated with 30% (v/v) trifluoroacetic acid (TFA) in DCM (1 mL) at room temperature for 45 min to remove the Boc protecting group. After the reaction mixture was treated with Na_2_CO_3_ three times, the organic layer was concentrated through rotor vap. and side chain **15** was obtained as white solid (13 mg, 85%); R_*f*_ (DCM/MeOH) 0.22. The side chain was then incorporated into MS II to provide **11** (25 mg, 83%). ^1^H NMR (600 MHz, CD_3_OD) (characteristic protons): δ 9.50 (s, 1H), 7.37–7.34 (m, 4H), 7.32 (m, 1H), 5.41 (d, *J* = 1.5 Hz, 1H), 5.31 (t, *J* = 3.2 Hz, 1H), 5.21 (d, *J* = 8.3 Hz, 1H), 5.12 (s, 2H), 5.02 (d, *J* = 1.3 Hz, 1H), 4.71 (d, *J* = 7.9 Hz, 1H), 4.54 (d, *J* = 7.8 Hz, 1H), 4.51 (s, 1H), 4.47–4.45 (m, 2H), 4.43 (d, *J* = 7.6 Hz, 1H), 4.22 (t, *J* = 3.2, 1.8 Hz, 1H), 4.03–4.00 (m, 2H), 3.20–3.14 (m, 4H), 3.12 (dd, *J* = 9.2, 8.1 Hz, 1H), 2.89 (dd, *J* = 9.4, 4.2 Hz, 1H), 2.41 (t, *J* = 7.3 Hz, 2H), 2.28 (t, *J* = 13.6 Hz, 1H), 2.19 (t, *J* = 7.3 Hz), 1.41 (s, 3H), 1.31 (d, *J* = 6.2 Hz, 4H), 1.23 (t, *J* = 6.0 Hz, 7H),1.17 (s, 4H), 1.00 (s, 3H), 0.93 (s, 3H), 0.87 (s, 3H), 0.79 (s, 3H); ^13^C NMR (214 MHz, CD_3_OD): δ 209.6, 175.6, 174.4, 173.5, 169.8, 161.9, 161.7, 143.5, 136.3, 128.2, 127.8, 121.6, 117.4, 116, 104.7, 103.8, 103.6, 102.7, 102.7, 101.9, 99.3, 94, 87.4, 84.6, 84.4, 82.1, 77.3, 76.9, 76.4, 76.0, 75.5, 75.4, 75.1, 74.3, 74.0, 73.6, 73.3, 73.0, 72.4, 72.3, 71.6, 71.5, 71.5, 70.8, 70.7, 70.5, 70.1, 70.0, 69.6, 69.2, 69.1, 68.0, 67.4, 65.8, 65.7, 60.8, 60.6, 56.1, 54.8, 47.8, 47.7, 47.6, 47.5, 47.4, 47.3, 41.5, 41.1, 39.7, 38.5, 38.4, 38.3, 37.9, 35.7, 35.2, 35.1, 33.2, 32.7, 31.9, 30.5, 29.9, 28.4, 28.3, 25.9, 24.5, 23.3, 23.1, 22.7, 22, 20.0, 17, 16.5, 16.4, 15.9, 15.1, 15.0, 9.6; HRMS (ESI-TOF) *m*/*z*: [M + H]^+^ calcd for C_93_H_145_NO_43_, 1977.9221; found, 1977.9209.

### 12

Dimeric side chain **15** (50 mg, 0.16 mmol) was added to the mixture of Boc-NH-C4-acid (35.4 mg, 0.16 mmol), NMM (90 μL, 0.82 mmol), HOBt (77 mg, 0.5 mmol), and EDC·HCl (99.0 mg, 0.5 mmol) in DCM (0.5 mL) at room temperature, and the reaction mixture was stirred for 16 h. To get rid of the unreacted monomer by making it carboxylate, the reaction mixture was diluted in DCM (10 mL) and washed with saturated NaHCO_3_ (10 mL × 3). The organic layer was concentrated through rotor vap. and directly treated with 30% (v/v) TFA in DCM (1 mL) at room temperature for 30 min to remove the Boc protecting group. The mixture was directly purified with reverse-phase high-performance liquid chromatography (RP HPLC) by using a Prep C18, 250 × 10 mm, 5 μm column, and H_2_O/acetonitrile (MeCN) gradients (90–40% H_2_O over 14.5 min with a 25 mL/min flow rate). The product fraction was concentrated on a rotary evaporator at room temperature to remove MeCN, and the remaining water was then removed on a lyophilizer to provide trimeric side chain **16** as a white solid (6 mg, 100%); R_*f*_ (DCM/MeOH) 0.12. ^1^H NMR (500 MHz, CD_3_OD) (characteristic protons): δ 7.37–7.28 (m, 5H), 5.11 (s, 2H), 3.18 (q, *J* = 6.84, 4H), 2.93 (t, *J* = 6.62 Hz, 2H), 2.40 (t, *J* = 7.31 Hz, 2H), 2.24 (t, *J* = 6.67 Hz, 2H), 2.19 (t, *J* = 7.29 Hz, 2H), 1.71–1.56 (m, 8H), 1.55–1.46 (m, 4H). The side chain was then incorporated into MS II to provide **12** (16 mg, 64%). ^1^H NMR (600 MHz, CD_3_OD) (characteristic protons): δ 9.47 (s, 1H), 7.36–7.34 (m, 4H), 7.32 (m, 1H), 5.41 (d, *J* = 1.5 Hz, 1H), 5.31 (t, *J* = 3.2 Hz, 1H), 5.21 (d, *J* = 8.3 Hz, 1H), 5.12 (s, 2H), 5.02 (d, *J* = 1.3 Hz, 1H), 4.72 (d, *J* = 7.9 Hz, 1H), 4.54 (d, *J* = 7.8 Hz, 1H), 4.50 (s, 1H), 4.47–4.45 (m, 2H), 4.44 (d, *J* = 7.6 Hz, 1H), 4.23 (t, *J* = 3.2, 1.8 Hz, 1H), 4.04–4.00 (m, 2H), 3.20–3.16 (m, 6H), 3.12 (dd, *J* = 9.2, 8.1 Hz, 1H), 2.90 (dd, *J* = 9.4, 4.2 Hz, 1H), 2.41 (t, *J* = 7.3 Hz, 2H), 2.28 (t, *J* = 13.6 Hz, 1H), 2.23–2.17 (m, 4H), 1.23 (d, *J* = 6.2 Hz, 3H), 1.22 (d, *J* = 6.4 Hz, 3H),1.17 (s, 3H), 1.01 (s, 3H), 0.93 (s, 3H), 0.87 (s, 3H), 0.79 (s, 3H); ^13^C NMR (214 MHz, CD_3_OD): δ 209.6, 175.6, 174.4, 174.4, 173.6, 169.9, 169.8, 161.8, 161.6, 143.5, 136.3, 128.2, 127.8, 121.6, 116.0, 104.7, 103.8, 103.6, 102.7, 102.7, 101.9, 99.3, 94.0, 87.4, 84.6, 84.4, 82.1, 77.3, 76.9, 76.4, 76.0, 75.5, 75.4, 75.1, 74.3, 74.0, 73.6, 73.4, 73.0, 72.4, 72.3, 71.7, 71.5, 71.5, 70.8, 70.7, 70.5, 70.1, 70, 69.6, 69.2, 69.1, 68, 67.4, 65.8, 65.7, 60.8, 60.7, 58.3, 56.1, 54.8, 48.5, 46.6, 41.5, 41.1, 39.7, 38.5, 38.5, 38.3, 37.9, 35.7, 35.2, 35.2, 35.1, 33.2, 32.7, 32.0, 30.5, 29.9, 28.5, 28.4, 28.3, 25.9, 24.5, 23.3, 23.1, 22.9, 22.7, 21.9, 20.0, 17.0, 16.5, 16.4, 16.0, 15.1, 15.0, 9.6; HRMS (ESI-TOF) *m*/*z*: [M + H]^+^ calcd for C_98_H_154_N_3_O_44_, 2076.9905; found, 2076.9915.

### **V1H** (10 mg, 83%)

^1^H NMR (850 MHz, CD_3_OD) (characteristic protons): δ 5.27 (d, *J* = 1.36 Hz, 1H), 5.22 (d, *J* = 8.2 Hz, 1H), 5.15 (t, *J* = 3.6 Hz, 1H), 4.59 (d, *J* = 1.5 Hz, 1H), 4.54 (d, *J* = 7.9 Hz, 1H), 4.52 (dd, *J* = 7.7, 3.0 Hz, 2H), 4.48 (s, 1H), 4.45 (d, *J* = 7.6 Hz, 1H), 4.38 (d, *J* = 7.6 Hz, 1H), 4.14 (dd, *J* = 3.0, 1.7 Hz, 1H), 3.96 (dd, *J* = 3.1, 1.9 Hz, 1H), 3.92 (m, 1H), 3.84–3.79 (m, 3H), 3.04 (t, *J* = 10.8 Hz, 1H), 2.98 (dd, *J* = 9.1, 7.9 Hz, 1H), 2.70 (dd, *J* = 13.7, 4.0 Hz, 1H), 1.08 (s, 3H), 0.88 (s, 3H), 0.69 (s, 3H), 0.65 (s, 3H); ^13^C NMR (850 MHz, CD_3_OD): δ 176.6, 169.8, 143.5, 129.5, 129.4, 122.1, 104.6, 104.0, 103.7, 102.8, 102.8, 102.0, 100.0, 94.0, 86.9, 85.0, 83.2, 81.4, 77.8, 77.0, 76.1, 75.9, 75.6, 75.4, 75.0, 74.5, 73.4, 73.1, 72.6, 72.4, 72.3, 71.5, 71.3, 70.8, 70.7, 70.6, 70.2, 70.1, 69.6, 69.3, 68.7, 68.0, 67.4, 65.8, 65.6, 63.6, 61.1, 60.7, 58.4, 58.3, 58.2, 56.2, 56.1, 46.7, 46.0, 42.8, 41.9, 41.7, 39.3, 38.7, 38.4, 36.3, 33.5, 32.4, 32.1, 31.7, 31.7, 31.6, 30.1, 29.5, 29.4, 29.4, 29.4, 29.2, 29.1, 29.1, 29.0, 28.9, 28.9, 28.8, 27.7, 26.7, 26.5, 25.5, 25.1, 24.8, 23.2, 22.7, 22.6, 22.4, 22.3, 17.9, 17.0, 16.4, 16.4, 15.9, 15.8, 15.2, 15.1, 13.1, 13.0, 12.0; HRMS (ESI-TOF) *m*/*z*: [M + H]^+^ calcd for C_88_H_148_NO_39_, 1842.9628; found, 1842.9617.

### **V2H** (12.1 mg, 37%)

^1^H NMR (850 MHz, CD_3_OD) (characteristic protons): δ 7.89 (t, *J* = 5.7 Hz, 1H), 7.38 (s, 2H), 7.37 (d, *J* = 1.0 Hz, 2H), 7.31–7.29 (m, 1H), 5.42 (d, *J* = 1.5 Hz, 1H), 5.33 (t, *J* = 3.6 Hz, 1H), 5.25 (d, *J* = 8.2 Hz, 1H), 5.14 (s, 2H), 5.03 (d, *J* = 1.6 Hz, 1H), 4.74 (d, *J* = 8.0 Hz, 1H), 4.63 (d, *J* = 7.5 Hz, 1H), 4.62 (d, *J* = 7.8 Hz, 1H), 4.58 (d, *J* = 7.6 Hz, 1H), 4.53–4.51 (m, 2H), 4.35 (dd, *J* = 3.1, 1.9 Hz, 1H), 4.07 (dd, *J* = 3.2, 1.9 Hz, 1H), 4.02 (m, 1H), 2.83 (dd, *J* = 14.0, 4.1 Hz, 1H), 2.39 (t, *J* = 7.3 Hz, 2H), 2.31 (t, *J* = 13.7 Hz, 1H), 1.42 (s, 3H), 1.26 (d, *J* = 6.2 Hz, 4H), 1.23 (d, *J* = 6.4 Hz, 4H), 1.01 (s, 3H), 0.95 (s, 3H), 0.88 (s, 3H), 0.80 (s, 3H), 0.78 (s, 3H); ^13^C NMR (850 MHz, CD_3_OD): δ 173.8, 136.4, 128.2, 127.9, 127.8, 104.6, 103.7, 103.7, 102.8, 102.7, 102.0, 94.0, 87.0, 85.0, 83.7, 81.9, 77.3, 76.9, 76.1, 76.0, 75.6, 75.5, 74.3, 73.9, 73.4, 73.1, 72.4, 72.3, 71.4, 70.8, 70.7, 70.1, 69.6, 69.3, 68.7, 68.0, 67.5, 65.8, 65.7, 61.1, 60.6, 58.3, 48.6, 48.1, 46.7, 42.7, 41.5, 41.0, 39.4, 38.8, 36.3, 35.2, 33.7, 32.9, 32.0, 29.9, 29.4, 29.2, 29.1, 29.0, 28.9, 28.7, 26.5, 26.0, 24.7, 23.3, 23.2, 17.1, 16.4, 15.8, 15.3, 15.1, 12.0; HRMS (ESI-TOF) *m*/*z*: [M + H]^+^ calcd for C_94_H_150_NO_42_, 1964.9632; found, 1964.8889.

### Immunological Studies

All in vivo studies were performed in accordance with proper guidelines and approved by the University of Alabama at Birmingham Institutional Animal Care and Use Committee (Animal Project Number: IACUC-22582).

### Antigens

The chicken egg albumin for in vivo use (Vac-pova) was purchased from InvivoGen.

### Mice and Immunization

BALB/c mice used in this study were purchased from Charles River (Hartford, CT) and maintained within an environmentally controlled, pathogen-free animal facility at the University of Alabama at Birmingham (UAB). To assess the adjuvant activity of the MS saponin-based immune adjuvants, groups of female mice (8–10 weeks of age; 5 mice per group) were immunized by the subcutaneous (s.c.) route with OVA (20 μg) alone or with antigen plus proper adjuvant on days 0, 14, and 28. The injection solution for each group (1.2 mL total volume) was prepared by diluting the antigen solution (120 μL, 1 mg/mL), either alone or combined with an adjuvant solution (600 μL, 1 mg/mL), with saline to a final volume of 1.2 mL. Prior to each immunization and at 2 weeks post last immunization, mice were weighed, and blood samples were collected from the submandibular vein. The serum was obtained after centrifugation and stored at −20 °C until assayed. All studies were performed according to the National Institutes of Health guidelines, and protocols were approved by the UAB Institutional Animal Care and Use Committee.

### Evaluation of Antibody Responses

The levels of specific serum IgG and IgG subclasses against OVA in each group were determined by an enzyme-linked immunosorbent assay (ELISA). Maxisorpmicrotiter plates (NUNC International, Roskilde, Denmark) were coated with OVA (0.1 μg/mL) or with optimal amounts of goat antimouse IgG1 or IgG2a (Southern Biotechnology Associates, Inc., Birmingham, AL) in phosphate-buffered saline (PBS) at 4 °C overnight. Plates were blocked with 1% bovine serum albumin (BSA) in PBS/0.05% Tween 20 (PBST) for 2 h at room temperature. Serial 2-fold dilutions of serum samples were added in duplicate to the plates. To generate standard curves, serial dilutions of a mouse immunoglobulin reference serum (Southern Biotechnology Associates, Inc.) were added to two rows of wells in each plate that had been coated with the appropriate antimouse IgG or IgG subclass reagent. After incubation (overnight at 4 °C) and washing of the plates, horseradish peroxidase-conjugated goat antimouse IgG1 or IgG2a antibody (Southern Biotechnology Associates, Inc.) was added to appropriate wells. After 4 h of incubation at room temperature, plates were washed and developed by an *o*-phenylenediamine substrate with hydrogen peroxide. After 15 min, color development was stopped by adding 1 N of H_2_SO_4_, and absorbance was recorded at 490 nm. The concentrations of antibodies were determined by interpolation on standard curves generated by using the mouse immunoglobulin reference serum and constructed by a computer program based on four-parameter logistic algorithms (Softmax/Molecular Devices Corp., Menlo Park, CA).

### Statistical Analysis

Statistical significance in antibody responses was evaluated by *t* tests (with unpaired, nonparametric, and Mann–Whitney test) using GraphPad Prism 8.0.1. Differences were considered significant at a *P* value <0.05.

## Supplementary Material



## Notes

^1^H and ^13^C NMR spectra of the new compounds **1**–**12**, **V1H**, and **V2H** (PDF)

## Abbreviations

Api: apiose
Ara: arabinose
Bn: benzyl
Boc: *tert*-butyloxycarbonyl
CD4: cluster differentiation 4
CD8: cluster differentiation 8
CTL: cytotoxic T cell
DCM: dichloromethane
EDC·HCl: 1-ethyl-3-(3-(dimethylamino)­propyl) carbodiimide
ELISA: enzyme-linked immunosorbent assay
ESI-TOF: electrospray ionization time-of-flight mass spectrometry
HBTU: *O*-(Benzotriazol-1-yl)-*N*,*N*,*N*′,*N*′-tetramethyluronium hexafluorophosphate
HOBt: hydroxybenzotriazole
IACUC: International Animal Care and Use Committee
IFN-γ: interferon-γ
IgG: immunoglobulin G
NaBH_4_: sodium borohydride
MeCN: acetonitrile
MeOH: methanol
NMM: *N*-methylmorpholine
OVA: chicken egg ovalbumin
RP HPLC: Reversed-phase High-Performance Liquid Chromatography
r.t.: room temperature
s.c: subcutaneous
SOCl_2_: thionyl chloride
TFA: trifluoroacetic acid
THF: tetrahydrofuran
Th: T helper cells
TNF-α: tumor necrosis factor-α
xyl: xylose
