# Supplementary material for: Impact of Side Chain Structure and Aglycon Carbonyl Group on the Immunostimulatory Activities of Semisynthetic Saponin Adjuvants
Source: ACS Pharmacol Transl Sci. 2026 Feb 20;9(3):706–15. doi: 10.1021/acsptsci.5c00792 (PMC12993776; doi:10.1021/acsptsci.5c00792)
Supplement: Supplementary file 1 [file pt5c00792_si_001.pdf]

Impact of side chain structure and aglycon carbonyl group on immunostimulatory activities of semisynthetic saponin adjuvants

Di Bai,[1] Liz Wang,[1] Rebekah Beyea,[2] Hyunjung Kim*[2], and Pengfei Wang*[1,2]

[1]Department of Chemistry, University of Alabama at Birmingham, 901 14th Street South, Birmingham, AL 35294, U.S.A. [2]Adjuvax LLC, 2000 9th Avenue South, Birmingham, AL35205, U.S.A.

Victoria.kim@adjuvax.net and wangp@uab.edu

Table of content	S1
Fig. S1	S2
Fig. S2	S2
^1H and ^{13}C NMR spectra of 1	S3-4
^1H and ^{13}C NMR spectra of 2	S5-6
^1H and ^{13}C NMR spectra of 3	S7-8
^1H and ^{13}C NMR spectra of 4	S9-10
^1H and ^{13}C NMR spectra of 5	S11-12
^1H and ^{13}C NMR spectra of 6	S13-14
^1H and ^{13}C NMR spectra of 7	S15-16
^1H and ^{13}C NMR spectra of 8	S17-18
^1H and ^{13}C NMR spectra of 9	S19-20
^1H and ^{13}C NMR spectra of 10	S21-22
^1H and ^{13}C NMR spectra of 11	S23-24
^1H and ^{13}C NMR spectra of 12	S25-26
^1H and ^{13}C NMR spectra of V1H	S27-28
^1H and ^{13}C NMR spectra of V2H	S29-30

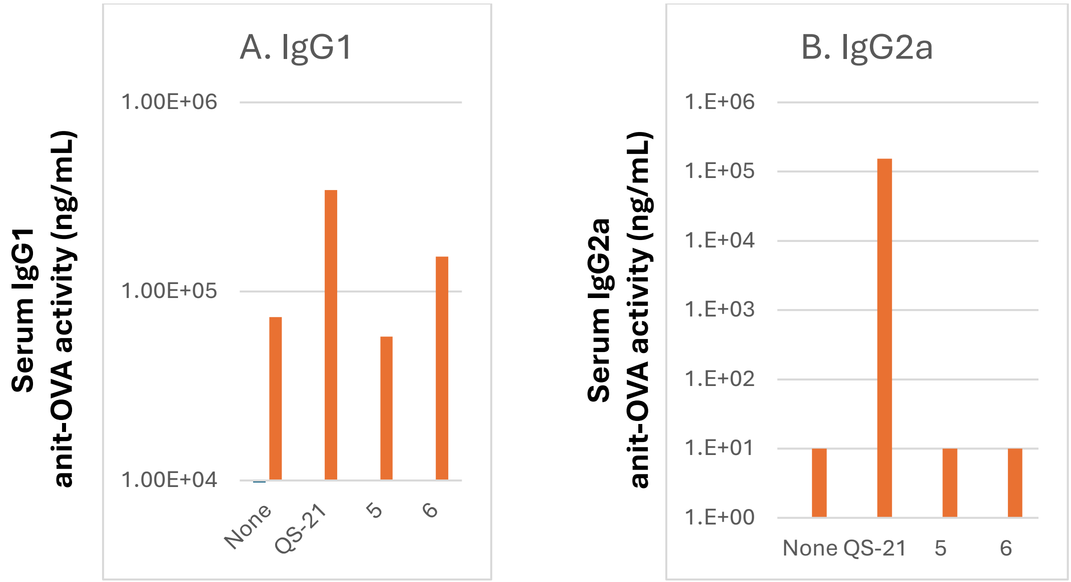

Figure S1. Serum antibody activity (A) week 6 IgG1 and (B) week 6 IgG2a. BALB/c mice (five per group) were immunized via the subcutaneous route (*s.c.*) on days 0, 14 and 28. Serum samples were collected at 2 weeks following the last immunization. The pooled serum samples of each group were analyzed by ELISA.

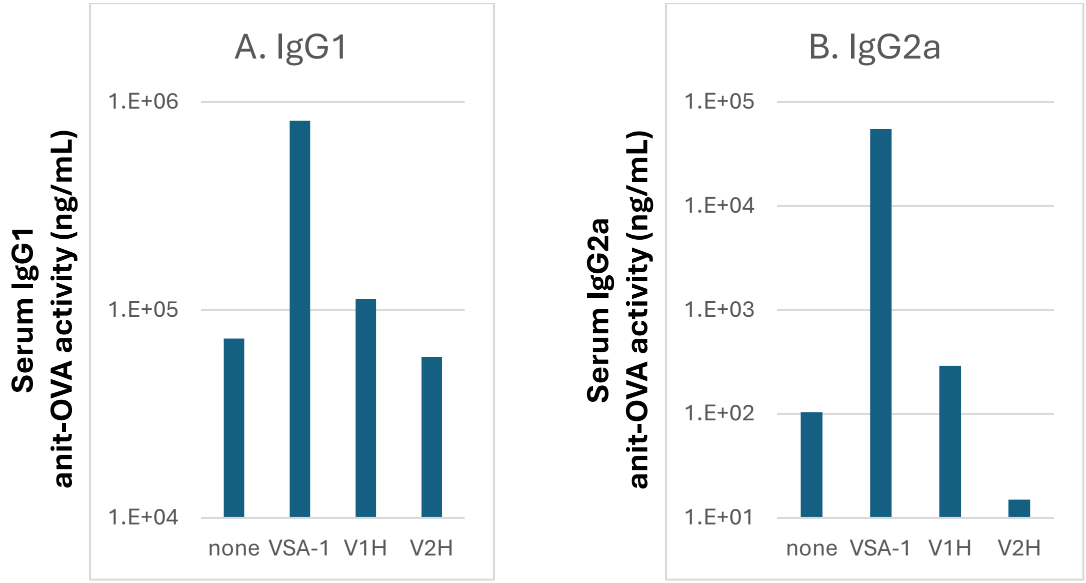

Figure S2. Serum antibody activity (A) week 6 IgG1 and (B) week 6 IgG2a. BALB/c mice (five per group) were immunized via the subcutaneous route (*s.c.*) on days 0, 14 and 28. Serum samples were collected at 2 weeks following the last immunization. The pooled serum samples of each group were analyzed by ELISA.

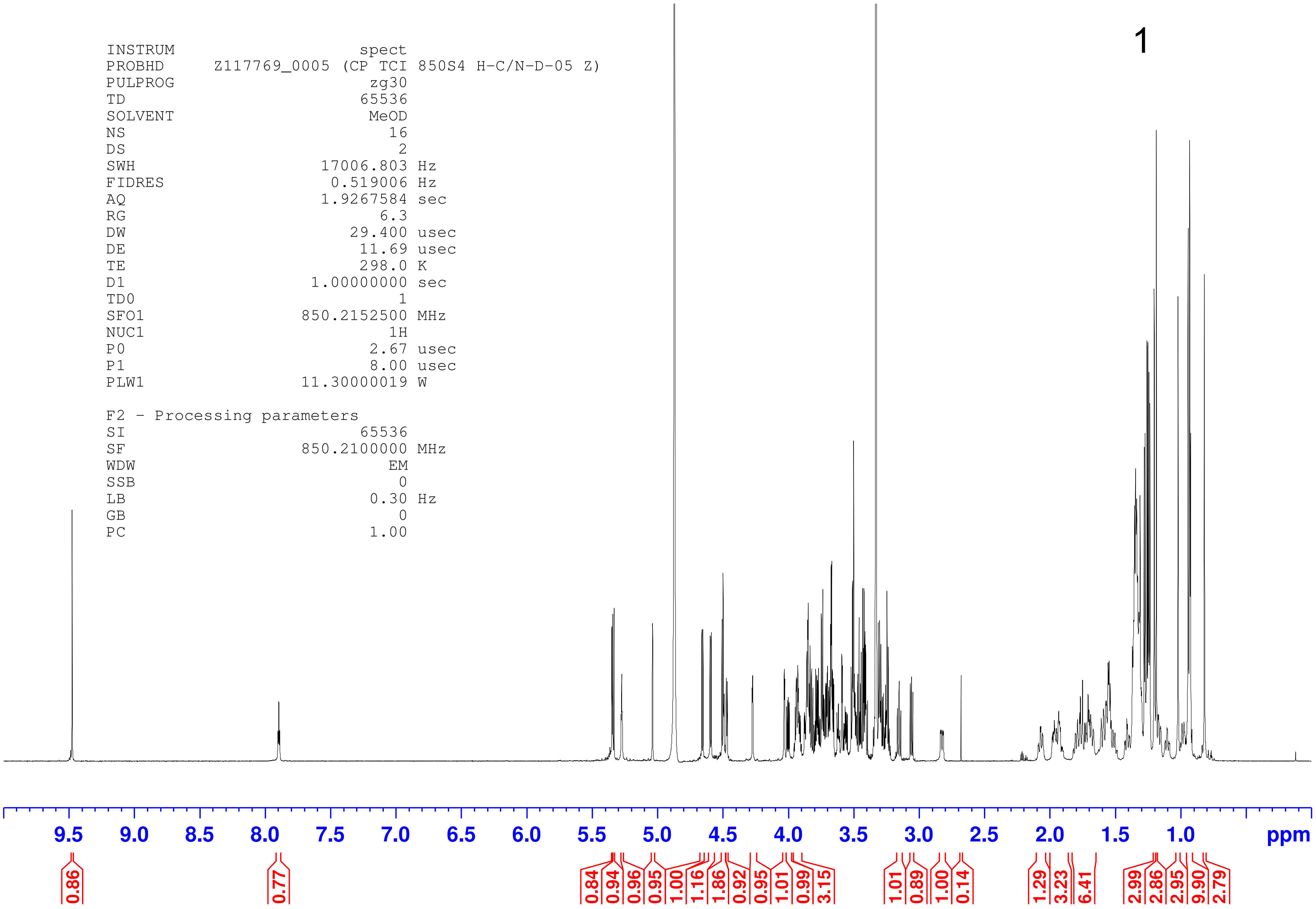
INSTRUM spect
PROBHD Z117769_0005 (CP TCI 850S4 H-C/N-D-05 Z)
PULPROG zg30
TD 65536
SOLVENT MeOD
NS 16
DS 2
SWH 17006.803 Hz
FIDRES 0.519006 Hz
AQ 1.9267584 sec
RG 6.3
DW 29.400 usec
DE 11.69 usec
TE 298.0 K
D1 1.00000000 sec
TD0 1
SFO1 850.2152500 MHz
NUC1 1H
P0 2.67 usec
P1 8.00 usec
PLW1 11.30000019 W
F2 - Processing parameters
SI 65536
SF 850.2100000 MHz
WDW EM
SSB 0
LB 0.30 Hz
GB 0
PC 1.00
9.5 9.0 8.5 8.0 7.5 7.0 6.5 6.0 5.5 5.0 4.5 4.0 3.5 3.0 2.5 2.0 1.5 1.0 ppm
0.86
0.77
0.84
0.94
0.96
0.95
1.00
1.16
1.86
0.92
0.95
1.01
0.99
3.15
1.01
0.89
1.00
0.14
1.29
3.23
6.41
2.99
2.86
2.95
9.90
2.79

1

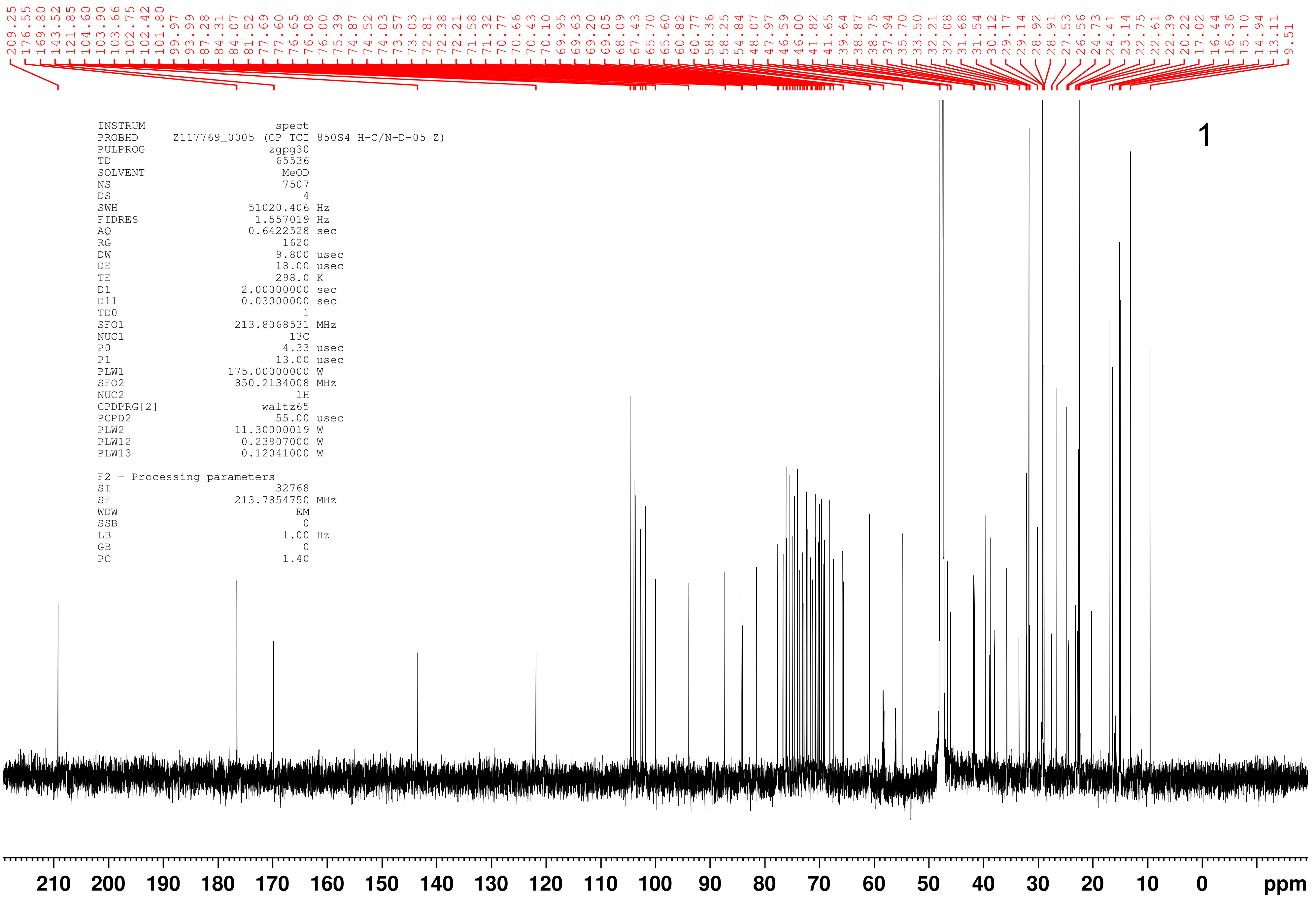
209.25
176.55
169.80
143.52
121.85
104.60
103.90
103.66
102.75
102.42
101.80
99.97
93.99
87.28
84.31
84.07
81.52
77.69
77.60
76.65
76.08
76.00
75.39
74.87
74.52
74.03
73.57
73.03
72.81
72.38
72.21
71.58
71.32
70.77
70.66
70.43
70.10
69.95
69.63
69.20
69.05
68.09
67.43
65.70
65.60
60.82
60.77
58.36
58.25
54.84
48.07
47.97
46.59
46.00
41.82
41.65
39.64
38.87
38.75
37.94
35.70
33.50
32.21
32.08
31.68
31.54
30.12
29.17
29.14
28.92
28.91
27.53
26.56
24.73
24.41
23.14
22.75
22.61
22.39
20.22
17.02
16.44
16.36
15.10
14.94
13.11
9.51
1
INSTRUM spect
PROBHD Z117769_0005 (CP TCI 850S4 H-C/N-D-05 Z)
PULPROG zgpg30
TD 65536
SOLVENT MeOD
NS 7507
DS 4
SWH 51020.406 Hz
FIDRES 1.557019 Hz
AQ 0.6422528 sec
RG 1620
DW 9.800 usec
DE 18.00 usec
TE 298.0 K
D1 2.00000000 sec
D11 0.03000000 sec
TD0 1
SFO1 213.8068531 MHz
NUC1 13C
P0 4.33 usec
P1 13.00 usec
PLW1 175.00000000 W
SFO2 850.2134008 MHz
NUC2 1H
CPDPRG[2] waltz65
PCPD2 55.00 usec
PLW2 11.30000019 W
PLW12 0.23907000 W
PLW13 0.12041000 W
F2 - Processing parameters
SI 32768
SF 213.7854750 MHz
WDW EM
SSB 0
LB 1.00 Hz
GB 0
PC 1.40
210 200 190 180 170 160 150 140 130 120 110 100 90 80 70 60 50 40 30 20 10 0 ppm

2

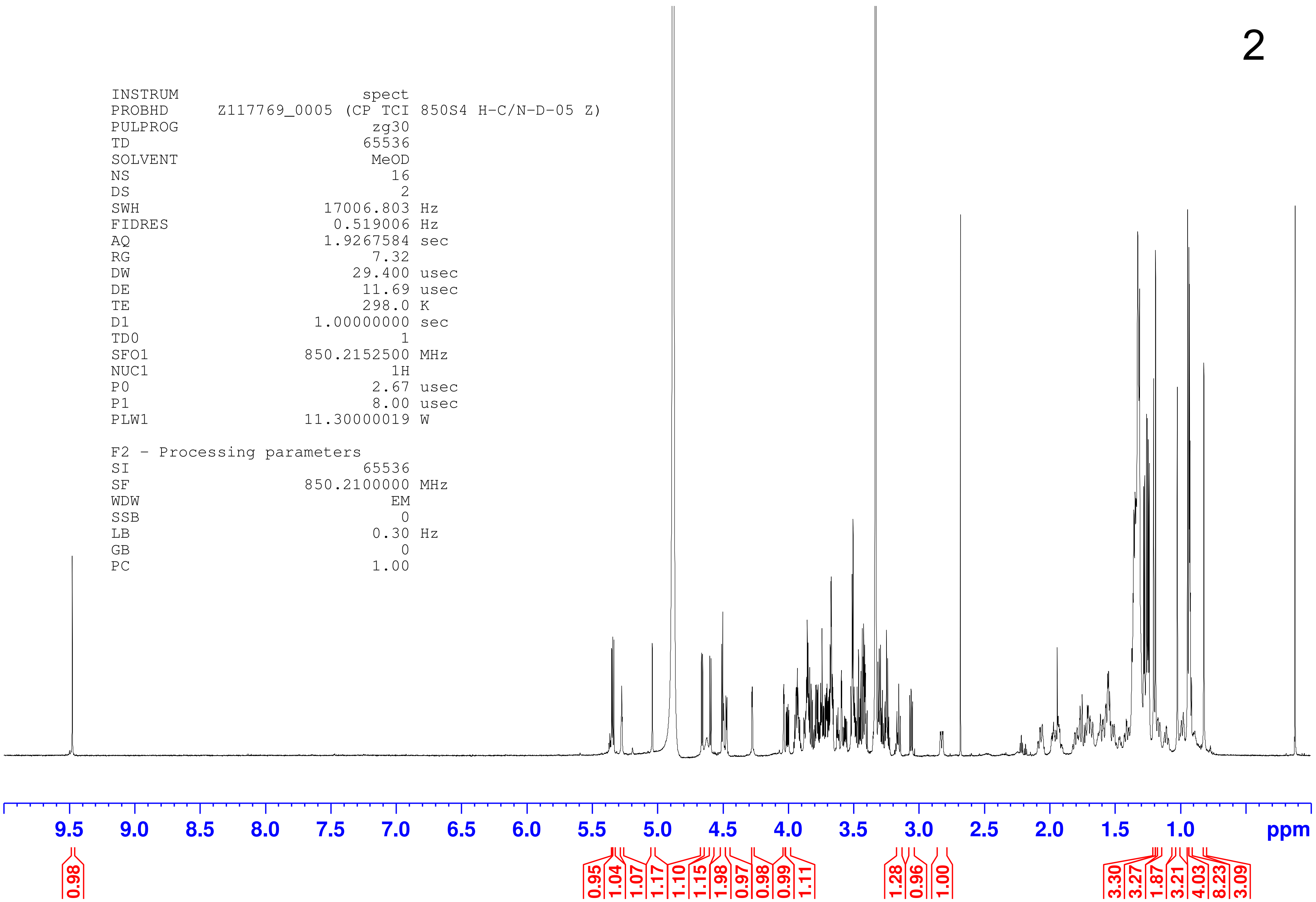
INSTRUM spect
PROBHD Z117769_0005 (CP TCI 850S4 H-C/N-D-05 Z)
PULPROG zg30
TD 65536
SOLVENT MeOD
NS 16
DS 2
SWH 17006.803 Hz
FIDRES 0.519006 Hz
AQ 1.9267584 sec
RG 7.32
DW 29.400 usec
DE 11.69 usec
TE 298.0 K
D1 1.00000000 sec
TD0 1
SFO1 850.2152500 MHz
NUC1 1H
P0 2.67 usec
P1 8.00 usec
PLW1 11.30000019 W
F2 - Processing parameters
SI 65536
SF 850.2100000 MHz
WDW EM
SSB 0
LB 0.30 Hz
GB 0
PC 1.00
9.5 9.0 8.5 8.0 7.5 7.0 6.5 6.0 5.5 5.0 4.5 4.0 3.5 3.0 2.5 2.0 1.5 1.0 ppm
0.98
0.95 1.04 1.07 1.17 1.10 1.15 1.98 0.97 0.98 0.99 1.11
1.28 0.96 1.00
3.30 3.27 1.87 3.21 4.03 8.23 3.09

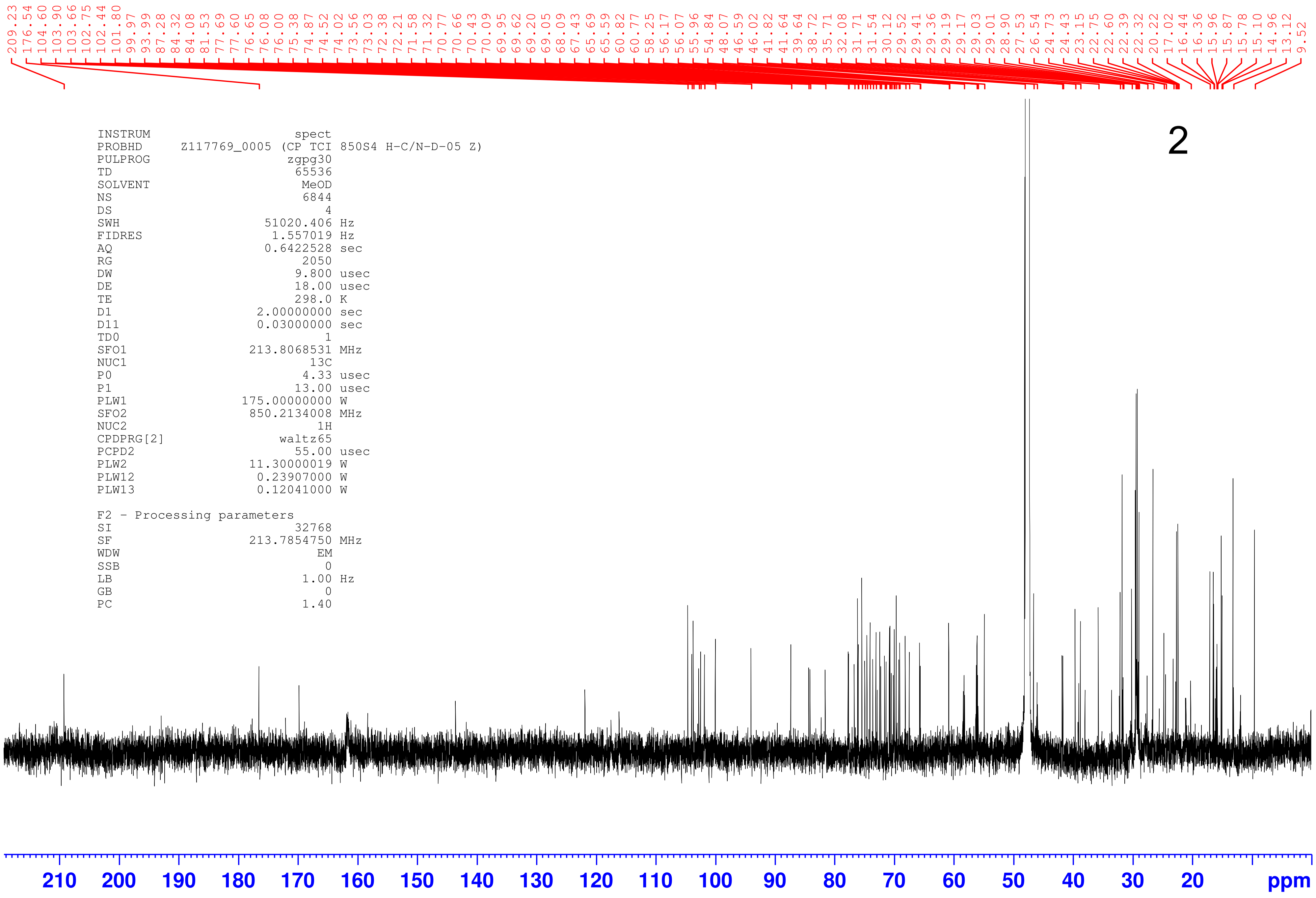
209.23
176.54
104.60
103.90
103.66
102.75
102.44
101.80
99.97
93.99
87.28
84.32
84.08
81.53
77.69
77.60
76.65
76.08
76.00
75.38
74.87
74.52
74.02
73.56
73.03
72.38
72.21
71.58
71.32
70.77
70.66
70.43
70.09
69.95
69.62
69.20
69.05
68.09
67.43
65.69
65.59
60.82
60.77
58.25
56.17
56.07
55.96
54.84
48.07
46.59
46.02
41.82
41.64
39.64
38.72
35.71
32.08
31.71
31.54
30.12
29.52
29.41
29.36
29.19
29.17
29.03
29.01
28.90
27.53
26.54
24.73
24.43
23.15
22.75
22.60
22.39
22.32
20.22
17.02
16.44
16.36
15.96
15.87
15.78
15.10
14.96
13.12
9.52
INSTRUM spect
PROBHD Z117769_0005 (CP TCI 850S4 H-C/N-D-05 Z)
PULPROG zgpg30
TD 65536
SOLVENT MeOD
NS 6844
DS 4
SWH 51020.406 Hz
FIDRES 1.557019 Hz
AQ 0.6422528 sec
RG 2050
DW 9.800 usec
DE 18.00 usec
TE 298.0 K
D1 2.00000000 sec
D11 0.03000000 sec
TD0 1
SFO1 213.8068531 MHz
NUC1 13C
P0 4.33 usec
P1 13.00 usec
PLW1 175.00000000 W
SFO2 850.2134008 MHz
NUC2 1H
CPDPRG[2] waltz65
PCPD2 55.00 usec
PLW2 11.30000019 W
PLW12 0.23907000 W
PLW13 0.12041000 W
F2 - Processing parameters
SI 32768
SF 213.7854750 MHz
WDW EM
SSB 0
LB 1.00 Hz
GB 0
PC 1.40
210
200
190
180
170
160
150
140
130
120
110
100
90
80
70
60
50
40
30
20
ppm

2

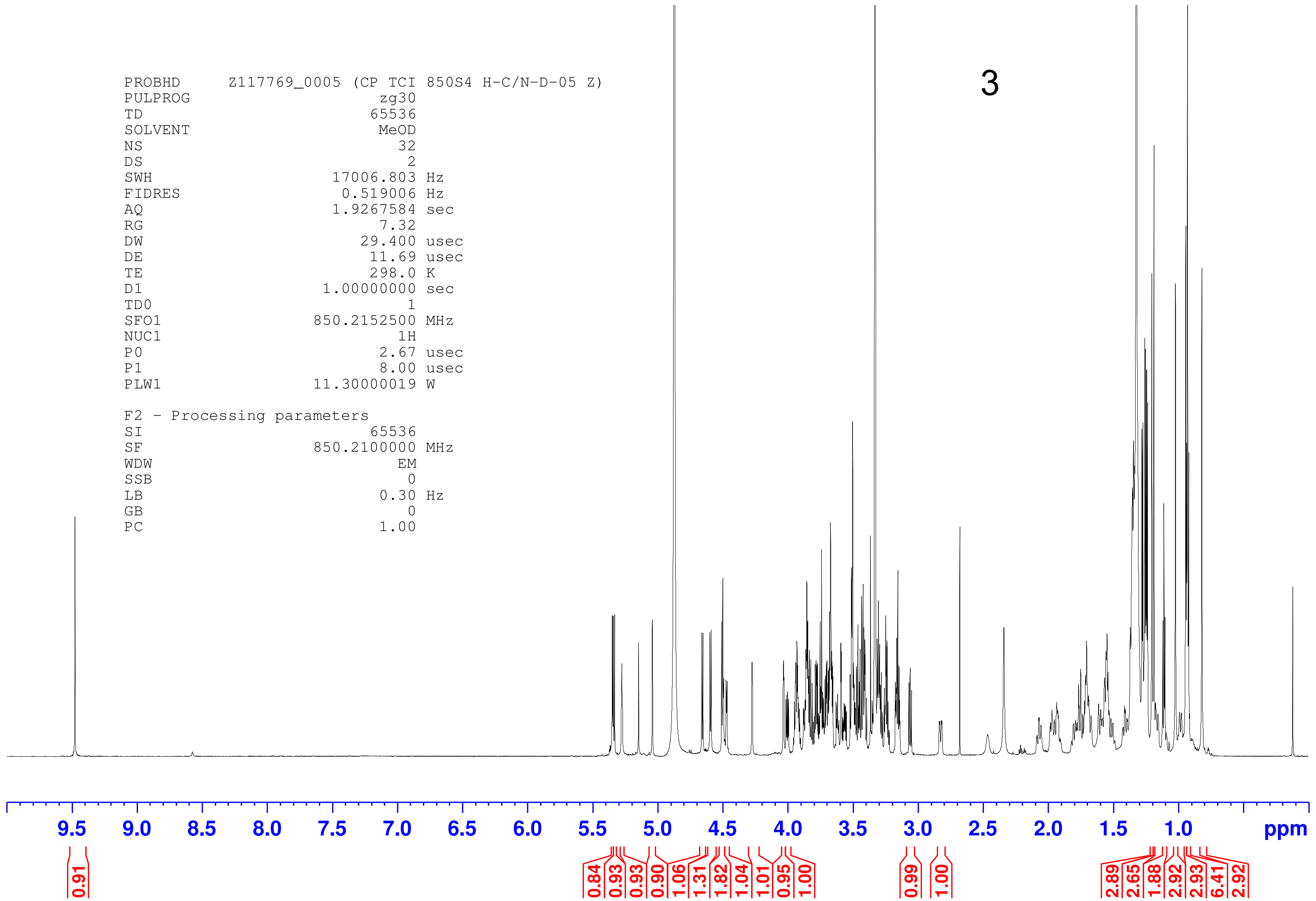
PROBHD Z117769_0005 (CP TCI 850S4 H-C/N-D-05 Z)
PULPROG zg30
TD 65536
SOLVENT MeOD
NS 32
DS 2
SWH 17006.803 Hz
FIDRES 0.519006 Hz
AQ 1.9267584 sec
RG 7.32
DW 29.400 usec
DE 11.69 usec
TE 298.0 K
D1 1.00000000 sec
TD0 1
SFO1 850.2152500 MHz
NUC1 1H
P0 2.67 usec
P1 8.00 usec
PLW1 11.30000019 W
F2 - Processing parameters
SI 65536
SF 850.2100000 MHz
WDW EM
SSB 0
LB 0.30 Hz
GB 0
PC 1.00
9.5 9.0 8.5 8.0 7.5 7.0 6.5 6.0 5.5 5.0 4.5 4.0 3.5 3.0 2.5 2.0 1.5 1.0 ppm
0.91
0.84
0.93
0.93
0.90
1.06
1.31
1.82
1.04
1.01
0.95
1.00
0.99
1.00
2.89
2.65
1.88
2.92
2.93
6.41
2.92
3

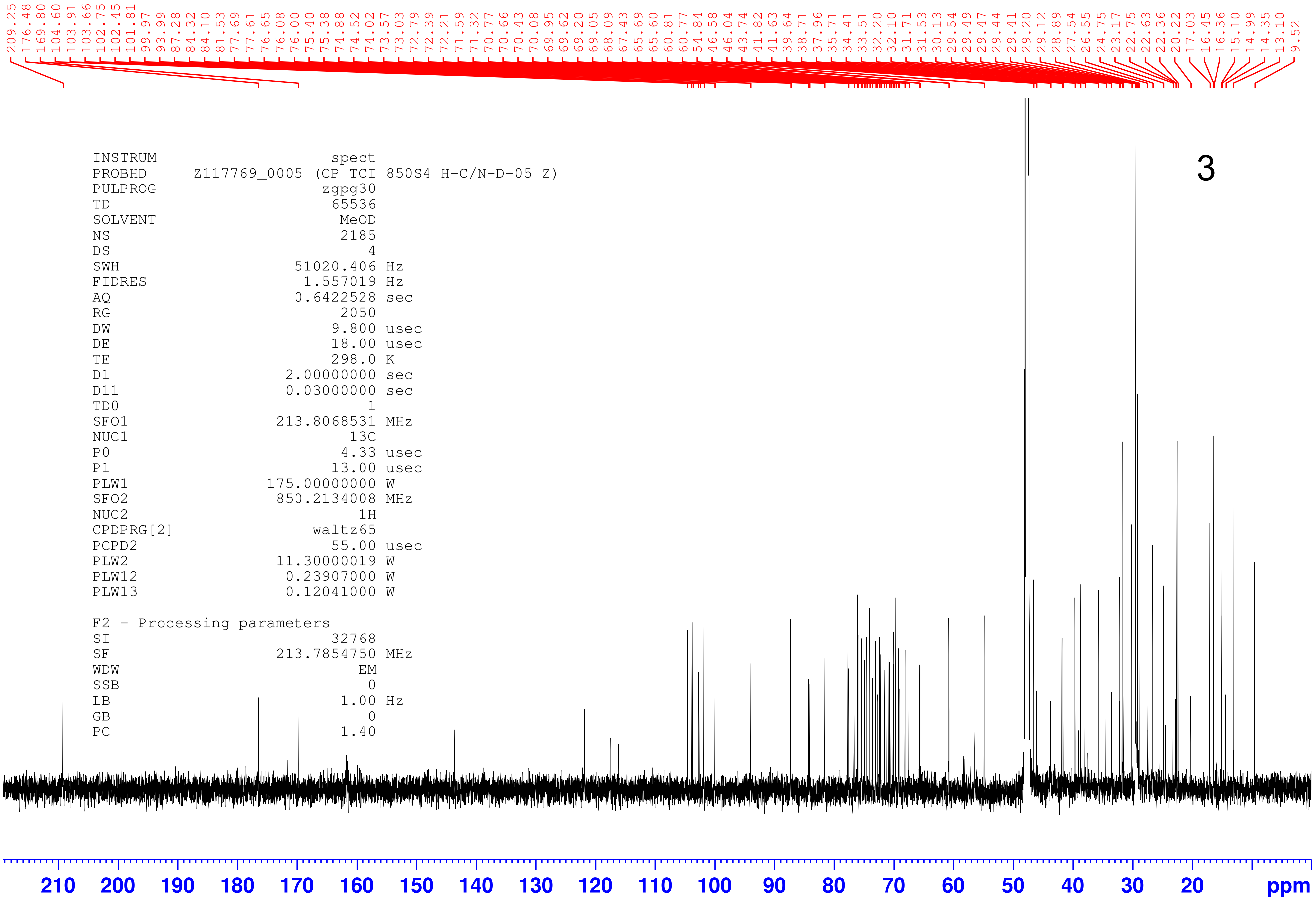
209.25
176.48
169.80
104.60
103.91
103.66
102.75
102.45
101.81
99.97
93.99
87.28
84.32
84.10
81.53
77.69
77.61
76.65
76.08
76.00
75.40
75.38
74.88
74.52
74.02
73.57
73.03
72.79
72.39
72.21
71.59
71.32
70.77
70.66
70.43
70.08
69.95
69.62
69.20
69.05
68.09
67.43
65.69
65.60
60.81
60.77
54.84
46.58
46.04
43.74
41.82
41.63
39.64
38.71
37.96
35.71
34.41
33.51
32.20
32.10
31.71
31.53
30.13
29.54
29.49
29.47
29.44
29.41
29.20
29.12
28.89
27.54
26.55
24.75
23.17
22.75
22.63
22.36
20.22
17.03
16.45
16.36
15.10
14.99
14.35
13.10
9.52
3
INSTRUM spect
PROBHD Z117769_0005 (CP TCI 850S4 H-C/N-D-05 Z)
PULPROG zgpg30
TD 65536
SOLVENT MeOD
NS 2185
DS 4
SWH 51020.406 Hz
FIDRES 1.557019 Hz
AQ 0.6422528 sec
RG 2050
DW 9.800 usec
DE 18.00 usec
TE 298.0 K
D1 2.00000000 sec
D11 0.03000000 sec
TD0 1
SFO1 213.8068531 MHz
NUC1 13C
P0 4.33 usec
P1 13.00 usec
PLW1 175.00000000 W
SFO2 850.2134008 MHz
NUC2 1H
CPDPRG[2] waltz65
PCPD2 55.00 usec
PLW2 11.30000019 W
PLW12 0.23907000 W
PLW13 0.12041000 W
F2 - Processing parameters
SI 32768
SF 213.7854750 MHz
WDW EM
SSB 0
LB 1.00 Hz
GB 0
PC 1.40
210
200
190
180
170
160
150
140
130
120
110
100
90
80
70
60
50
40
30
20
ppm

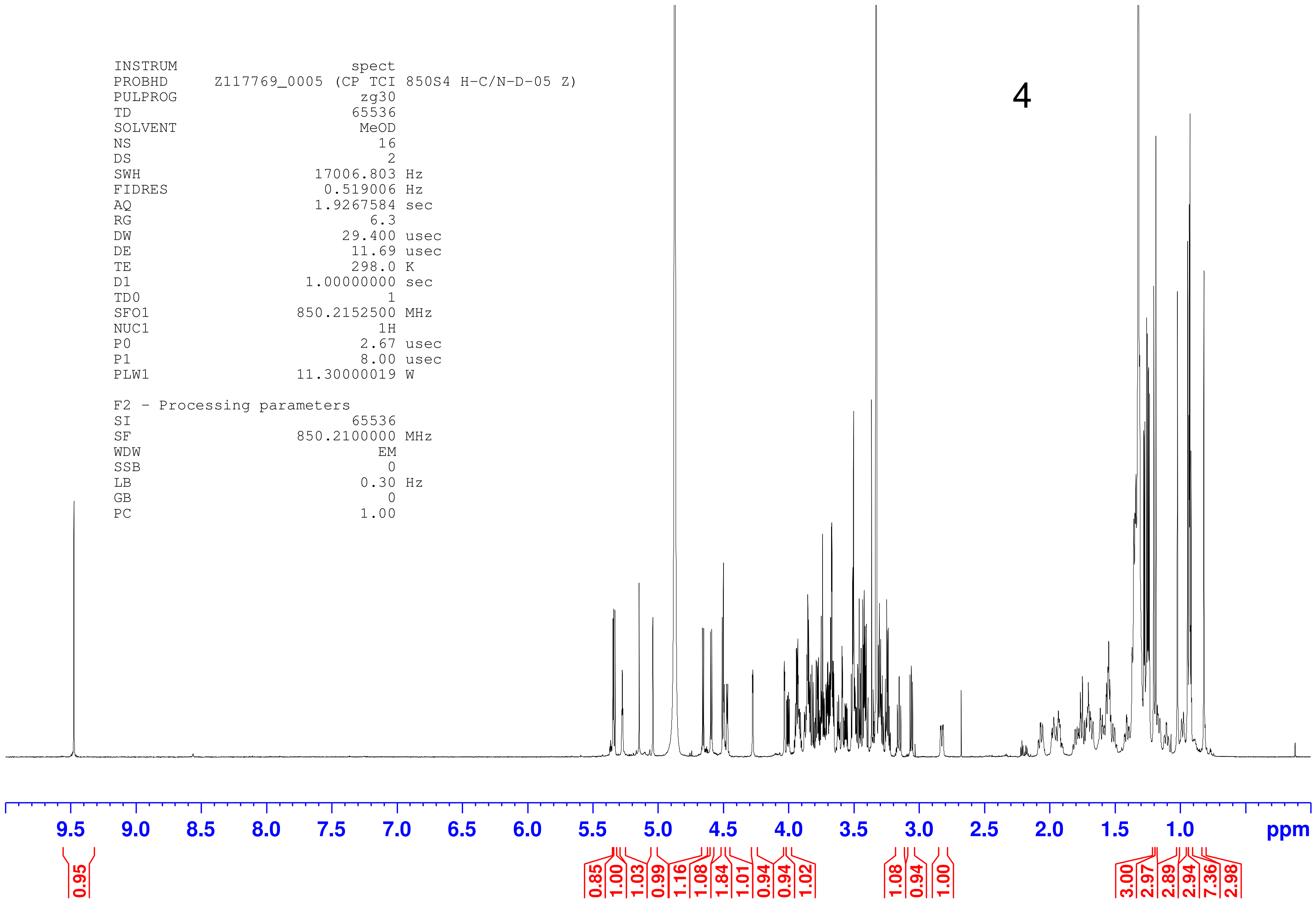

INSTRUM spect
PROBHD Z117769_0005 (CP TCI 850S4 H-C/N-D-05 Z)
PULPROG zg30
TD 65536
SOLVENT MeOD
NS 16
DS 2
SWH 17006.803 Hz
FIDRES 0.519006 Hz
AQ 1.9267584 sec
RG 6.3
DW 29.400 usec
DE 11.69 usec
TE 298.0 K
D1 1.00000000 sec
TD0 1
SFO1 850.2152500 MHz
NUC1 1H
P0 2.67 usec
P1 8.00 usec
PLW1 11.30000019 W
F2 - Processing parameters
SI 65536
SF 850.2100000 MHz
WDW EM
SSB 0
LB 0.30 Hz
GB 0
PC 1.00
4
9.5 9.0 8.5 8.0 7.5 7.0 6.5 6.0 5.5 5.0 4.5 4.0 3.5 3.0 2.5 2.0 1.5 1.0 ppm
0.95
0.85 1.00 1.03 0.99 1.16 1.08 1.84 1.01 0.94 0.94 1.02
1.08 0.94 1.00
3.00 2.97 2.89 2.94 7.36 2.98

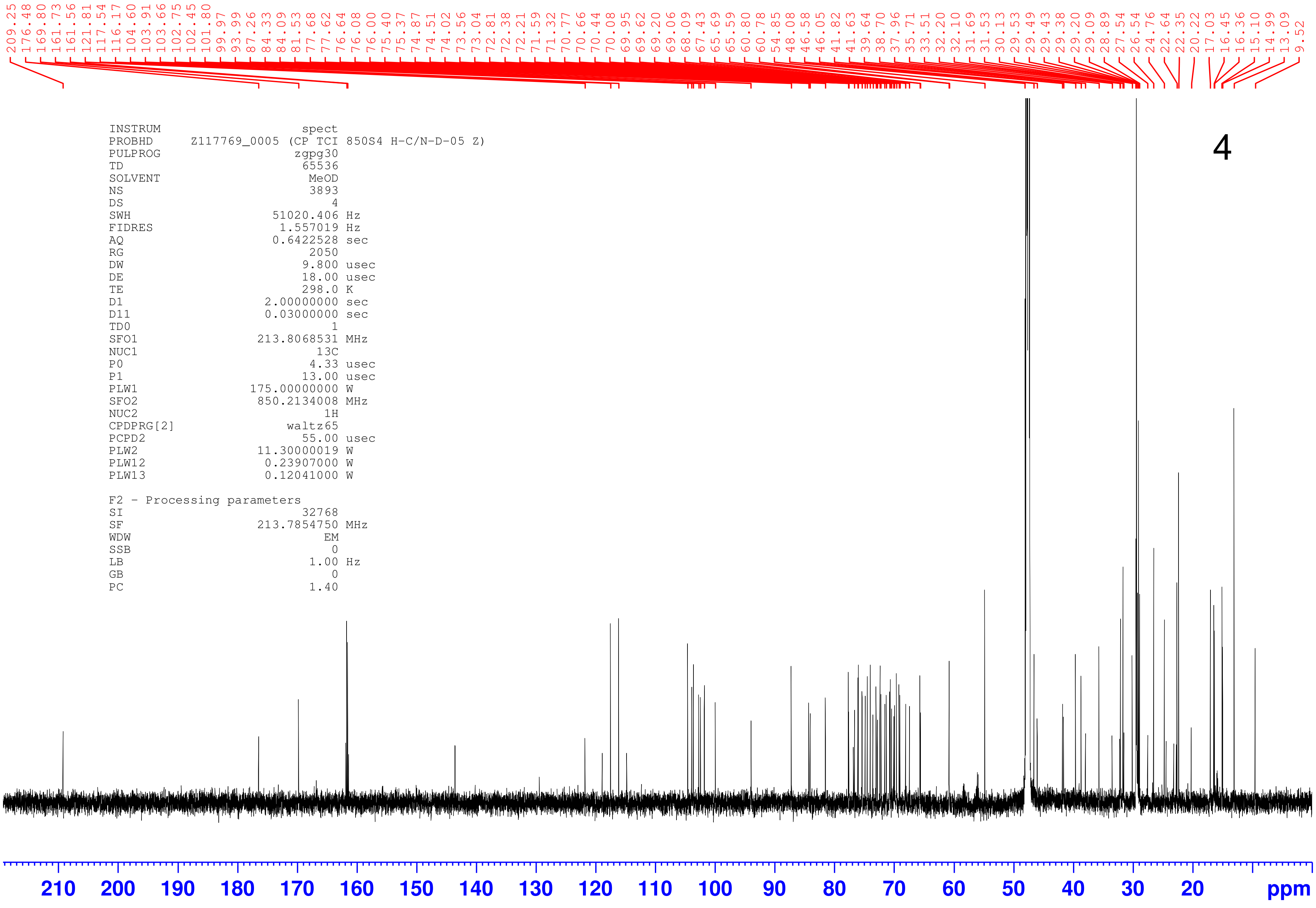
209.25 176.48 169.80 161.73 161.56 121.81 117.54 116.17 104.60 103.91 103.66 102.75 102.45 101.80 99.97 93.99 87.26 84.33 84.09 81.53 77.68 77.62 76.64 76.08 76.00 75.40 75.37 74.87 74.51 74.02 73.56 73.04 72.81 72.38 72.21 71.59 71.32 70.77 70.66 70.44 70.08 69.95 69.62 69.20 69.06 68.09 67.43 65.69 65.59 60.80 60.78 54.85 48.08 46.58 46.05 41.82 41.63 39.64 38.70 37.96 35.71 33.51 32.20 32.10 31.69 31.53 30.13 29.53 29.49 29.43 29.38 29.20 29.09 28.89 27.54 26.54 24.76 22.64 22.35 20.22 17.03 16.45 16.36 15.10 14.99 13.09 9.52
INSTRUM spect
PROBHD Z117769_0005 (CP TCI 850S4 H-C/N-D-05 Z)
PULPROG zgpg30
TD 65536
SOLVENT MeOD
NS 3893
DS 4
SWH 51020.406 Hz
FIDRES 1.557019 Hz
AQ 0.6422528 sec
RG 2050
DW 9.800 usec
DE 18.00 usec
TE 298.0 K
D1 2.00000000 sec
D11 0.03000000 sec
TD0 1
SFO1 213.8068531 MHz
NUC1 13C
P0 4.33 usec
P1 13.00 usec
PLW1 175.00000000 W
SFO2 850.2134008 MHz
NUC2 1H
CPDPRG[2] waltz65
PCPD2 55.00 usec
PLW2 11.30000019 W
PLW12 0.23907000 W
PLW13 0.12041000 W
F2 - Processing parameters
SI 32768
SF 213.7854750 MHz
WDW EM
SSB 0
LB 1.00 Hz
GB 0
PC 1.40
4
210 200 190 180 170 160 150 140 130 120 110 100 90 80 70 60 50 40 30 20 ppm

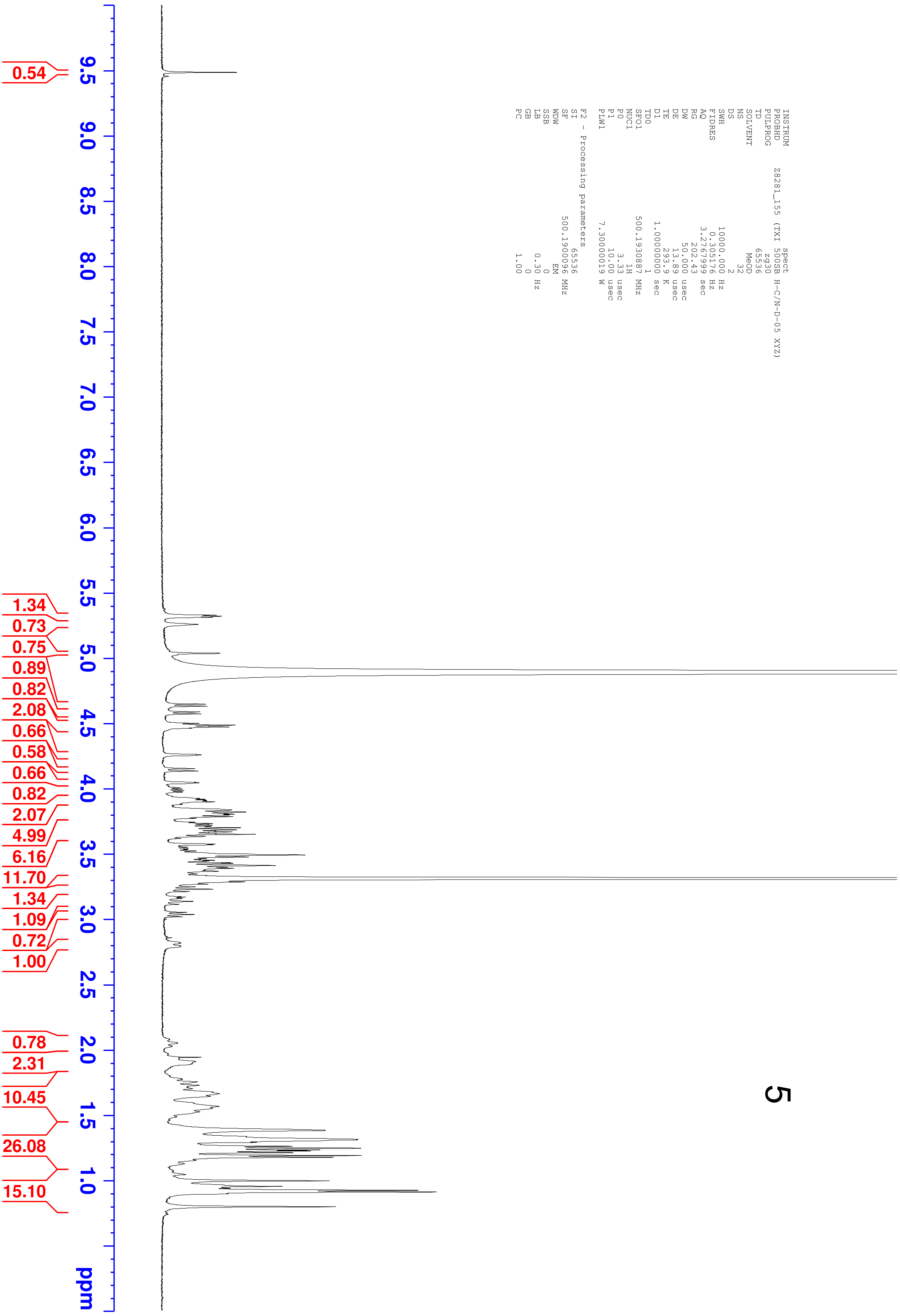
INSTRUM spect
PROBHD Z8281_155 (TXI 500SB H-C/N-D-05 XYZ)
PULPROG zg30
TD 65536
SOLVENT MeOD
NS 32
DS 2
SWH 10000.000 Hz
FIDRES 0.305176 Hz
AQ 3.2767999 sec
RG 202.43
DW 50.000 usec
DE 13.89 usec
TE 293.9 K
D1 1.00000000 sec
TD0 1
SFO1 500.1930887 MHz
NUC1 1H
P0 3.33 usec
P1 10.00 usec
PLW1 7.30000019 W
F2 - Processing parameters
SI 65536
SF 500.1900096 MHz
WDW EM
SSB 0
LB 0.30 Hz
GB 0
PC 1.00
ppm

5

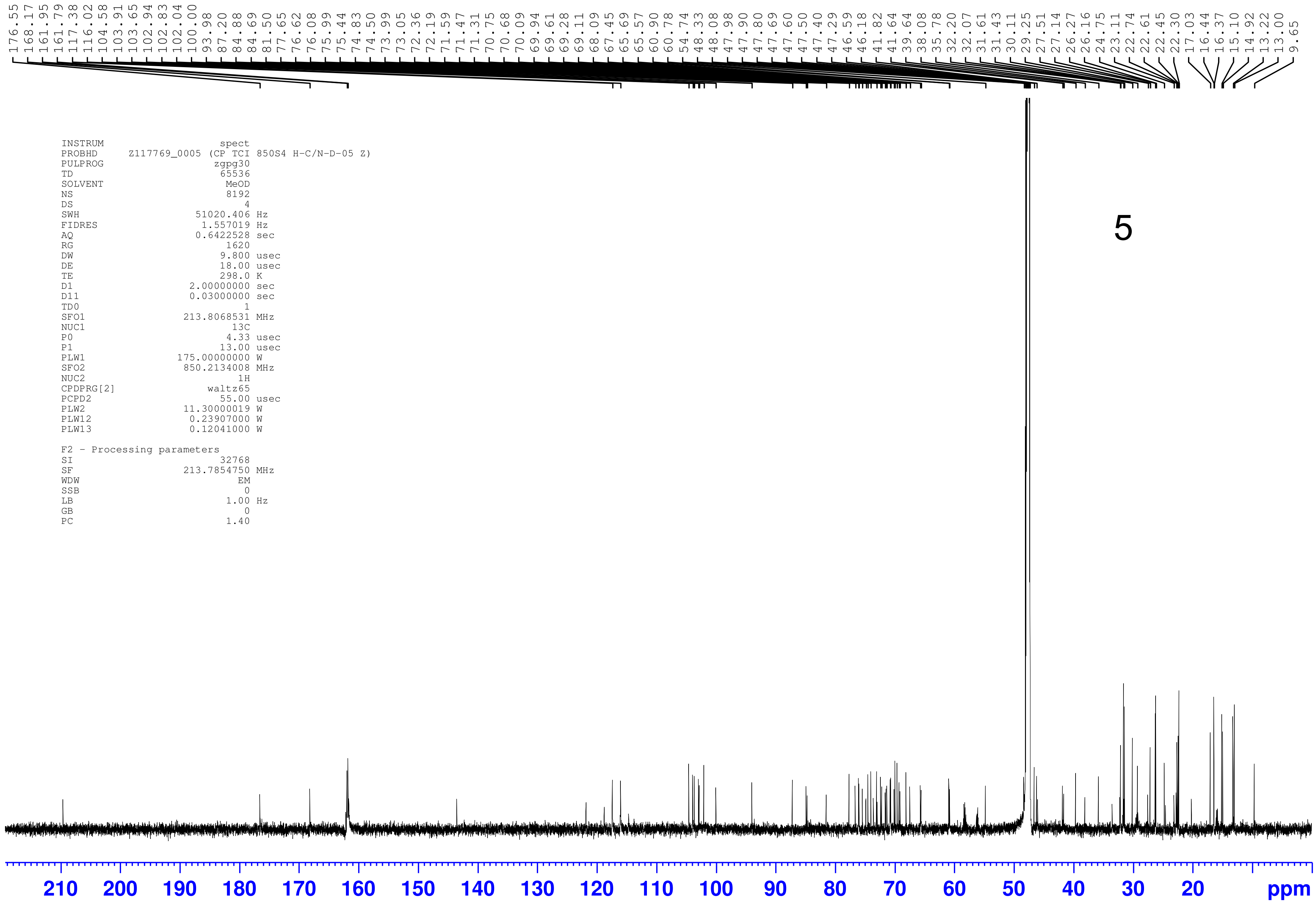
176.55
168.17
161.95
161.79
117.38
116.02
104.58
103.91
103.65
102.94
102.83
102.04
100.00
93.98
87.20
84.88
84.69
81.50
77.65
76.62
76.08
75.99
75.44
74.83
74.50
73.99
73.05
72.36
72.19
71.59
71.47
71.31
70.75
70.68
70.09
69.94
69.61
69.28
69.11
68.09
67.45
65.69
65.57
60.90
60.78
54.74
48.33
48.08
47.98
47.90
47.80
47.69
47.60
47.50
47.40
47.29
46.59
46.18
41.82
41.64
39.64
38.08
35.78
32.20
32.07
31.61
31.43
30.11
29.25
27.51
27.14
26.27
26.16
24.75
23.11
22.74
22.61
22.45
22.30
17.03
16.44
16.37
15.10
14.92
13.22
13.00
9.65
INSTRUM spect
PROBHD Z117769_0005 (CP TCI 850S4 H-C/N-D-05 Z)
PULPROG zgpg30
TD 65536
SOLVENT MeOD
NS 8192
DS 4
SWH 51020.406 Hz
FIDRES 1.557019 Hz
AQ 0.6422528 sec
RG 1620
DW 9.800 usec
DE 18.00 usec
TE 298.0 K
D1 2.00000000 sec
D11 0.03000000 sec
TD0 1
SFO1 213.8068531 MHz
NUC1 13C
P0 4.33 usec
P1 13.00 usec
PLW1 175.00000000 W
SFO2 850.2134008 MHz
NUC2 1H
CPDPRG[2] waltz65
PCPD2 55.00 usec
PLW2 11.30000019 W
PLW12 0.23907000 W
PLW13 0.12041000 W
F2 - Processing parameters
SI 32768
SF 213.7854750 MHz
WDW EM
SSB 0
LB 1.00 Hz
GB 0
PC 1.40
210 200 190 180 170 160 150 140 130 120 110 100 90 80 70 60 50 40 30 20 ppm

5

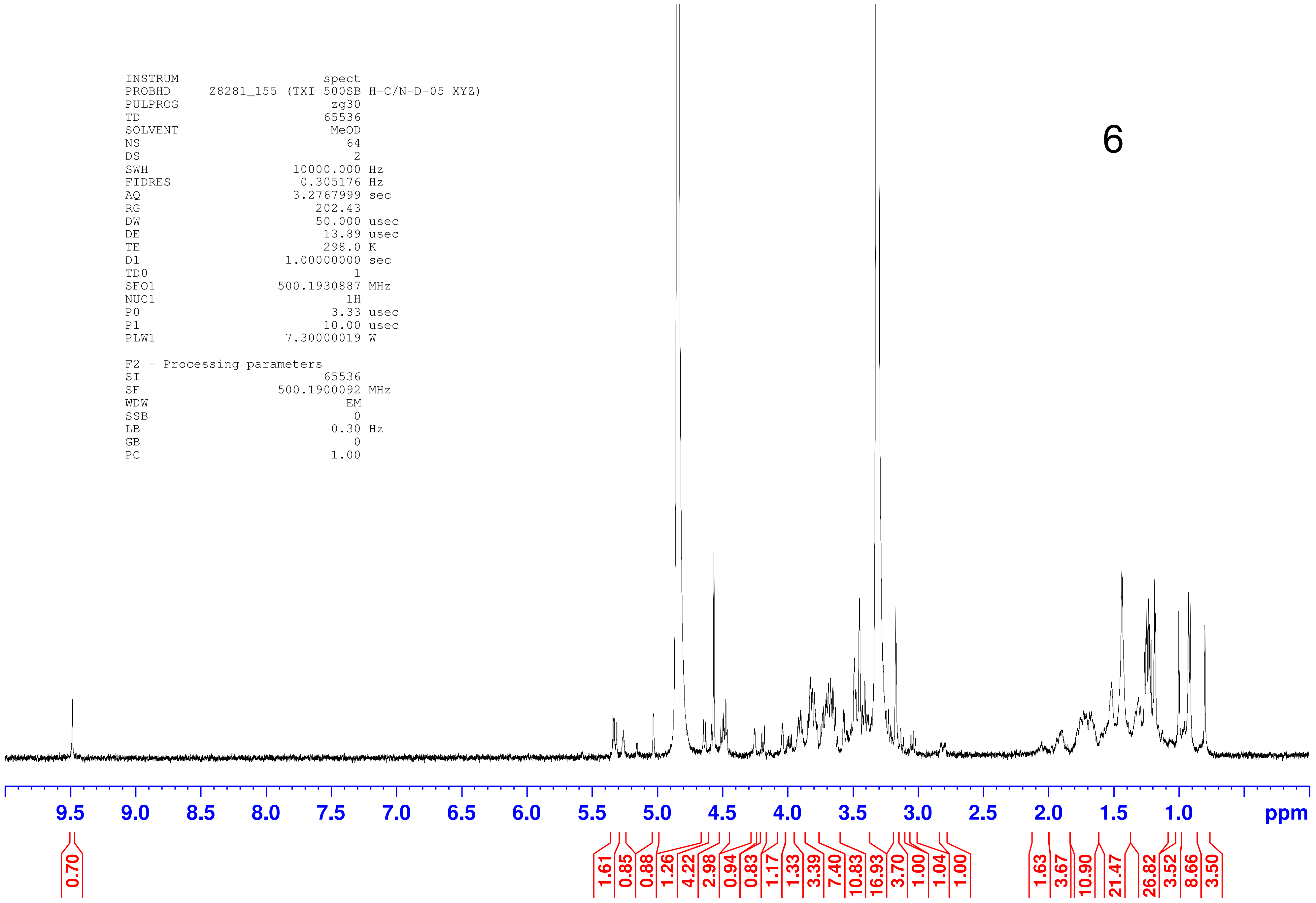
INSTRUM spect
PROBHD Z8281_155 (TXI 500SB H-C/N-D-05 XYZ)
PULPROG zg30
TD 65536
SOLVENT MeOD
NS 64
DS 2
SWH 10000.000 Hz
FIDRES 0.305176 Hz
AQ 3.2767999 sec
RG 202.43
DW 50.000 usec
DE 13.89 usec
TE 298.0 K
D1 1.00000000 sec
TD0 1
SFO1 500.1930887 MHz
NUC1 1H
P0 3.33 usec
P1 10.00 usec
PLW1 7.30000019 W
F2 - Processing parameters
SI 65536
SF 500.1900092 MHz
WDW EM
SSB 0
LB 0.30 Hz
GB 0
PC 1.00
6
9.5 9.0 8.5 8.0 7.5 7.0 6.5 6.0 5.5 5.0 4.5 4.0 3.5 3.0 2.5 2.0 1.5 1.0 ppm
0.70
1.61 0.85 0.88 1.26 4.22 2.98 0.94 0.83 1.17 1.33 3.39 7.40 10.83 16.93 3.70 1.00 1.04 1.00
1.63 3.67 10.90 21.47 26.82 3.52 8.66 3.50

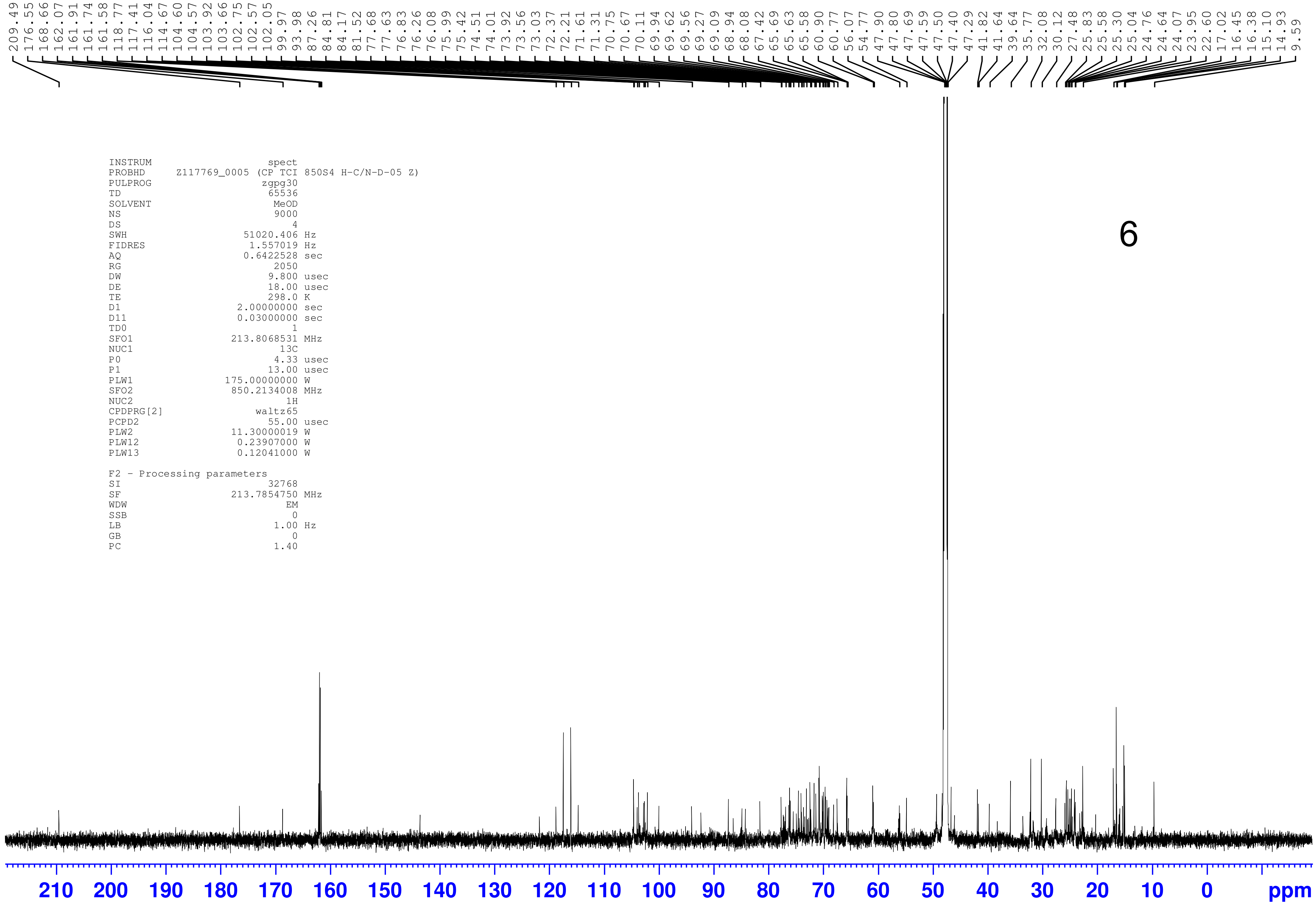
209.49
176.55
168.66
162.07
161.91
161.74
161.58
118.77
117.41
116.04
114.67
104.60
104.57
103.92
103.66
102.75
102.57
102.05
99.97
93.98
87.26
84.81
84.17
81.52
77.68
77.63
76.83
76.26
76.08
75.99
75.42
74.51
74.01
73.92
73.56
73.03
72.37
72.21
71.61
71.31
70.75
70.67
70.11
69.94
69.62
69.56
69.27
69.09
68.94
68.08
67.42
65.69
65.63
65.58
60.90
60.77
56.07
54.77
47.90
47.80
47.69
47.59
47.50
47.40
47.29
41.82
41.64
39.64
35.77
32.08
30.12
27.48
25.83
25.58
25.30
25.04
24.76
24.64
24.07
23.95
22.60
17.02
16.45
16.38
15.10
14.93
9.59
INSTRUM spect
PROBHD Z117769_0005 (CP TCI 850S4 H-C/N-D-05 Z)
PULPROG zgpg30
TD 65536
SOLVENT MeOD
NS 9000
DS 4
SWH 51020.406 Hz
FIDRES 1.557019 Hz
AQ 0.6422528 sec
RG 2050
DW 9.800 usec
DE 18.00 usec
TE 298.0 K
D1 2.00000000 sec
D11 0.03000000 sec
TD0 1
SFO1 213.8068531 MHz
NUC1 13C
P0 4.33 usec
P1 13.00 usec
PLW1 175.00000000 W
SFO2 850.2134008 MHz
NUC2 1H
CPDPRG[2] waltz65
PCPD2 55.00 usec
PLW2 11.30000019 W
PLW12 0.23907000 W
PLW13 0.12041000 W
F2 - Processing parameters
SI 32768
SF 213.7854750 MHz
WDW EM
SSB 0
LB 1.00 Hz
GB 0
PC 1.40
210
200
190
180
170
160
150
140
130
120
110
100
90
80
70
60
50
40
30
20
10
0
ppm

6

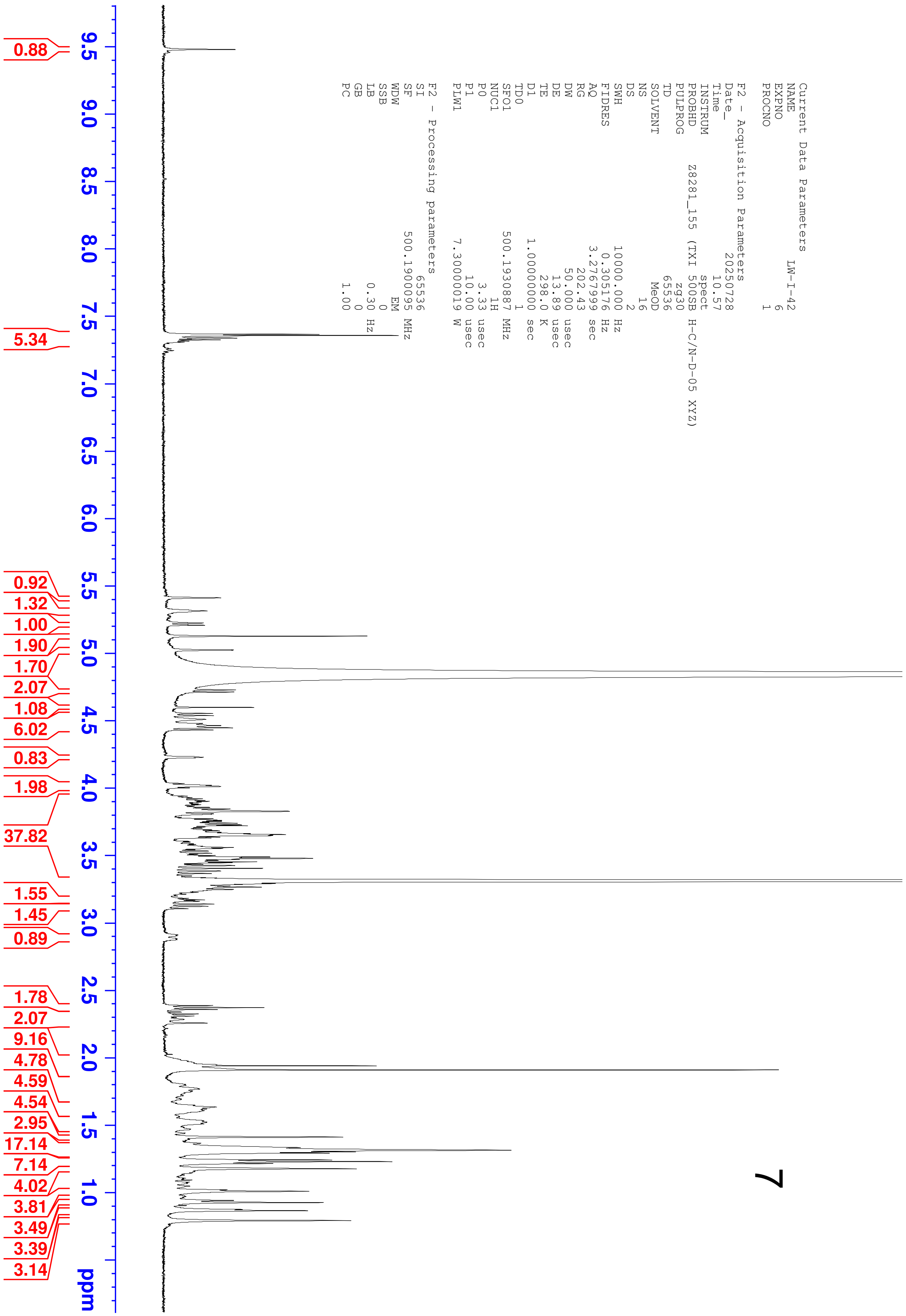

Current Data Parameters
NAME LW-I-42
EXPNO 6
PROCNO 1
F2 - Acquisition Parameters
Date_ 20250728
Time 10.57
INSTRUM spect
PROBHD Z8281_155 (TXI 500SB H-C/N-D-05 XYZ)
PULPROG zg30
TD 65536
SOLVENT MeOD
NS 16
DS 2
SWH 10000.000 Hz
FIDRES 0.305176 Hz
AQ 3.2767999 sec
RG 202.43
DW 50.000 usec
DE 13.89 usec
TE 298.0 K
D1 1.00000000 sec
TD0 1
SFO1 500.1930887 MHz
NUC1 1H
P0 3.33 usec
P1 10.00 usec
PLW1 7.30000019 W
F2 - Processing parameters
SI 65536
SF 500.1900095 MHz
WDW EM
SSB 0
LB 0.30 Hz
GB 0
PC 1.00
7
ppm

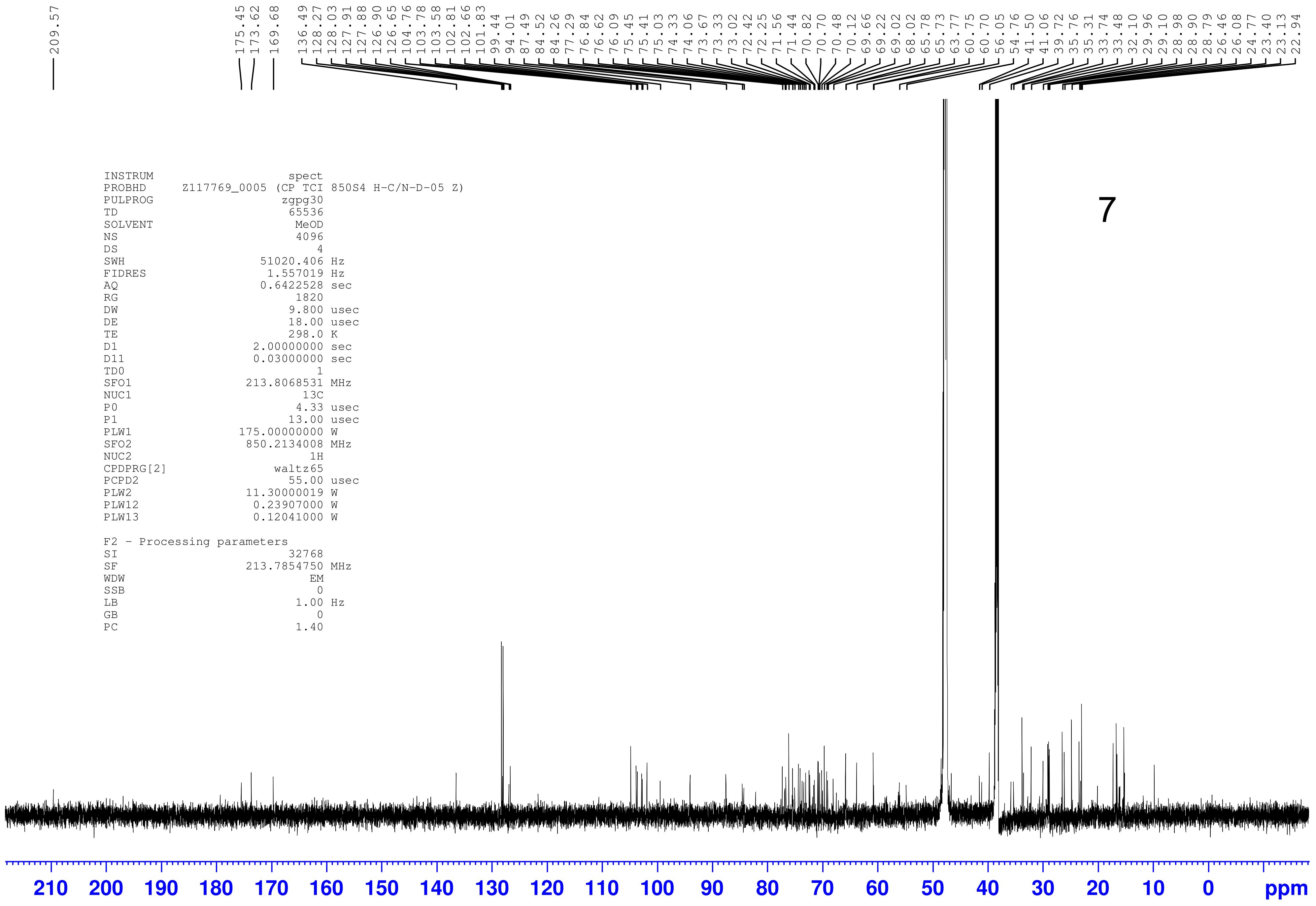

7

INSTRUM	spect	
PROBHD	Z117769_0005 (CP TCI 850S4 H-C/N-D-05 Z)	
PULPROG	zgpg30	
TD	65536	
SOLVENT	MeOD	
NS	4096	
DS	4	
SWH	51020.406	Hz
FIDRES	1.557019	Hz
AQ	0.6422528	sec
RG	1820	
DW	9.800	usec
DE	18.00	usec
TE	298.0	K
D1	2.00000000	sec
D11	0.03000000	sec
TD0	1	
SFO1	213.8068531	MHz
NUC1	13C	
P0	4.33	usec
P1	13.00	usec
PLW1	175.00000000	W
SFO2	850.2134008	MHz
NUC2	1H	
CPDPRG[2]	waltz65	
PCPD2	55.00	usec
PLW2	11.30000019	W
PLW12	0.23907000	W
PLW13	0.12041000	W

F2 - Processing parameters

SI	32768	
SF	213.7854750	MHz
WDW	EM	
SSB	0	
LB	1.00	Hz
GB	0	
PC	1.40	

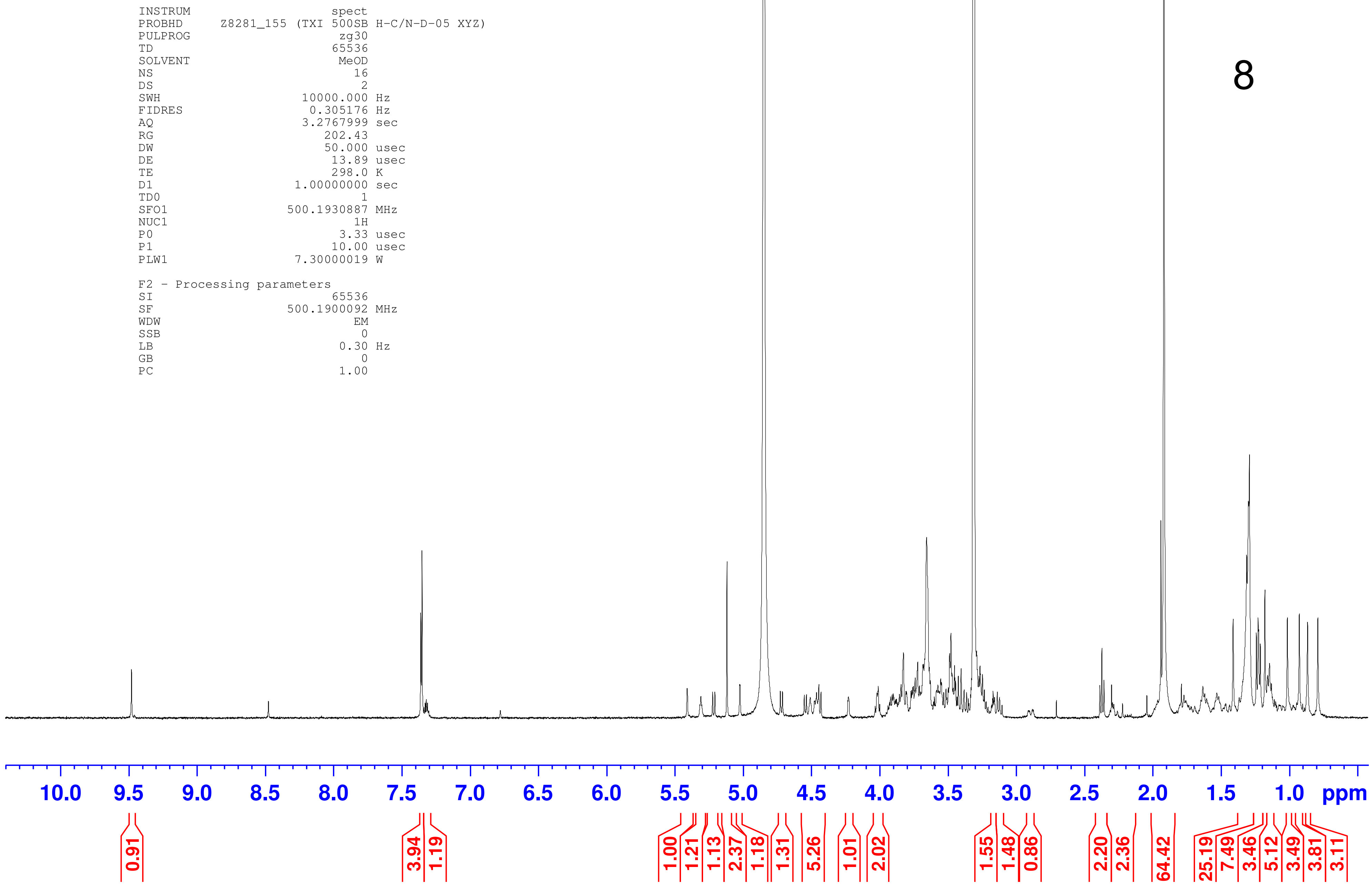
INSTRUM spect
PROBHD Z8281_155 (TXI 500SB H-C/N-D-05 XYZ)
PULPROG zg30
TD 65536
SOLVENT MeOD
NS 16
DS 2
SWH 10000.000 Hz
FIDRES 0.305176 Hz
AQ 3.2767999 sec
RG 202.43
DW 50.000 usec
DE 13.89 usec
TE 298.0 K
D1 1.00000000 sec
TD0 1
SFO1 500.1930887 MHz
NUC1 1H
P0 3.33 usec
P1 10.00 usec
PLW1 7.30000019 W
F2 - Processing parameters
SI 65536
SF 500.1900092 MHz
WDW EM
SSB 0
LB 0.30 Hz
GB 0
PC 1.00
10.0 9.5 9.0 8.5 8.0 7.5 7.0 6.5 6.0 5.5 5.0 4.5 4.0 3.5 3.0 2.5 2.0 1.5 1.0 ppm
0.91 3.94 1.19 1.00 1.21 1.13 2.37 1.18 1.31 5.26 1.01 2.02 1.55 1.48 0.86 2.20 2.36 64.42 25.19 7.49 3.46 5.12 3.49 3.81 3.11

8

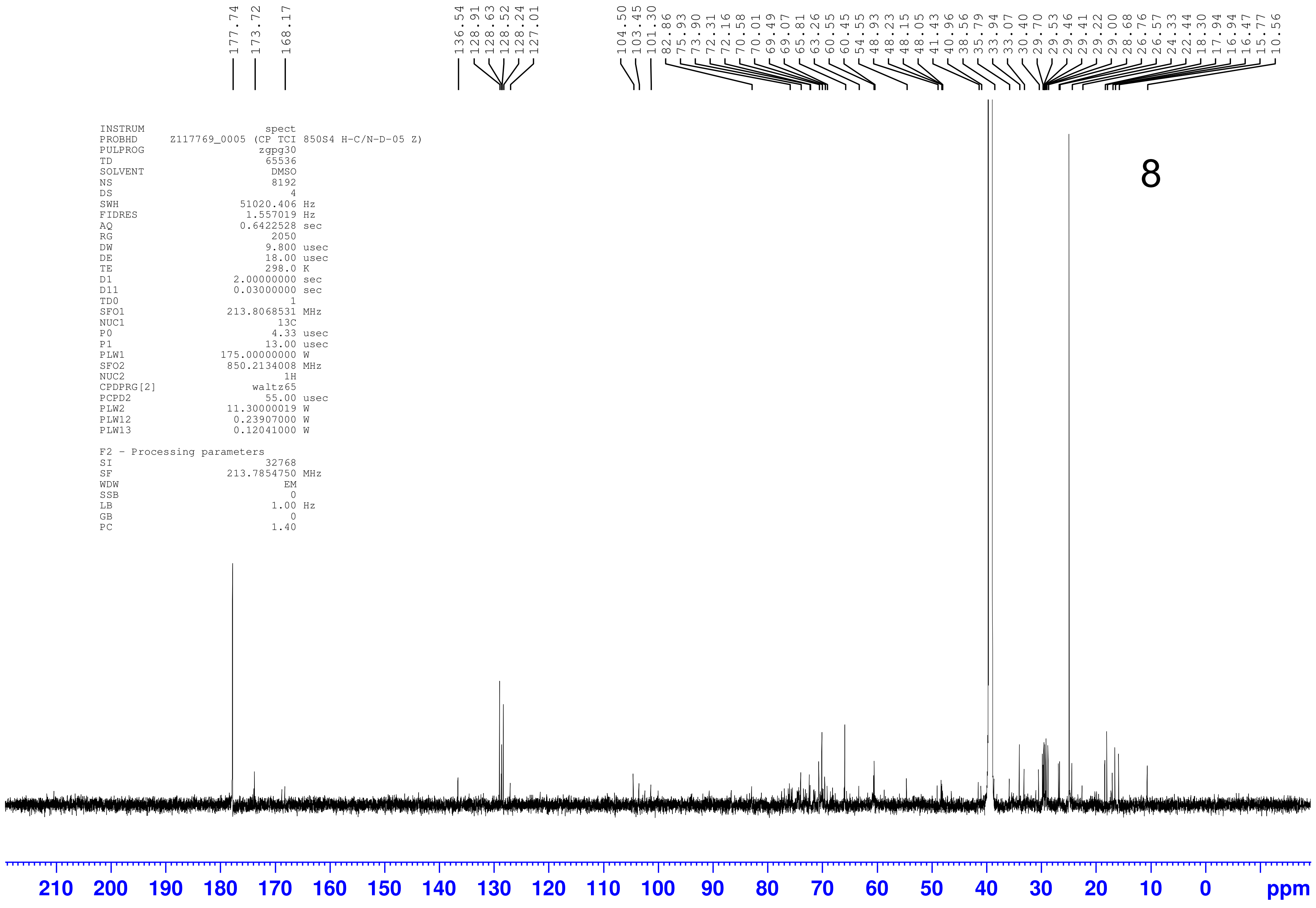
177.74
173.72
168.17
136.54
128.91
128.63
128.52
128.24
127.01
104.50
103.45
101.30
82.86
75.93
73.90
72.31
72.16
70.58
70.01
69.49
69.07
65.81
63.26
60.55
60.45
54.55
48.93
48.23
48.15
48.05
41.43
40.96
38.56
35.79
33.94
33.07
30.40
29.70
29.53
29.46
29.41
29.22
29.00
28.68
26.76
26.57
24.33
22.44
18.30
17.94
16.94
16.47
15.77
10.56
INSTRUM spect
PROBHD Z117769_0005 (CP TCI 850S4 H-C/N-D-05 Z)
PULPROG zgpg30
TD 65536
SOLVENT DMSO
NS 8192
DS 4
SWH 51020.406 Hz
FIDRES 1.557019 Hz
AQ 0.6422528 sec
RG 2050
DW 9.800 usec
DE 18.00 usec
TE 298.0 K
D1 2.00000000 sec
D11 0.03000000 sec
TD0 1
SFO1 213.8068531 MHz
NUC1 13C
P0 4.33 usec
P1 13.00 usec
PLW1 175.00000000 W
SFO2 850.2134008 MHz
NUC2 1H
CPDPRG[2] waltz65
PCPD2 55.00 usec
PLW2 11.30000019 W
PLW12 0.23907000 W
PLW13 0.12041000 W
F2 - Processing parameters
SI 32768
SF 213.7854750 MHz
WDW EM
SSB 0
LB 1.00 Hz
GB 0
PC 1.40
8
210 200 190 180 170 160 150 140 130 120 110 100 90 80 70 60 50 40 30 20 10 0 ppm

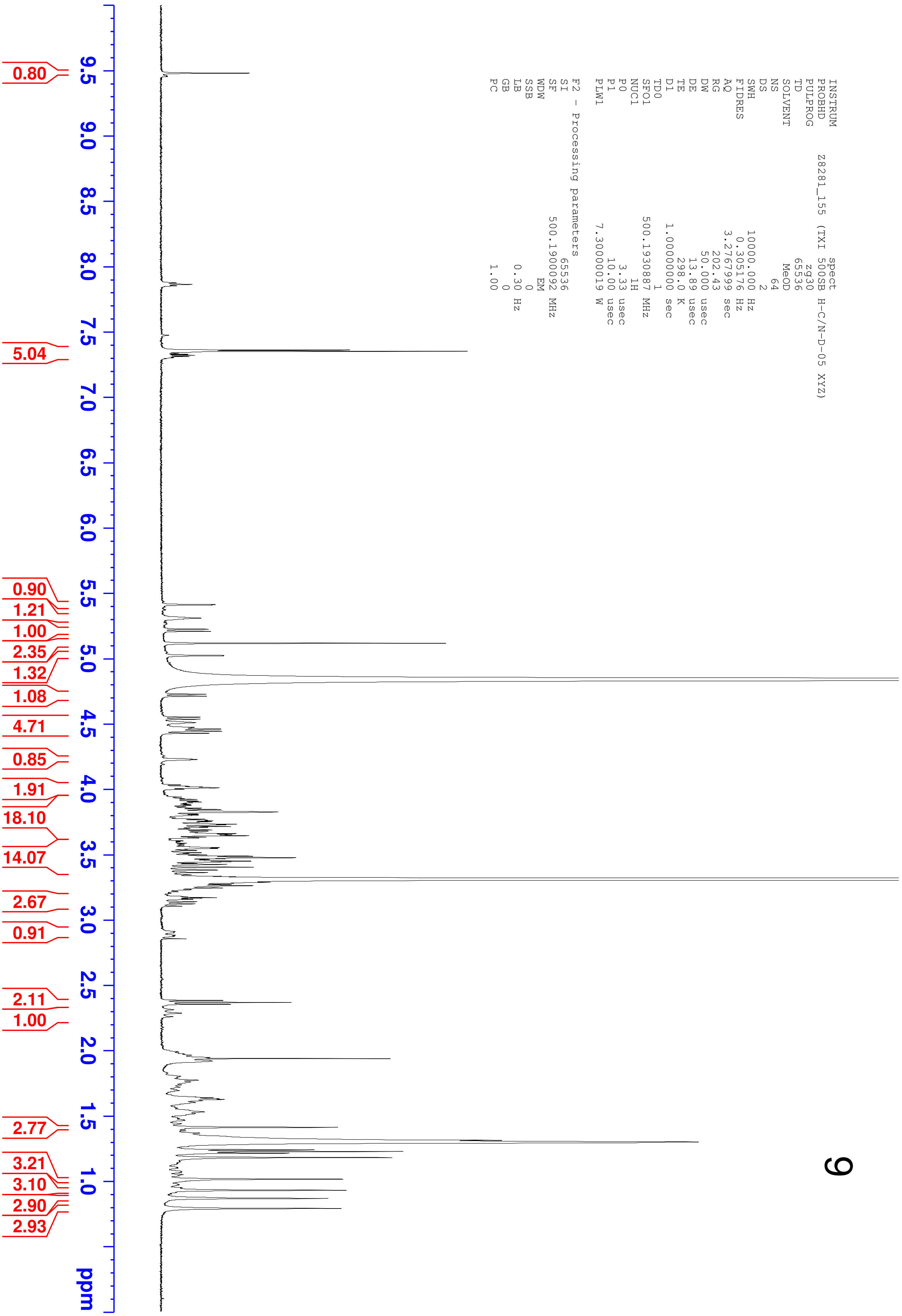
INSTRUM spect
PROBHD Z8281_155 (TXI 500SB H-C/N-D-05 XYZ)
PULPROG zg30
TD 65536
SOLVENT MeOD
NS 64
DS 2
SWH 10000.000 Hz
FIDRES 0.305176 Hz
AQ 3.2767999 sec
RG 202.43
DW 50.000 usec
DE 13.89 usec
TE 298.0 K
D1 1.00000000 sec
TD0 1
SFO1 500.1930887 MHz
NUC1 1H
P0 3.33 usec
P1 10.00 usec
PLW1 7.30000019 W
F2 - Processing parameters
SI 65536
SF 500.1900092 MHz
WDW EM
SSB 0
LB 0.30 Hz
GB 0
PC 1.00
9.5 9.0 8.5 8.0 7.5 7.0 6.5 6.0 5.5 5.0 4.5 4.0 3.5 3.0 2.5 2.0 1.5 1.0 ppm
0.80 5.04 0.90 1.21 1.00 2.35 1.32 1.08 4.71 0.85 1.91 18.10 14.07 2.67 0.91 2.11 1.00 2.77 3.21 3.10 2.90 2.93

9

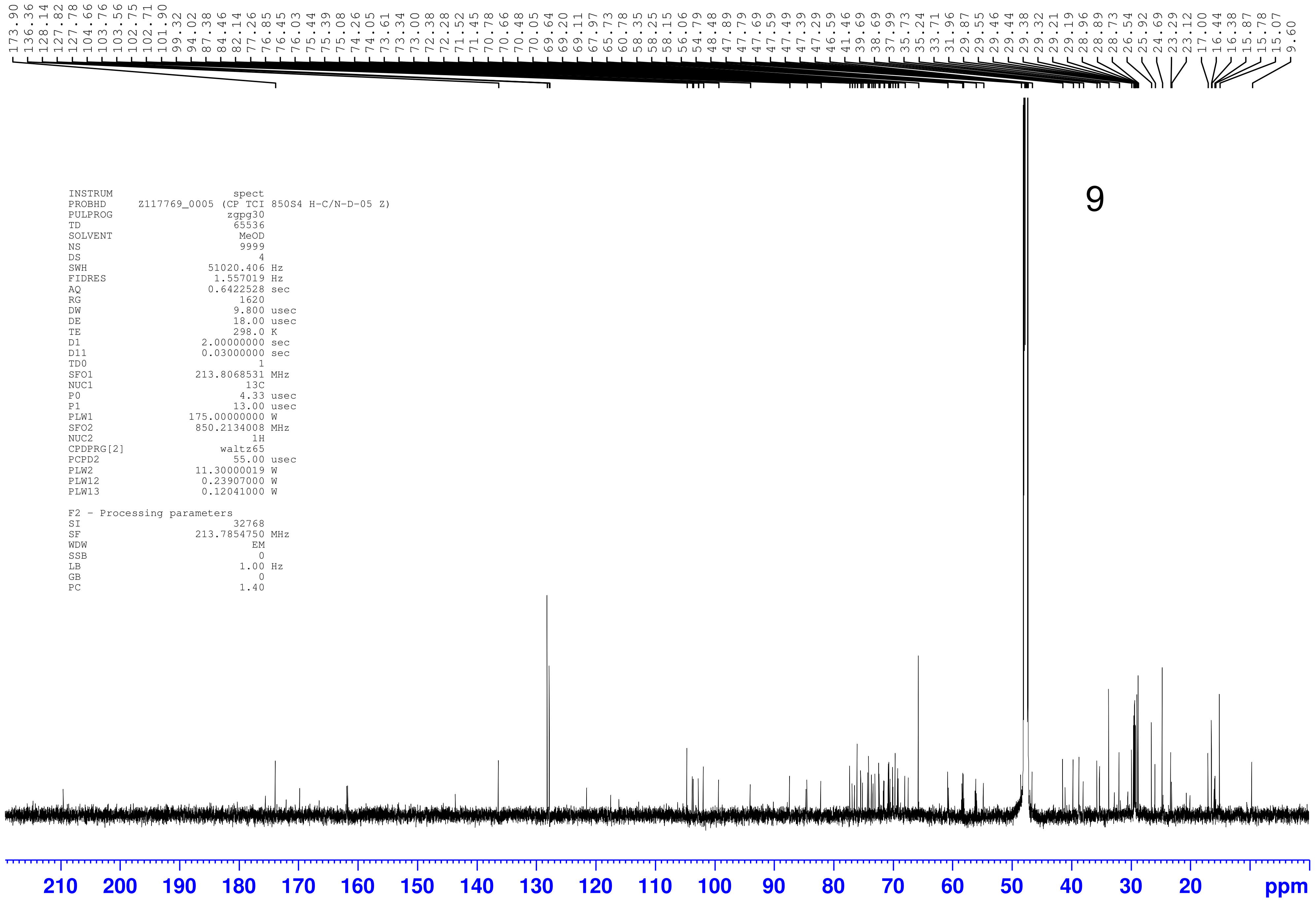
173.90
136.36
128.14
127.82
127.78
104.66
103.76
103.56
102.75
102.71
101.90
99.32
94.02
87.38
84.46
82.14
77.26
76.85
76.45
76.03
75.44
75.39
75.08
74.26
74.05
73.61
73.34
73.00
72.38
72.28
71.52
71.45
70.78
70.66
70.48
70.05
69.64
69.20
69.11
67.97
65.73
60.78
58.35
58.25
58.15
56.06
54.79
48.48
47.89
47.79
47.69
47.59
47.49
47.39
47.29
46.59
41.46
39.69
38.69
37.99
35.73
35.24
33.71
31.96
29.87
29.55
29.46
29.44
29.38
29.32
29.21
29.19
28.96
28.89
28.73
26.54
25.92
24.69
23.29
23.12
17.00
16.44
16.38
15.87
15.78
15.07
9.60
9
INSTRUM spect
PROBHD Z117769_0005 (CP TCI 850S4 H-C/N-D-05 Z)
PULPROG zgpg30
TD 65536
SOLVENT MeOD
NS 9999
DS 4
SWH 51020.406 Hz
FIDRES 1.557019 Hz
AQ 0.6422528 sec
RG 1620
DW 9.800 usec
DE 18.00 usec
TE 298.0 K
D1 2.00000000 sec
D11 0.03000000 sec
TD0 1
SFO1 213.8068531 MHz
NUC1 13C
P0 4.33 usec
P1 13.00 usec
PLW1 175.00000000 W
SFO2 850.2134008 MHz
NUC2 1H
CPDPRG[2] waltz65
PCPD2 55.00 usec
PLW2 11.30000019 W
PLW12 0.23907000 W
PLW13 0.12041000 W
F2 - Processing parameters
SI 32768
SF 213.7854750 MHz
WDW EM
SSB 0
LB 1.00 Hz
GB 0
PC 1.40
210 200 190 180 170 160 150 140 130 120 110 100 90 80 70 60 50 40 30 20 ppm

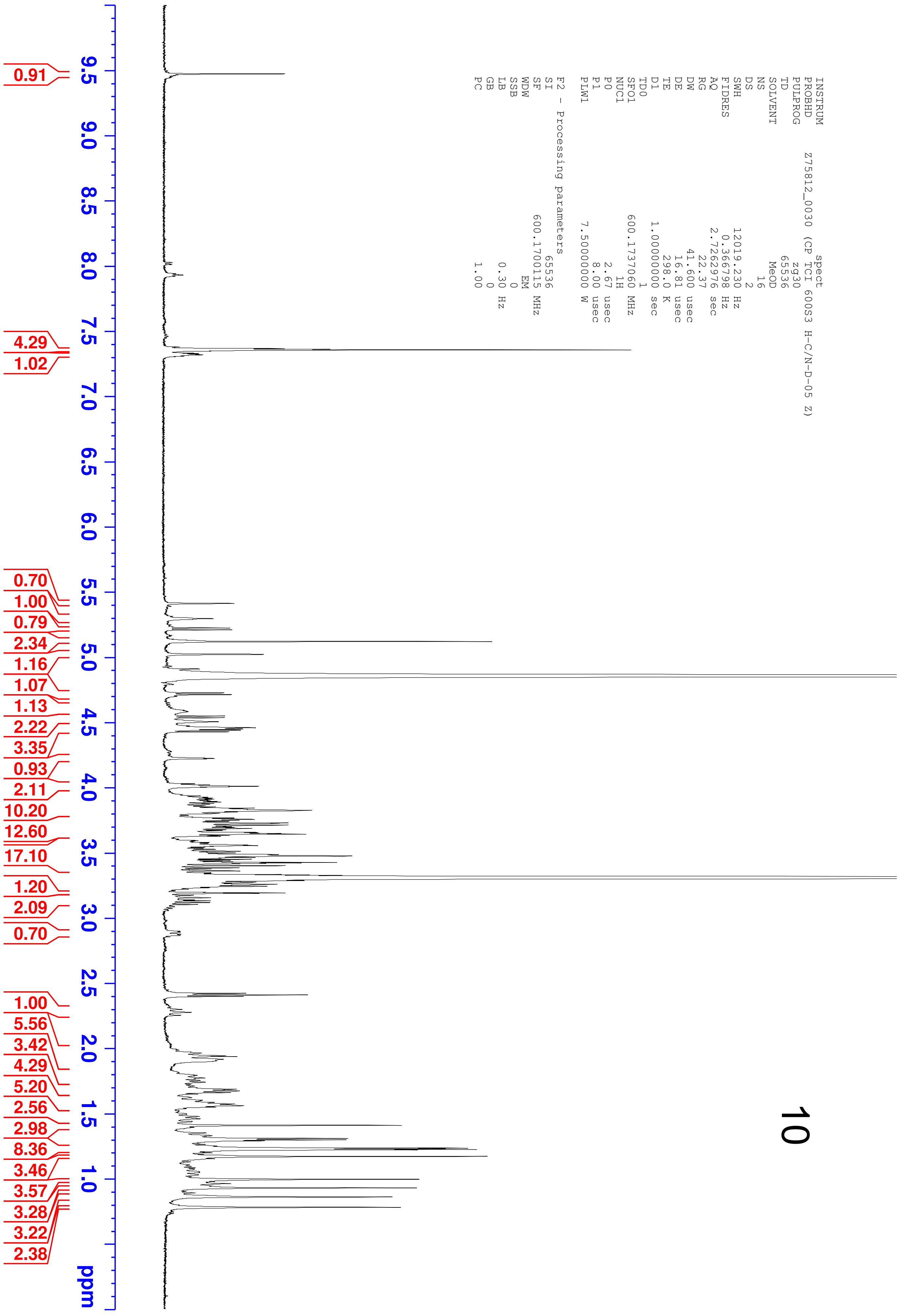
INSTRUM spect
PROBHD Z75812_0030 (CP TCI 600S3 H-C/N-D-05 Z)
PULPROG zg30
TD 65536
SOLVENT MeOD
NS 16
DS 2
SWH 12019.230 Hz
FIDRES 0.366798 Hz
AQ 2.7262976 sec
RG 22.37
DW 41.600 usec
DE 16.81 usec
TE 298.0 K
D1 1.00000000 sec
TD0 1
SFO1 600.1737060 MHz
NUC1 1H
P0 2.67 usec
P1 8.00 usec
PLW1 7.50000000 W
F2 - Processing parameters
SI 65536
SF 600.1700115 MHz
WDW EM
SSB 0
LB 0.30 Hz
GB 0
PC 1.00
9.5
9.0
8.5
8.0
7.5
7.0
6.5
6.0
5.5
5.0
4.5
4.0
3.5
3.0
2.5
2.0
1.5
1.0
ppm
0.91
4.29
1.02
0.70
1.00
0.79
2.34
1.16
1.07
1.13
2.22
3.35
0.93
2.11
10.20
12.60
17.10
1.20
2.09
0.70
1.00
5.56
3.42
4.29
5.20
2.56
2.98
8.36
3.46
3.57
3.28
3.22
2.38

10

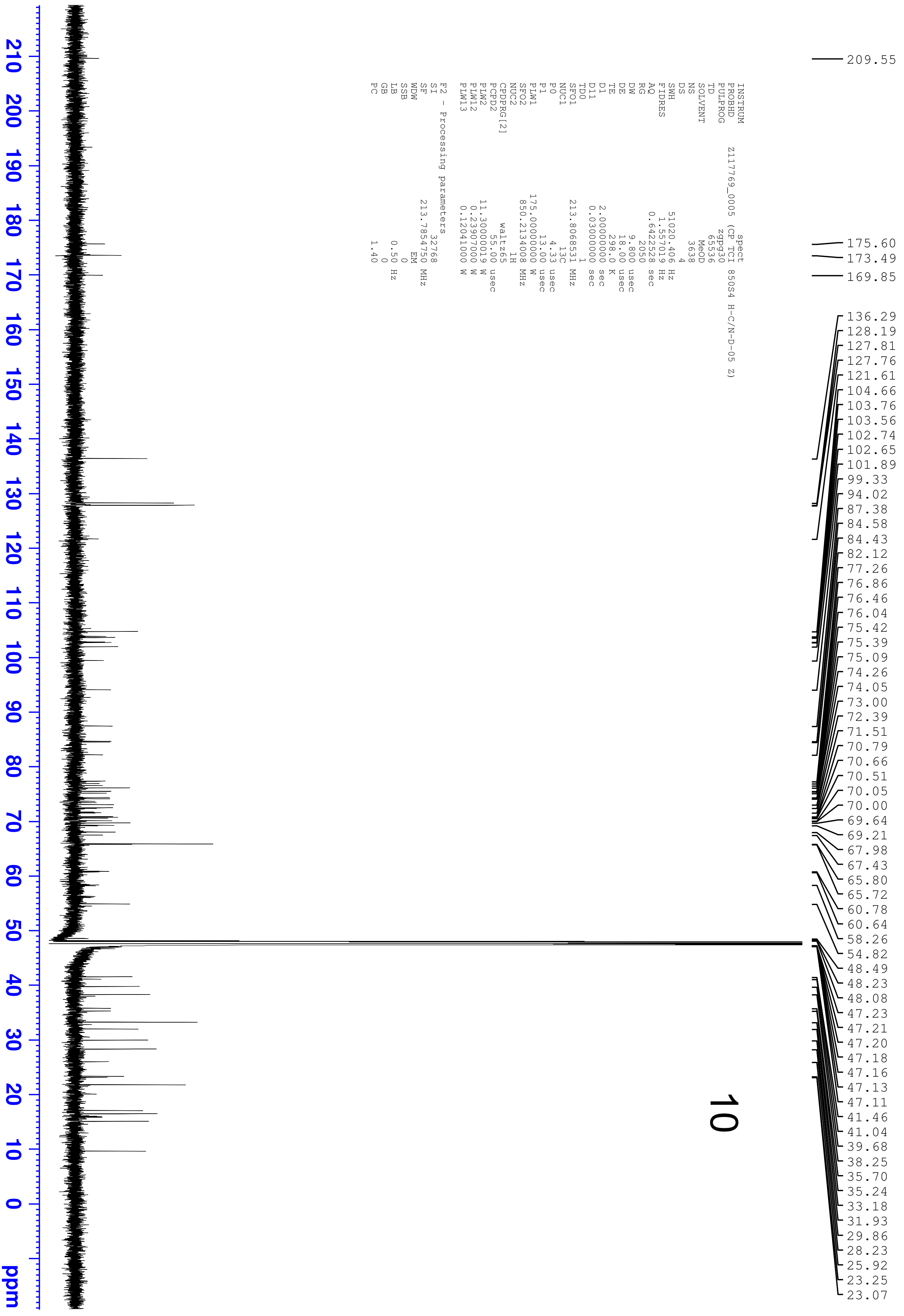

10
INSTRUM spect
PROBHD Z117769_0005 (CP TCI 850S4 H-C/N-D-05 Z)
PULPROG zgpg30
TD 65536
SOLVENT MeOD
NS 3638
DS 4
SWH 51020.406 Hz
FIDRES 1.557019 Hz
AQ 0.6422528 sec
RG 2050
DW 9.800 usec
DE 18.00 usec
TE 298.0 K
D1 2.00000000 sec
D11 0.03000000 sec
TD0 1
SFO1 213.8068531 MHz
NUC1 13C
P0 4.33 usec
P1 13.00 usec
PLW1 175.00000000 W
SFO2 850.2134008 MHz
NUC2 1H
CPDPRG[2] waltz65
PCPD2 55.00 usec
PLW2 11.30000019 W
PLW12 0.23907000 W
PLW13 0.12041000 W
F2 - Processing parameters
SI 32768
SF 213.7854750 MHz
WDW EM
SSB 0
LB 0.50 Hz
GB 0
PC 1.40
209.55
175.60
173.49
169.85
136.29
128.19
127.81
127.76
121.61
104.66
103.76
103.56
102.74
102.65
101.89
99.33
94.02
87.38
84.58
84.43
82.12
77.26
76.86
76.46
76.04
75.42
75.39
75.09
74.26
74.05
73.00
72.39
71.51
70.79
70.66
70.51
70.05
70.00
69.64
69.21
67.98
67.43
65.80
65.72
60.78
60.64
58.26
54.82
48.49
48.23
48.08
47.23
47.21
47.20
47.18
47.16
47.13
47.11
41.46
41.04
39.68
38.25
35.70
35.24
33.18
31.93
29.86
28.23
25.92
23.25
23.07
210 200 190 180 170 160 150 140 130 120 110 100 90 80 70 60 50 40 30 20 10 0 ppm

11

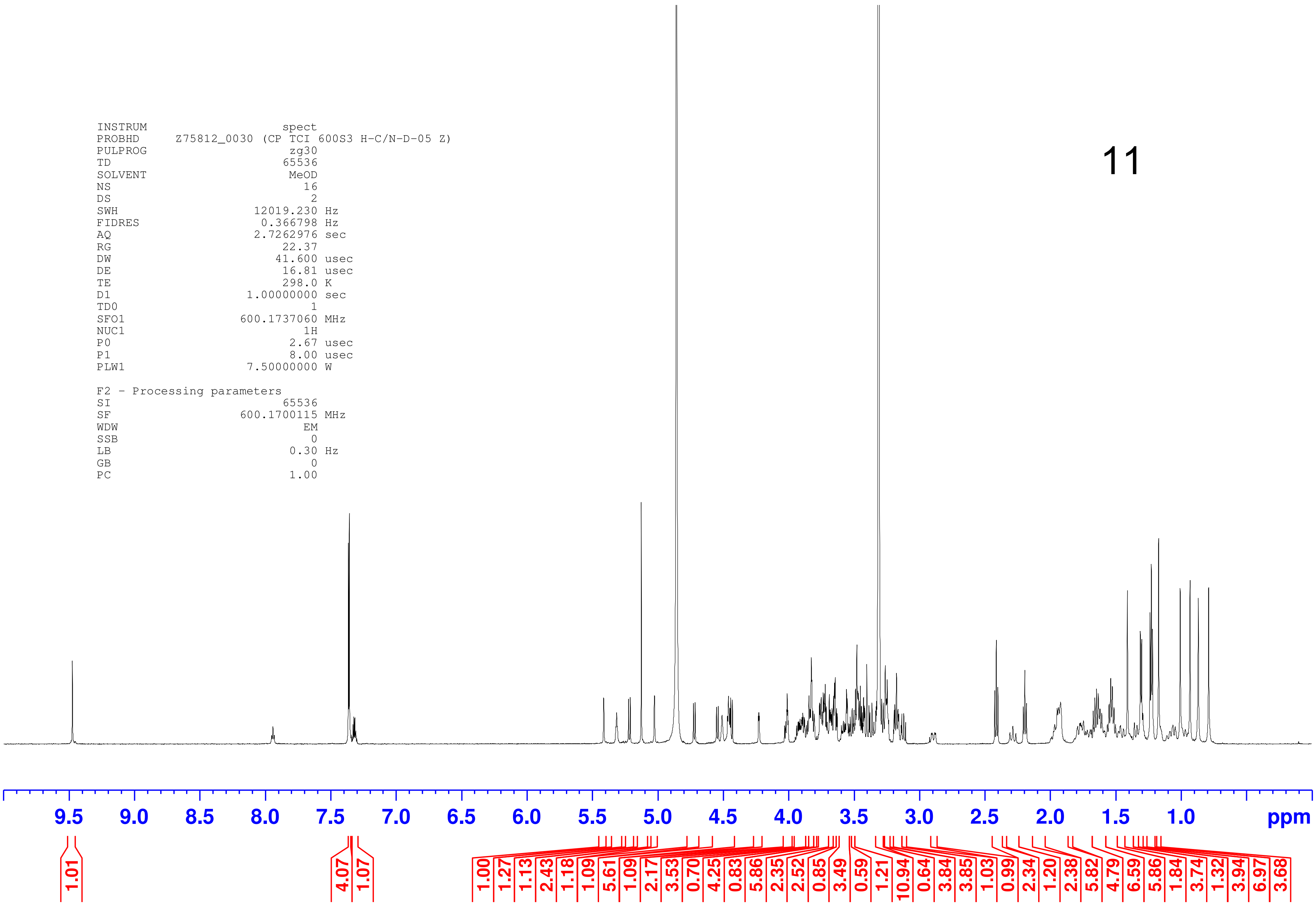


```
INSTRUM                 spect
PROBHD      Z75812_0030 (CP TCI 600S3 H-C/N-D-05 Z)
PULPROG                 zg30
TD                      65536
SOLVENT                 MeOD
NS                      16
DS                      2
SWH                     12019.230 Hz
FIDRES                  0.366798 Hz
AQ                      2.7262976 sec
RG                      22.37
DW                      41.600 usec
DE                      16.81 usec
TE                      298.0 K
D1                      1.00000000 sec
TD0                     1
SFO1                    600.1737060 MHz
NUC1                    1H
P0                      2.67 usec
P1                      8.00 usec
PLW1                    7.50000000 W

F2 - Processing parameters
SI                      65536
SF                      600.1700115 MHz
WDW                     EM
SSB                     0
LB                      0.30 Hz
GB                      0
PC                      1.00
```

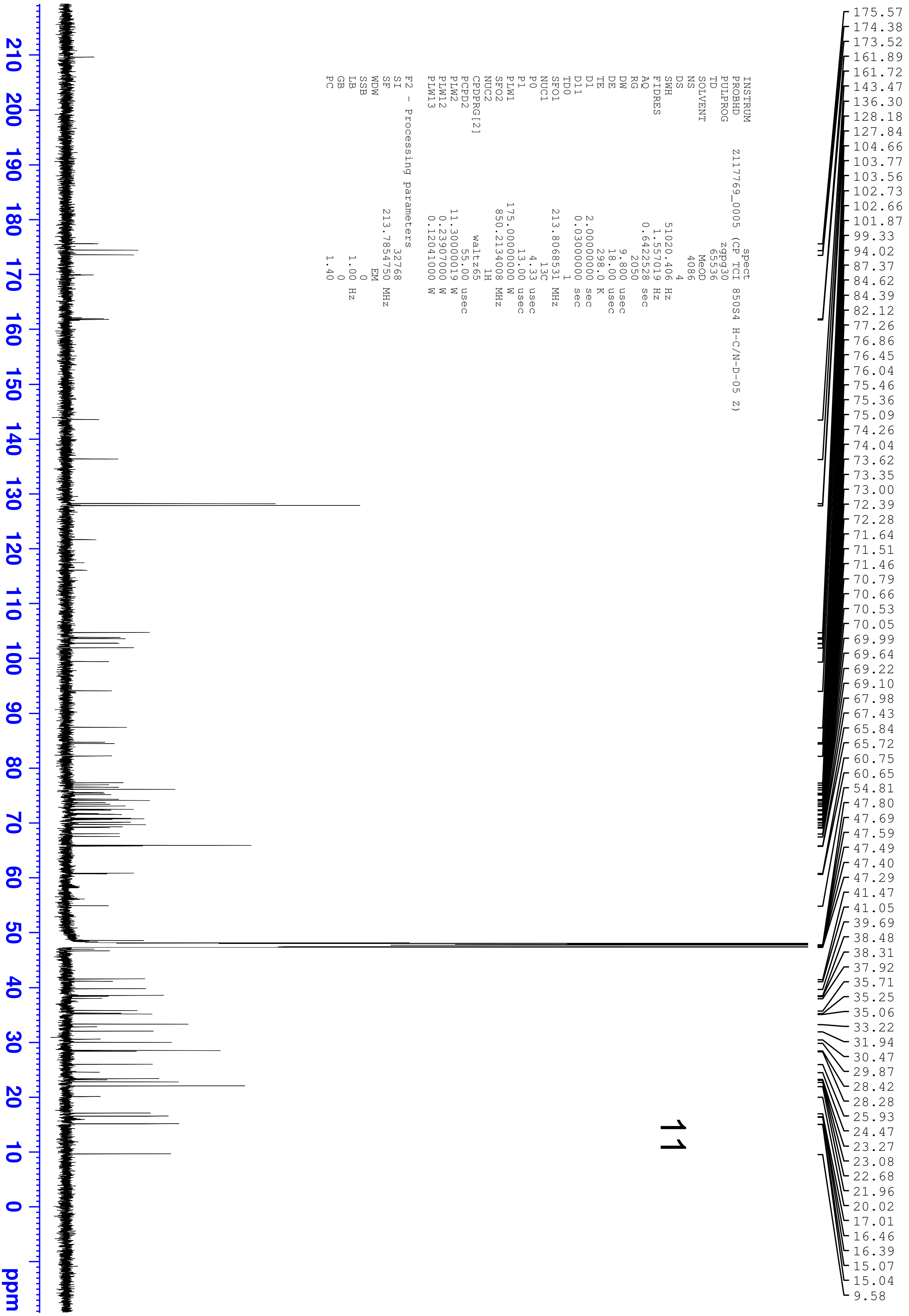
INSTRUM spect
PROBHD Z117769_0005 (CP TCI 850S4 H-C/N-D-05 Z)
PULPROG zgpg30
TD 65536
SOLVENT MeOD
NS 4086
DS 4
SWH 51020.406 Hz
FIDRES 1.557019 Hz
AQ 0.6422528 sec
RG 2050
DW 9.800 usec
DE 18.00 usec
TE 298.0 K
D1 2.00000000 sec
D11 0.03000000 sec
TD0 1
SFO1 213.8068531 MHz
NUC1 13C
P0 4.33 usec
P1 13.00 usec
PLW1 175.00000000 W
SFO2 850.2134008 MHz
NUC2 1H
CPDPRG[2] waltz65
PCPD2 55.00 usec
PLW2 11.30000019 W
PLW12 0.23907000 W
PLW13 0.12041000 W
F2 - Processing parameters
SI 32768
SF 213.7854750 MHz
WDW EM
SSB 0
LB 1.00 Hz
GB 0
PC 1.40
175.57
174.38
173.52
161.89
161.72
143.47
136.30
128.18
127.84
104.66
103.77
103.56
102.73
102.66
101.87
99.33
94.02
87.37
84.62
84.39
82.12
77.26
76.86
76.45
76.04
75.46
75.36
75.09
74.26
74.04
73.62
73.35
73.00
72.39
72.28
71.64
71.51
71.46
70.79
70.66
70.53
70.05
69.99
69.64
69.22
69.10
67.98
67.43
65.84
65.72
60.75
60.65
54.81
47.80
47.69
47.59
47.49
47.40
47.29
41.47
41.05
39.69
38.48
38.31
37.92
35.71
35.25
35.06
33.22
31.94
30.47
29.87
28.42
28.28
25.93
24.47
23.27
23.08
22.68
21.96
20.02
17.01
16.46
16.39
15.07
15.04
9.58
210
200
190
180
170
160
150
140
130
120
110
100
90
80
70
60
50
40
30
20
10
0
ppm
11

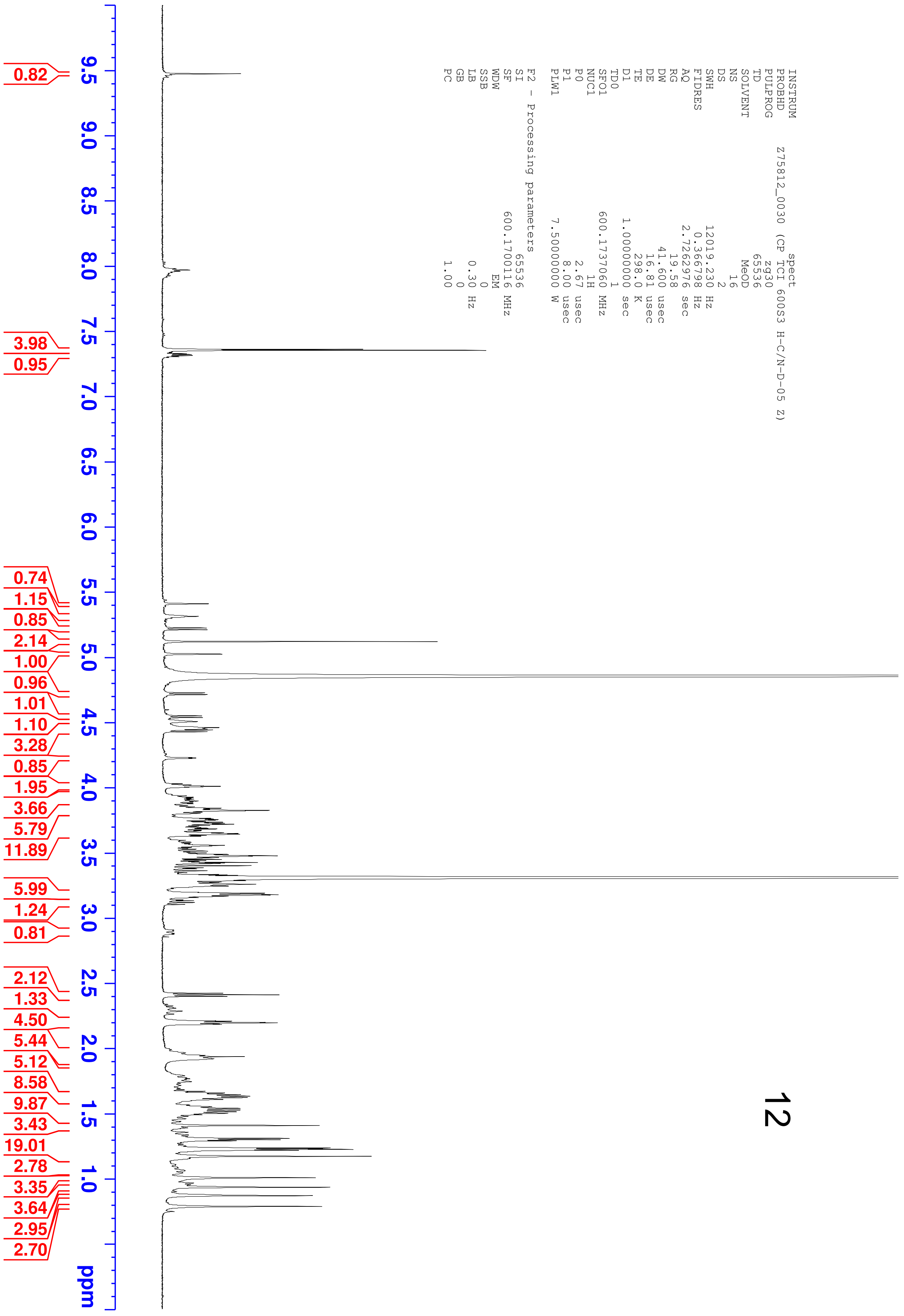
INSTRUM spect
PROBHD Z75812_0030 (CP TCI 600S3 H-C/N-D-05 Z)
PULPROG zg30
TD 65536
SOLVENT MeOD
NS 16
DS 2
SWH 12019.230 Hz
FIDRES 0.366798 Hz
AQ 2.7262976 sec
RG 19.58
DW 41.600 usec
DE 16.81 usec
TE 298.0 K
D1 1.00000000 sec
TD0 1
SFO1 600.1737060 MHz
NUC1 1H
P0 2.67 usec
P1 8.00 usec
PLW1 7.50000000 W
F2 - Processing parameters
SI 65536
SF 600.1700116 MHz
WDW EM
SSB 0
LB 0.30 Hz
GB 0
PC 1.00
12
9.5 9.0 8.5 8.0 7.5 7.0 6.5 6.0 5.5 5.0 4.5 4.0 3.5 3.0 2.5 2.0 1.5 1.0 ppm
0.82 3.98 0.95 0.74 1.15 0.85 2.14 1.00 0.96 1.01 1.10 3.28 0.85 1.95 3.66 5.79 11.89 5.99 1.24 0.81 2.12 1.33 4.50 5.44 5.12 8.58 9.87 3.43 19.01 2.78 3.35 3.64 2.95 2.70

12

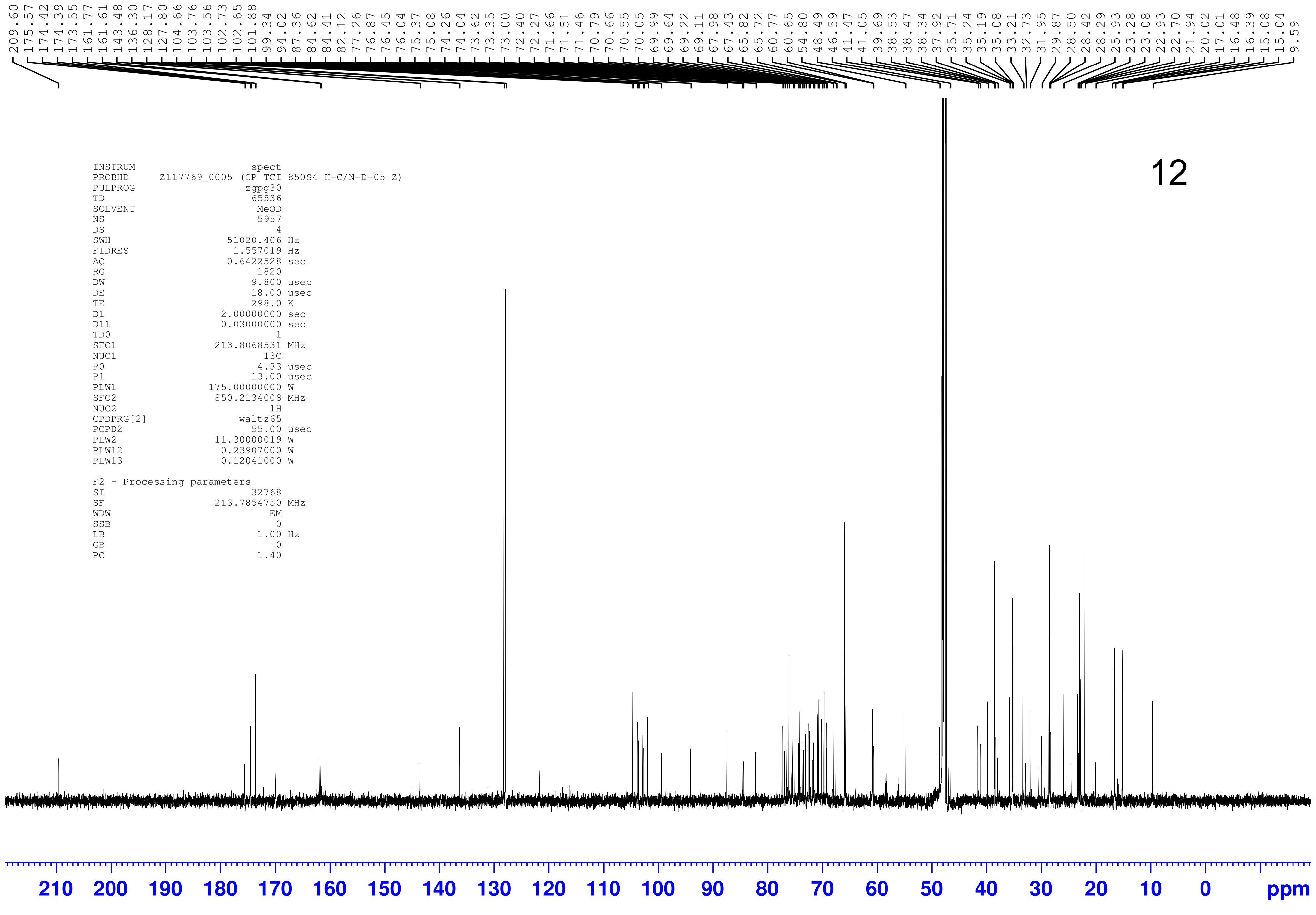
209.60
175.57
174.42
174.39
173.55
161.77
161.61
143.48
136.30
128.17
127.80
104.66
103.76
103.56
102.73
102.65
101.88
99.34
94.02
87.36
84.62
84.41
82.12
77.26
76.87
76.45
76.04
75.37
75.08
74.26
74.04
73.62
73.35
73.00
72.40
72.27
71.66
71.51
71.46
70.79
70.66
70.55
70.05
69.99
69.64
69.22
69.11
67.98
67.43
65.82
65.72
60.77
60.65
54.80
48.49
46.59
41.47
41.05
39.69
38.53
38.47
38.34
37.92
35.71
35.24
35.19
35.08
33.21
32.73
31.95
29.87
28.50
28.42
28.29
25.93
23.28
23.08
22.93
22.70
21.94
20.02
17.01
16.48
16.39
15.08
15.04
9.59
INSTRUM spect
PROBHD Z117769_0005 (CP TCI 850S4 H-C/N-D-05 Z)
PULPROG zgpg30
TD 65536
SOLVENT MeOD
NS 5957
DS 4
SWH 51020.406 Hz
FIDRES 1.557019 Hz
AQ 0.6422528 sec
RG 1820
DW 9.800 usec
DE 18.00 usec
TE 298.0 K
D1 2.00000000 sec
D11 0.03000000 sec
TD0 1
SFO1 213.8068531 MHz
NUC1 13C
P0 4.33 usec
P1 13.00 usec
PLW1 175.00000000 W
SFO2 850.2134008 MHz
NUC2 1H
CPDPRG[2] waltz65
PCPD2 55.00 usec
PLW2 11.30000019 W
PLW12 0.23907000 W
PLW13 0.12041000 W
F2 - Processing parameters
SI 32768
SF 213.7854750 MHz
WDW EM
SSB 0
LB 1.00 Hz
GB 0
PC 1.40
210
200
190
180
170
160
150
140
130
120
110
100
90
80
70
60
50
40
30
20
10
0
ppm

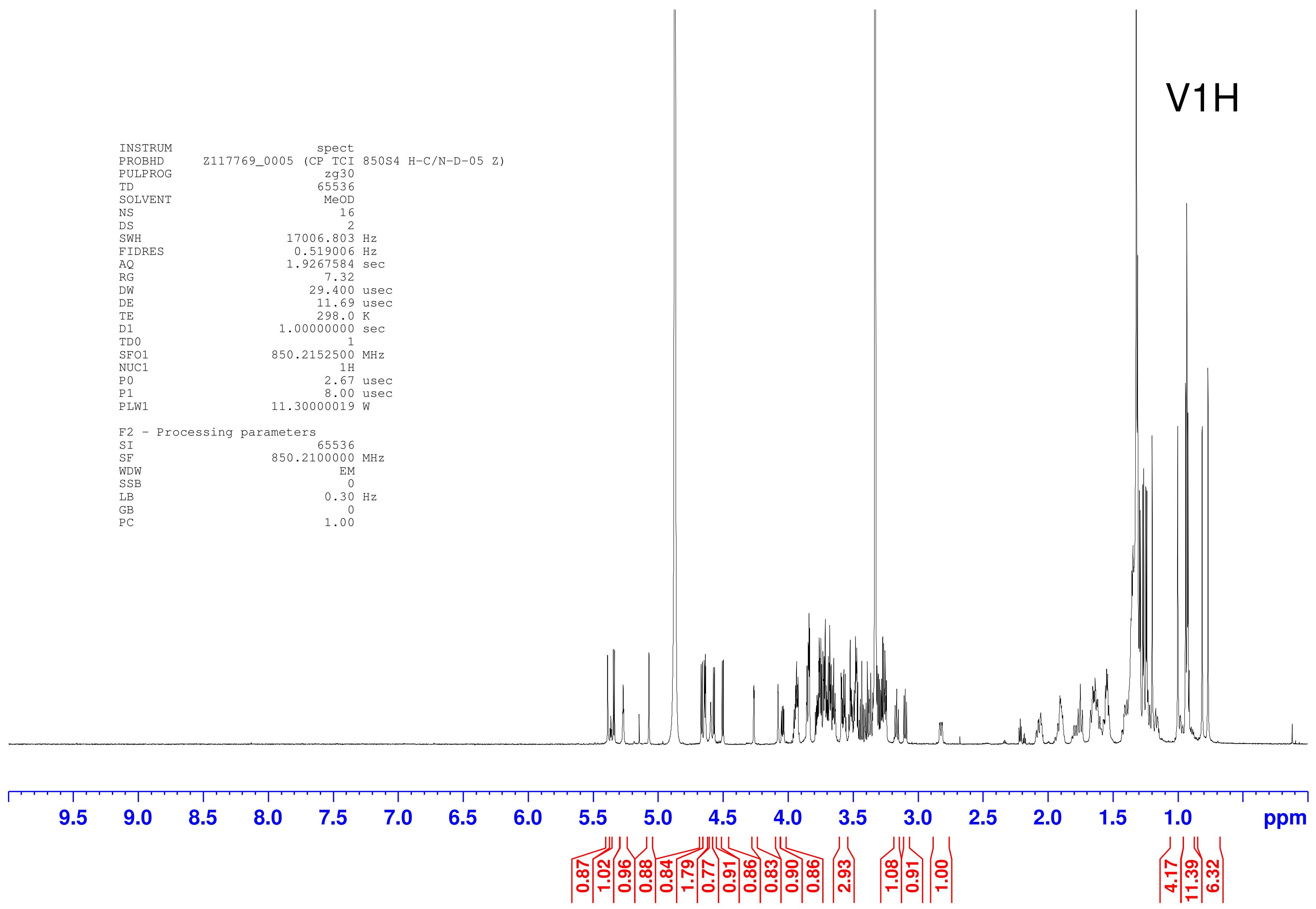
V1H
INSTRUM spect
PROBHD Z117769_0005 (CP TCI 850S4 H-C/N-D-05 Z)
PULPROG zg30
TD 65536
SOLVENT MeOD
NS 16
DS 2
SWH 17006.803 Hz
FIDRES 0.519006 Hz
AQ 1.9267584 sec
RG 7.32
DW 29.400 usec
DE 11.69 usec
TE 298.0 K
D1 1.00000000 sec
TD0 1
SFO1 850.2152500 MHz
NUC1 1H
P0 2.67 usec
P1 8.00 usec
PLW1 11.30000019 W
F2 - Processing parameters
SI 65536
SF 850.2100000 MHz
WDW EM
SSB 0
LB 0.30 Hz
GB 0
PC 1.00
9.5 9.0 8.5 8.0 7.5 7.0 6.5 6.0 5.5 5.0 4.5 4.0 3.5 3.0 2.5 2.0 1.5 1.0 ppm
0.87 1.02 0.96 0.88 0.84 1.79 0.77 0.91 0.86 0.83 0.90 0.86 2.93 1.08 0.91 1.00 4.17 11.39 6.32

V1H

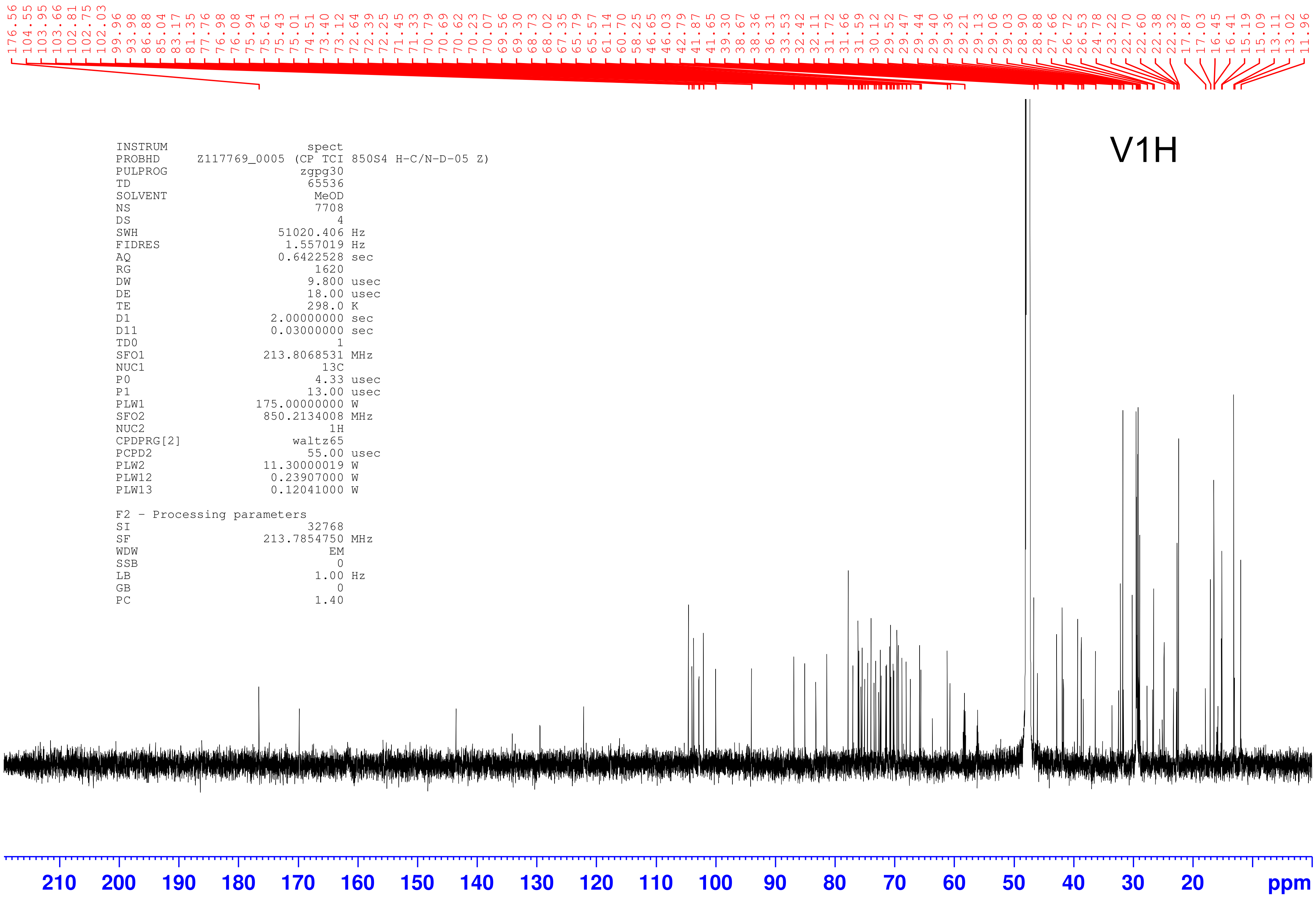

INSTRUM	spect
PROBHD	Z117769_0005 (CP TCI 850S4 H-C/N-D-05 Z)
PULPROG	zgpg30
TD	65536
SOLVENT	MeOD
NS	7708
DS	4
SWH	51020.406 Hz
FIDRES	1.557019 Hz
AQ	0.6422528 sec
RG	1620
DW	9.800 usec
DE	18.00 usec
TE	298.0 K
D1	2.00000000 sec
D11	0.03000000 sec
TD0	1
SFO1	213.8068531 MHz
NUC1	13C
P0	4.33 usec
P1	13.00 usec
PLW1	175.00000000 W
SFO2	850.2134008 MHz
NUC2	1H
CPDPRG[2]	waltz65
PCPD2	55.00 usec
PLW2	11.30000019 W
PLW12	0.23907000 W
PLW13	0.12041000 W

F2 - Processing parameters

SI	32768
SF	213.7854750 MHz
WDW	EM
SSB	0
LB	1.00 Hz
GB	0
PC	1.40

V2H

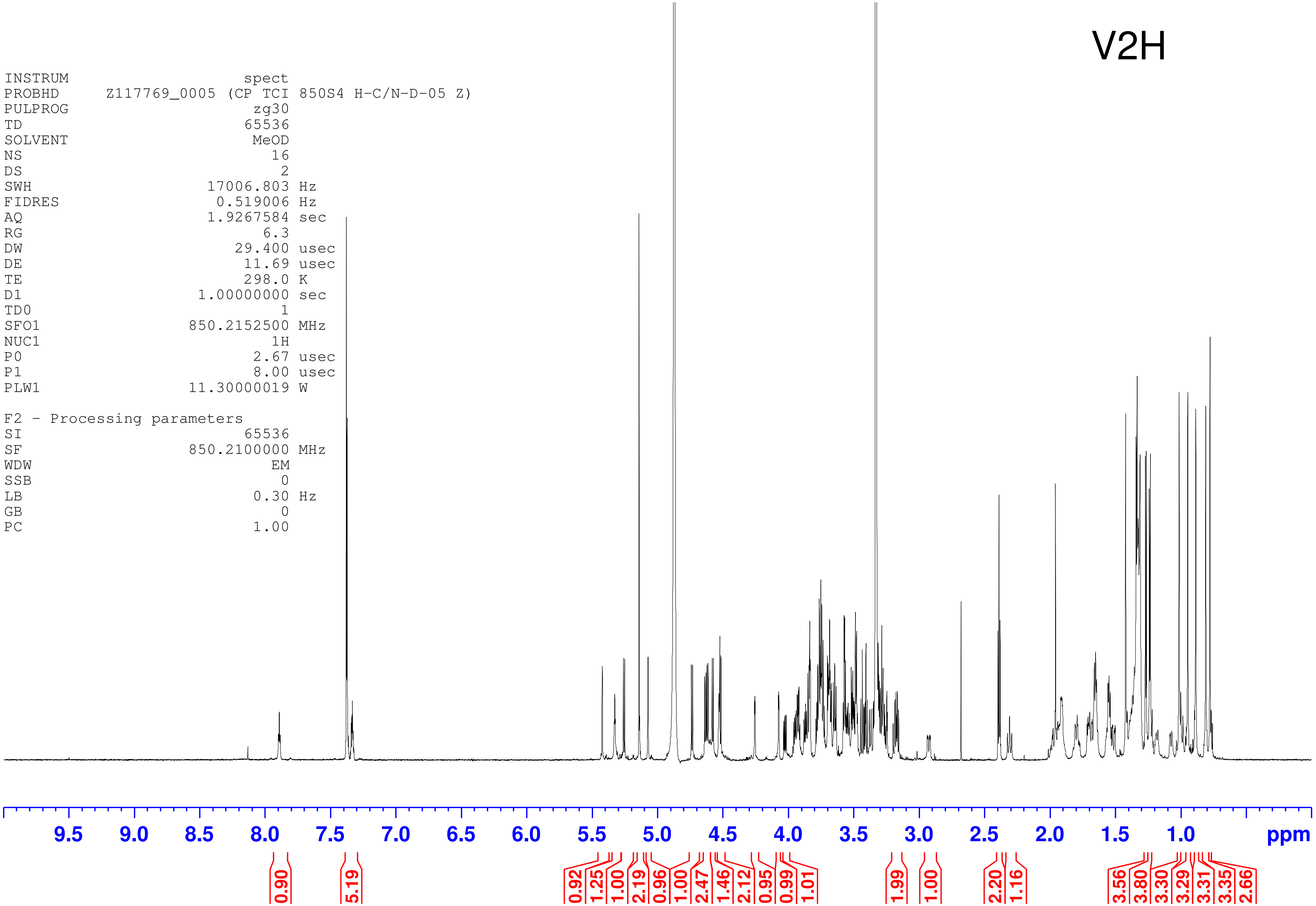
INSTRUM spect
PROBHD Z117769_0005 (CP TCI 850S4 H-C/N-D-05 Z)
PULPROG zg30
TD 65536
SOLVENT MeOD
NS 16
DS 2
SWH 17006.803 Hz
FIDRES 0.519006 Hz
AQ 1.9267584 sec
RG 6.3
DW 29.400 usec
DE 11.69 usec
TE 298.0 K
D1 1.00000000 sec
TD0 1
SFO1 850.2152500 MHz
NUC1 1H
P0 2.67 usec
P1 8.00 usec
PLW1 11.30000019 W
F2 - Processing parameters
SI 65536
SF 850.2100000 MHz
WDW EM
SSB 0
LB 0.30 Hz
GB 0
PC 1.00
9.5 9.0 8.5 8.0 7.5 7.0 6.5 6.0 5.5 5.0 4.5 4.0 3.5 3.0 2.5 2.0 1.5 1.0 ppm
0.90
5.19
0.92 1.25 1.00 2.19 0.96 1.00 2.47 1.46 2.12 0.95 0.99 1.01
1.99 1.00
2.20 1.16
3.56 3.80 3.30 3.29 3.31 3.35 2.66

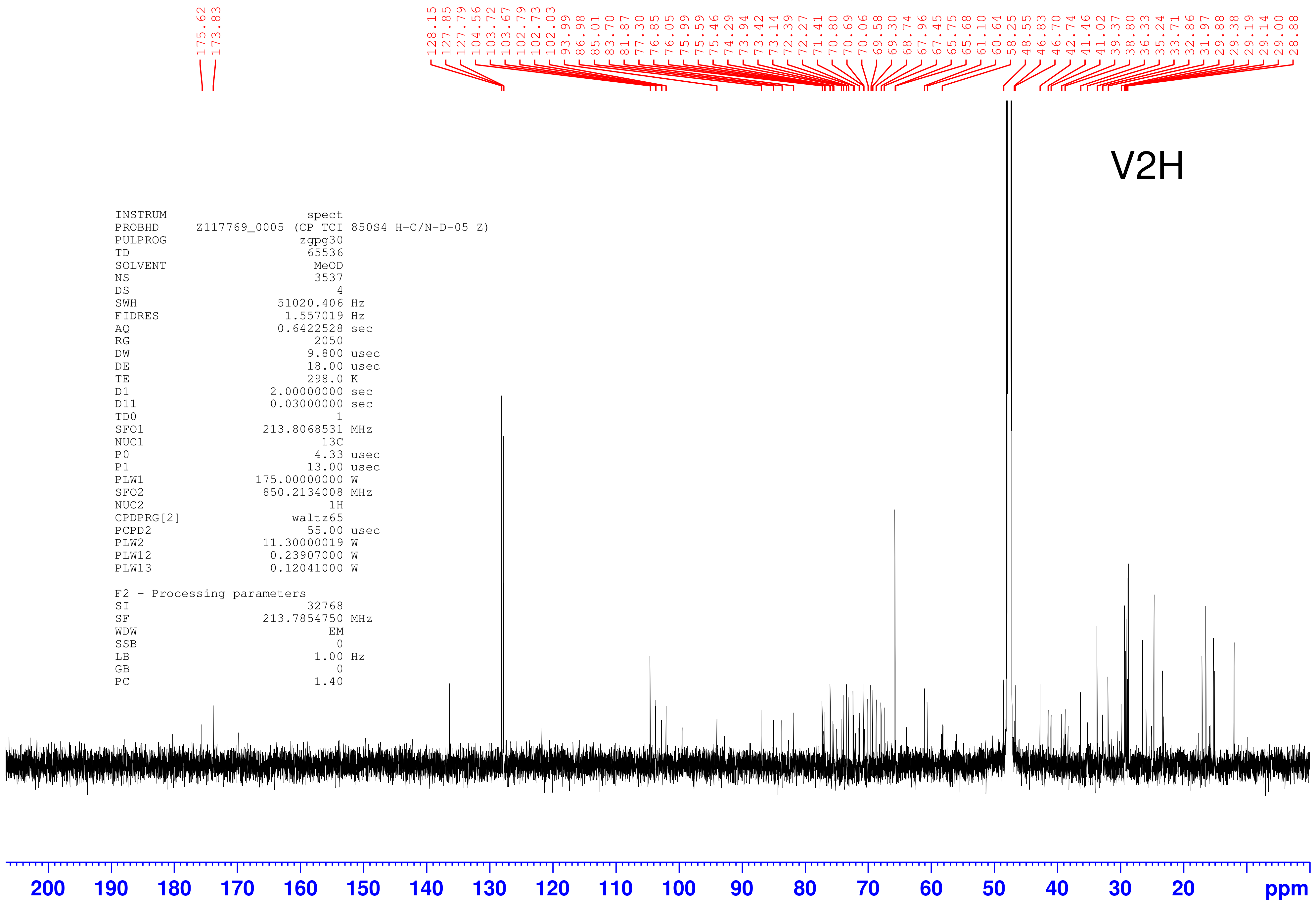
175.62
173.83
128.15
127.85
127.79
104.56
103.72
103.67
102.79
102.73
102.03
93.99
86.98
85.01
83.70
81.87
77.30
76.85
76.05
75.99
75.59
75.46
74.29
73.94
73.42
73.14
72.39
72.27
71.41
70.80
70.69
70.06
69.58
69.30
68.74
67.96
67.45
65.75
65.68
61.10
60.64
58.25
48.55
46.83
46.70
42.74
41.46
41.02
39.37
38.80
36.33
35.24
33.71
32.86
31.97
29.88
29.38
29.19
29.14
29.00
28.88
V2H
INSTRUM spect
PROBHD Z117769_0005 (CP TCI 850S4 H-C/N-D-05 Z)
PULPROG zgpg30
TD 65536
SOLVENT MeOD
NS 3537
DS 4
SWH 51020.406 Hz
FIDRES 1.557019 Hz
AQ 0.6422528 sec
RG 2050
DW 9.800 usec
DE 18.00 usec
TE 298.0 K
D1 2.00000000 sec
D11 0.03000000 sec
TD0 1
SFO1 213.8068531 MHz
NUC1 13C
P0 4.33 usec
P1 13.00 usec
PLW1 175.00000000 W
SFO2 850.2134008 MHz
NUC2 1H
CPDPRG[2] waltz65
PCPD2 55.00 usec
PLW2 11.30000019 W
PLW12 0.23907000 W
PLW13 0.12041000 W
F2 - Processing parameters
SI 32768
SF 213.7854750 MHz
WDW EM
SSB 0
LB 1.00 Hz
GB 0
PC 1.40
200
190
180
170
160
150
140
130
120
110
100
90
80
70
60
50
40
30
20
ppm
